# Rhenium(I) conjugates as tools for tracking cholesterol in cells

**DOI:** 10.1093/mtomcs/mfac040

**Published:** 2022-06-03

**Authors:** Joanna Lazniewska, Christie Bader, Shane M Hickey, Stavros Selemidis, John O'Leary, Peter V Simpson, Stefano Stagni, Sally E Plush, Massimiliano Massi, Doug Brooks

**Affiliations:** UniSA Clinical and Health Sciences, University of South Australia, Adelaide, South Australia 5001, Australia; UniSA Clinical and Health Sciences, University of South Australia, Adelaide, South Australia 5001, Australia; UniSA Clinical and Health Sciences, University of South Australia, Adelaide, South Australia 5001, Australia; Department of Human Biosciences, RMIT University, Melbourne, Victoria 3000, Australia; Discipline of Histopathology, University of Dublin Trinity College, Dublin 2, Ireland; School of Molecular and Life Sciences - Curtin University, Bentley, Western Australia 6102, Australia; Department of Industrial Chemistry Toso Montanari, University of Bologna, Via Zamboni, 33, Bologna I-40136, Italy; UniSA Clinical and Health Sciences, University of South Australia, Adelaide, South Australia 5001, Australia; School of Molecular and Life Sciences - Curtin University, Bentley, Western Australia 6102, Australia; UniSA Clinical and Health Sciences, University of South Australia, Adelaide, South Australia 5001, Australia; Discipline of Histopathology, University of Dublin Trinity College, Dublin 2, Ireland; School of Molecular and Life Sciences - Curtin University, Bentley, Western Australia 6102, Australia

**Keywords:** Cholesterol, endosomal pathway, luminescence, prostate cancer, rhenium(I) complex

## Abstract

Cholesterol is vital to control membrane integrity and fluidity, but is also a precursor to produce steroid hormones, bile acids, and vitamin D. Consequently, altered cholesterol biology has been linked to many diseases, including metabolic syndromes and cancer. Defining the intracellular pools of cholesterol and its trafficking within cells is essential to understand both normal cell physiology and mechanisms of pathogenesis. We have synthesized a new cholesterol mimic (**ReTEGCholestanol**), comprising a luminescent rhenium metal complex and a cholestanol targeting unit, linked using a tetraethylene glycol (TEG) spacer. **ReTEGCholestanol** demonstrated favourable imaging properties and improved water solubility when compared to a cholesterol derivative, and structurally related probes lacking the TEG linker. A non-malignant and three malignant prostate cell lines were used to characterize the uptake and intracellular distribution of **ReTEGCholestanol**. The **ReTEGCholestanol** complex was effectively internalized and mainly localized to late endosomes/lysosomes in non-malignant PNT1a cells, while in prostate cancer cells it also accumulated in early endosomes and multivesicular bodies, suggesting disturbed cholesterol biology in the malignant cells. The **ReTEGCholestanol** is a novel imaging agent for visualizing endosomal uptake and trafficking, which may be used to define cholesterol related biology including membrane integration and altered lipid trafficking/processing.

## Graphical Abstract

**Graphical Abstract:**
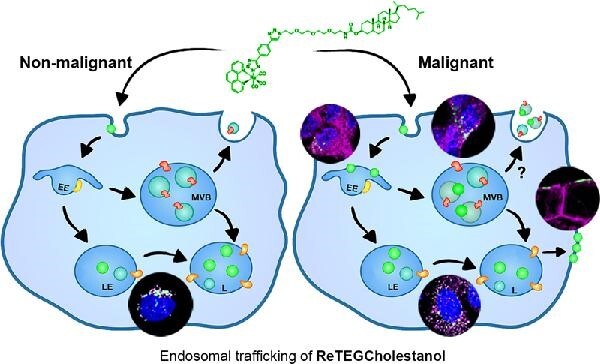
Endosomal trafficking of **ReTEGCholestanol** in non-malignant and malignant prostate cells. EE, early endosomes; LE, late endosomes; L, lysosomes; MVB, multivesicular bodies.

## Introduction

Cholesterol is an essential multifunctional lipid, which is involved in the control of eukaryotic cell membrane stability and permeability. As well as its structural function in lipid rafts and biological membranes, cholesterol is a precursor for steroid hormones,^1^ bile acid,^2^ and vitamin D^3^ production and is critical for signal transduction, membrane trafficking, endocytosis, and lipid metabolism.^4^ Cholesterol can be synthesized *de novo* in the liver or obtained from the diet/extracellular environment. The tight regulation of intracellular cholesterol is important for long term cellular function/homeostasis and organism health.^4^ The uptake of exogenous cholesterol into cells is primarily facilitated *via* endocytosis with low-density lipoproteins (LDL), which carry cholesterol that can then be internalized *via* LDL receptors at the plasma membrane.^5^ These receptors are internalized *via* clathrin-dependent endocytosis and trafficked towards the lysosome *via* the endosomal network.^5^ In the late endosome-lysosome, cholesterol is released from LDL allowing insertion into the membranes of these compartments.^5^ Cholesterol pools can subsequently be redistributed to autophagosomes, the plasma membrane or the endoplasmic reticulum, possibly *via* a process involving phosphatidylserine.^6,7^ At the endoplasmic reticulum, cholesterol can be esterified to permit lipid droplet storage or redistributed to other membrane pools. There are numerous mechanisms of cholesterol uptake/transport and specific sites for intracellular cholesterol distribution/storage, which align with the multifunctionality of this important lipid.

Alterations in cholesterol homeostasis can be observed in a variety of human pathologies at the cellular and organ level, making it a molecule of particular interest for medical research. The amount of cholesterol is generally higher in cancer cells,^8^ which may be the result of sequestering excess exogenous cholesterol^9,10^ or increased intracellular synthesis/lipid degradation.^11^ Because of the multiple functions and bioproducts of cholesterol, there are many contradicting reports on cholesterol function in cancer cell survival and progression.^8,9^ Difficulties in tracking and detecting cholesterol and its derivatives has limited our understanding of the exact changes in the distribution and functional significance of cholesterol in cancer cells and for other diseases. The focus for cholesterol biology has been mainly on the molecular machinery involved in its synthesis and/or trafficking but is still limited due to the difficulties in detecting cholesterol itself.

A range of fluorescent sterol mimics and tagged sterols have been described and used to investigate cholesterol cell biology, but these have come with significant drawbacks and challenges. Dehydroergosterol and cholestatrienol are both intrinsically fluorescent sterols, which have very similar structures to cholesterol, providing potential biochemical/cell biological mimics.^12,13^ However, the fluorescence properties of these compounds are not ideal for microscopy, with low quantum yields, poor photostability, absorption bands within the UV range, and emissions between 370 nm and 400 nm.^14,15^ This fluorescent profile is outside the range of confocal microscopy and is at the upper excitation and emission limits of standard epifluorescence microscopes, thus requiring specialized objectives for efficient transmission of the emitted light.^12^ Multiphoton microscopy, can overcome some of these limitations, providing the additional resolution for interrogating subcellular localization and equipment sensitivity for photon detection, while employing low excitation frequencies, which reduces sample damage induced by UV excitation.^16,17^ Utilization of these molecules for live cell imaging requires complexing with methyl-β-cyclodextrin (MβCD), due to their poor solubility; and this process is time consuming and requires careful storage once prepared.^12,18^ Thus, despite their usefulness as a biological and biochemical mimics of cholesterol, dehydroergosterol and cholestatrienol, require specialized equipment and expertise to be used successfully, which has limited their use.^12^

The inherent difficulties in the application of fluorescent sterols have driven a need for alternative approaches to imaging cholesterol in cells. Most live cell imaging studies have utilized fluorescently tagged sterols, such as BODIPY-tagged cholesterol analogs, which do have improved fluorescence properties.^19^ Numerous fluorescently tagged analogs have been developed, of which cholesterol with BODIPY tagged at C24 is one of the most representative mimics of native cholesterol, demonstrating similar membrane partitioning, metabolism, and trafficking in cells^18,20–23^ However, some processing, such as cholesterol partitioning into different phases of model membranes, is altered due to the presence of the BODIPY tag and this molecule can also suffer from self-quenching.^18^ Tagged analogs with fluorophores including rhodamine and dansyl attached at C6 of cholestanol have also been developed and have shown similar cellular localization and membrane partitioning as C24 tagged cholesterols, providing alternative tools for investigating cholesterol trafficking in cells.^24–26^ However, dansyl has low emission and is prone to photobleaching, and thus is less ideal for live cell imaging, while the rhodamine tag impairs endoplasmic reticulum trafficking and esterification.^24–26^ Fluorescently tagged cholesterol that utilizes the 3’ OH as the point of conjugation have also produced viable tools for the investigation of cholesterol trafficking and are more synthetically accessible than their C6 and C24 counterparts.^21,27,28^ BODIPY attached to cholesterol in this position, using an ester linkage, demonstrates a similar cellular distribution to the C24 functionalized derivative, but offers improved cellular uptake without the need for complexing with MβCD.^21,27^ Although functionalization through the 3-OH region disallows probes of this design to be used to track esterified cholesterol, the benefit of synthetic simplicity in this approach allows the addition of a range of alternative fluorescence tags to suit an investigator's specific imaging requirements. Consequently, there is still a defined need for other cholesterol derivatives that are readily accessible and offer advantageous imaging properties.

Luminescent organometallic rhenium complexes can provide alternatives to traditional organic fluorescent tags with a number of specific advantages. For example, they have reduced photobleaching, allowing for longer imaging times; a large Stokes shift, reduced background for the excitation beam; and long emission lifetimes, making time-gating of bright endogenous fluorescence possible.^29,30^ These compounds are also compatible with multimodal imaging platforms, enabling detection though multiphoton microscopy,^31^ infrared spectroscopy,^32–34^ Raman spectroscopy,^33,35^ X-ray fluorescence spectroscopy,^36,37^ mass spectrometry,^29,38^ and potentially electron microscopy. In order to exploit these properties, we have synthesized four new cholesterol related compounds, tagged with a neutral rhenium complex of the type *fac*-[Re(CO)_3_(phen)L], where phen = 1,10-phenathroline and L is a tetrazolato ancillary ligand, and have explored their potential as cellular imaging agents. These initial complexes have used the OH handle as the point of attachment for both cholesterol and cholestanol, as structural mimics of cholesterol.^24,39^ We also explore the inclusion of a short tetraethylene glycol (TEG) linker to improve water solubility thereby removing the need for further complexing with MβCD for cellular uptake and delivery. Here, we have developed an alternative tool for the investigation of cholesterol trafficking in cells, with the synthesis of **ReTEGCholestanol** that demonstrates good aqueous solubility and photophysical properties amenable for live cell imaging.

## Materials and methods

### General experimental details

Chemicals were purchased from commercial sources and used without further purification. Anhydrous CH_2_Cl_2_ and THF were obtained by drying over freshly activated 3 Å molecular sieves. Anhydrous DMF and EtOH were purchased from Merck (Australia). Thin layer chromatography (TLC) was performed on silica gel 60 F_254_ plates purchased from Merck (Australia). Column chromatography was performed using silica gel as the stationary phase unless otherwise stated. All melting points were obtained using Stuart Scientific SMP10 melting point apparatus and were uncorrected. All ^1^H and ^13^C NMR spectra were collected on either a BRUKER AVANCE III 500 MHz FT-NMR spectrometer or BRUKER AVANCE 400 MHz FT-NMR spectrometer. All NMR experiments were performed at 25°C. Samples were dissolved in either CDCl_3_, DMSO-*d_6_*, or acetone-*d_6_* with the residual solvent peak used as the internal reference—CDCl_3_: 7.26 (^1^H) and 77.0 (^13^C); DMSO-*d_6_*: 2.50 (^1^H) and 39.52 (^13^C); acetone-*d_6_*: 2.05 (^1^H) and 29.84 (^13^C).^40^ Proton spectra are reported as chemical shift δ (ppm) (integral, multiplicity (s = singlet, br s = broad singlet, d = doublet, t = triplet and m = multiplet), coupling constant (Hz)). High resolution mass spectral data was collected using an AB SCIEX TripleTOF 5600 mass spectrometer in a MeOH solvent system containing 0.1% formic acid. Analyte solutions were prepared in a 1:1 mixture of HPLC grade MeCN and MeOH (conc. ∼ 1 mg mL^−1^). RP-HPLC experiments were conducted on a Shimadzu Prominence UltraFast Liquid Chromatography (UFLC) system equipped with a CBM-20A communications bus module, a DGU-20ASR degassing unit, an LC-20AD liquid chromatograph pump, an SIL-20AHT autosa-sampler, and SPD-M20A photo diode array detector, a CTO-20A column oven and a Phenomenex Kinetex 5 mM C18 100 Å 250 mm × 4.60 mm column. The solvent system used was a gradient beginning at 5% MeOH in H_2_O containing 0.1% formic acid over 9 min, followed by 95% MeOH in H_2_O containing 0.1% formic acid over 21 min. A flow rate of 1 mL/min was maintained throughout. UV-vis spectra were recorded over the range 250—750 nm on a Varian Cary 50 UV-vis spectrophotometer (Agilent) in a 10 mm pathlength quartz cuvette, using a scan rate of 9,600 nm/min. Fluorescence spectra were recorded on a Varian Cary Eclipse spectrophotometer (Agilent) over 450—750 nm. An excitation slit width of 10 nm and emission slit width of 5 nm, PMT voltage of 600 V, scan rate of 600 nm/min, a 10 mm pathlength quartz cuvette and an excitation wavelength of 405 nm was used for all experiments. Background correction was applied to all UV-Vis and fluorescence experiments using a solvent blank. All samples were performed at a final concentration of 1 × 10^−^^5^ M in a 1% DMSO in PBS solution. The synthesis of **Realkyne** has been previously reported.^38^.^38^  ^1^H and ^13^C NMR spectra for all compounds and HRMS and normalized absorbance and emission spectra for all Re complexes are included in the Supplementary Materials.

### Synthesis

#### Synthesis of 3β-methanesulfonyloxycholest-5-ene **1**

To a stirred solution of cholesterol (3.301 g, 8.54 mmol) and anhydrous CH_2_Cl_2_ (85 mL) at 0°C under an inert atmosphere, was added Et_3_N (2.4 mL, 17.22 mmol) and MsCl (730 μL, 9.43 mmol) dropwise. The reaction was warmed to ambient temperature and stirred for 4 h before being diluted with CH_2_Cl_2_ (40 mL) and transferred to a separatory funnel. The mixture was washed with sat. NaHCO_3_ (40 mL), H_2_O (40 mL), brine (40 mL), dried (Na_2_SO_4_), filtered, and concentrated *in vacuo* to give an off-white solid. The crude material was purified using a silica plug (20% EtOAc in pet. spirits) to give the title compound (3.403 g, 86%) as a white solid. *R_f_* = 0.40 (20% EtOAc in pet. spirits). m.p 122–124°C. (lit. 118–119°C).^40^  ^1^H NMR (500 MHz, CDCl_3_) δ 5.42 (1H, d, *J* = 2.0 Hz), 4.89–4.55 (1H, m), 3.00 (3H, s), 2.55–2.48 (2H, m), 2.02–1.75 (6H, m), 1.60–0.85 (32H, m), 0.67 (3H, s). ^13^C NMR (125 MHz, CDCl_3_) δ 138.8, 124.0, 82.2, 56.8, 56.3, 50.1, 42.4, 39.8, 39.6, 39.3, 38.9, 37.0, 36.5, 36.3, 35.9, 32.0, 31.9, 29.1, 28.3, 28.2, 24.4, 24.0, 23.0, 22.7, 21.2, 19.3, 18.9, 12.0.

#### Synthesis of 3β-azido-5-cholestene **2**

To a stirred solution of mesylate **1** (3.365 g, 7.24 mmol) and anhydrous CH_2_Cl_2_ (70 mL) under an inert atmosphere, was added TMSN_3_ (1.2 mL, 8.29 mmol) and BF_3_·(OEt_2_)_2_ (2.3 mL, 18.64 mmol). The reaction was stirred for 16 h before being poured into a stirred solution of 2 M NaOH (20 mL). The crude material was extracted with CH_2_Cl_2_ (3 × 50 mL). The combined organic portions were washed with brine (50 mL), dried (Na_2_SO_4_), filtered, and concentrated *in vacuo* to give a yellow oil. The crude material was purified using column chromatography (pet. spirits) to give the title compound (1.802 g, 60%) as a white solid. *R_f_* = 0.27 (pet. spirits). m.p 87–88°C. (lit. 84–86°C).^40^  ^1^H NMR (500 MHz, CDCl_3_) δ 5.39 (1H, d, *J* = 4.1 Hz), 3.24–3.17 (1H, m), 2.29 (2H, d, *J* = 7.9 Hz), 2.03–1.96 (2H, m), 1.92–1.79 (3H, m), 1.60–1.23 (12H, m), 1.19–0.86 (21H, m), 0.68 (3H, s). ^13^C NMR (125 MHz, CDCl_3_) δ 140.0, 122.7, 61.3, 56.9, 56.3, 50.2, 42.5, 39.9, 39.7, 38.3, 37.7, 36.8, 36.3, 35.9, 32.02, 31.96, 28.4, 28.2, 28.1, 24.4, 24.0, 23.0, 22.7, 21.1, 19.4, 18.9, 12.0. HRMS (ESI, *m/z*) for C_27_H_45_N_3_ [M + H—N_2_]^+^ calc. 384.3625; found 384.3616.

### ReCholesterol

**Realkyne** (66 mg, 0.11 mmol) and **2** (44.0 mg, 0.11 mmol) were added to 10.0 mL of a 3:1 THF/H_2_O mixture, along with CuSO_4_∙5H_2_O (6 mg, 0.0213 mmol) and sodium ascorbate (2 mg, 0.016 mmol). The solution was heated to 60°C and stirred for 2 d. After this time, H_2_O (20 mL) and CH_2_Cl_2_ (20 mL) were added. The organic layer was extracted and dried over anhydrous MgSO_4_. After removal of the solvent, the crude product was purified using column chromatography (neutral Brockmann II alumina as stationary phase) using CH_2_Cl_2_ as eluent, to give the target compound as a yellow solid (63 mg, 55%). *R_f_* = 0.51 (CH_2_Cl_2_). ^1^H NMR (500 MHz, DMSO-*d_6_*) δ 9.58 (2H, dd, *J* = 5.2, 1.4 Hz), 9.00 (2H, dd, *J* = 8.3, 1.5 Hz), 8.71 (1H, s), 8.32 (2H, s), 8.14 (2H, dd, *J* = 8.3, 5.2 Hz), 7.76 (2H, d, *J* = 8.7 Hz), 7.57 (2H, d, *J* = 8.7 Hz), 5.44 (1H, br s), 4.42–4.36 (1H, m), 2.82–2.74 (1H, m), 2.11–1.94 (5H, m), 1.86–1.73 (1H, m), 1.59–0.96 (23H, m), 0.90 (3H, d, *J* = 6.6 Hz), 0.86–0.84 (6H, m), 0.67 (3H, s). ^13^C NMR (125 MHz, DMSO-d_6_) δ 196.7, 161.4, 153.6, 146.5, 145.6, 140.2, 139.1, 131.0, 130.3, 129.3, 127.7, 126.7, 126.0, 125.2, 123.0, 120.2, 60.0, 56.6, 55.6, 51.7, 42.6, 36.9, 36.2, 35.6, 35.2, 31.3, 29.4, 27.8, 26.7, 25.3, 23.2, 22.7, 22.4, 20.5, 19.0, 18.6, 12.7. HRMS (ESI, *m/z*) for C_51_H_58_N_9_O_3_Re [M + H]^+^ calc. 1032.4293; found 1032.4319.

#### Synthesis of 3α-bromo-5α-cholestane **3**

To a stirred solution of 5α-cholestan-3β-ol (3.106 g, 7.99 mmol), PPh_3_ (2.962 g, 11.29 mmol), and CH_2_Cl_2_ (40 mL), was added CBr_4_ (3.200 g, 9.65 mmol) slowly. The mixture was stirred at ambient temperature for 16 h over which time the colour turned light brown. The reaction mixture was concentrated under reduced pressure and the crude material was purified using a silica plug (10% EtOAc in pet. spirits) to give the desired product (3.583 g, 99%) as a white solid. *R_f_* = 0.81 (10% EtOAc in pet. spirits). m.p 102–103°C. (lit. 102–104°C).^41^  ^1^H NMR (500 MHz, CDCl_3_) δ 4.74–4.73 (1H, m), 1.99–1.93 (3H, m), 1.85–1.64 (5H, m), 1.58–1.46 (5H, m), 1.38–1.28 (4H, m), 1.27–0.94 (13H, m), 0.91–0.78 (13H, m), 0.65 (3H, s). ^13^C NMR (125 MHz, CDCl_3_) δ 56.5, 56.3, 56.2, 54.0, 42.7, 40.2, 40.1, 39.6, 37.4, 36.33, 36.27, 35.9, 35.5, 33.0, 31.9, 31.1, 28.3, 28.1, 28.0, 24.3, 24.0, 22.9, 22.7, 20.9, 18.8, 12.4, 12.2.

#### Synthesis of 3β-azido-5α-cholestane **4**

A solution of bromo **3** (455 mg, 1.01 mmol), NaN_3_ (332 mg, 5.11 mmol), and DMF (10 mL) was stirred for 16 h at 100°C. The mixture was concentrated to approximately a third of its volume under a stream of air and then diluted with EtOAc (20 mL). The organic phase was washed with H_2_O (3 × 10 mL), brine (10 mL), dried (Na_2_SO_4_), filtered, and concentrated *in vacuo* to give a yellow oil. The material was purified using column chromatography (pet. spirits) to give the desired product (258 mg, 62%) as a white, waxy solid. *R_f_* = 0.29 (pet. spirits). m.p 63–64°C. (lit. 65–67°C).^41^  ^1^H NMR (500 MHz, CDCl_3_) δ 3.29–3.22 (1H, m), 1.98–1.95 (1H, m), 1.84–1.74 (3H, m), 1.69–1.64 (1H, m), 1.60–1.43 (5H, m), 1.39–1.19 (10H, m), 1.14–0.94 (10H, m), 0.90–0.85 (10H, m), 0.80 (3H, s), 0.65 (3H, s). ^13^C NMR (125 MHz, CDCl_3_) δ 60.8, 56.6, 56.4, 54.4, 45.4, 42.7, 40.1, 39.7, 37.3, 36.3, 35.9, 35.7, 35.6, 34.2, 32.1, 28.8, 28.4, 28.2, 27.8, 24.3, 24.0, 23.0, 22.7, 21.3, 18.8, 12.4, 12.2. HRMS (ESI, *m/z*) for C_27_H_47_N_3_ [M + H—N_2_]^+^ calc. 386.3781; found 386.3787.

### ReCholestanol

**Realkyne** (59 mg, 0.092 mmol) and **4** (38 mg, 0.092 mmol) were added to 10.0 mL of a 3:1 THF/H_2_O mixture, along with CuSO_4_∙5H_2_O (5 mg, 0.018 mmol) and sodium ascorbate (2 mg, 0.009 mmol). The solution was heated at 60°C and stirred for 2 d. After this time, H_2_O (20 mL) and CH_2_Cl_2_ (20 mL) were added. The organic layer was extracted and dried over anhydrous MgSO_4_. After removal of the solvent, the crude product was purified using column chromatography (neutral Brockmann II alumina as stationary phase) using CH_2_Cl_2_ as eluent, to give the target compound as a yellow solid (42 mg, 44%). *R_f_* = 0.49 (CH_2_Cl_2_). ^1^H NMR (500 MHz, acetone-*d_6_*) δ 9.66 (2H, dd, *J* = 5.1, 1.4 Hz), 8.97 (2H, dd, *J* = 8.3, 1.4 Hz), 8.40 (1H, s), 8.31 (2H, s), 8.18 (2H, dd, *J* = 8.3, 5.2 Hz), 7.77 (2H, d, *J* = 8.6 Hz), 7.68 (2H, d, *J* = 8.6 Hz), 4.60–4.51 (1H, m), 1.94–1.00 (30H, m), 0.97–0.04 (6H, m), 0.88–0.86 (6H, m), 0.72 (3H, s). ^13^C NMR (125 MHz, acetone-*d_6_*) δ 163.3, 155.6, 150.1, 147.2, 140.3, 133.2, 131.7, 130.5, 129.1, 127.9, 127.0, 126.0, 119.4, 61.0, 57.4, 57.2, 55.1, 46.3, 43.4, 40.9, 40.3, 38.1, 37.0, 36.6, 36.4, 36.2, 32.8, 28.9, 28.7, 24.9, 24.6, 23.1, 22.8, 21.9, 19.1, 12.6, 12.5. HRMS (ESI, *m/z*) for C_51_H_60_N_9_O_3_Re [M + H]^+^ calc. 1034.4449; found 1034.4432.

#### Synthesis of tetraethylene glycol monotosylate **5**^42^

To a stirring solution of TEG (9.0 mL, 52.13 mmol) in CH_2_Cl_2_ (11 mL) at 0°C, was added TsCl (1.011 g, 5.30 mmol) and Et_3_N (1.1 mL, 7.89 mmol). The reaction was slowly warmed to ambient temperature and stirring was maintained for a further 18 h. The mixture was diluted with CH_2_Cl_2_ (10 mL) and washed with H_2_O (3 × 10 mL), brine (10 mL), dried (Na_2_SO_4_), filtered, and concentrated *in vacuo* to give the desired product (1.593 g, 86%) as a colourless oil. ^1^H NMR (500 MHz, CDCl_3_) δ 7.79 (2H, d, *J* = 8.3 Hz), 7.34 (2H, d, *J* = 8.3 Hz), 4.16 (2H, t, *J* = 4.8 Hz), 3.72–3.56 (14H, m), 2.44 (3H, s). ^13^C NMR (125 MHz, CDCl_3_) δ 145.0, 133.1, 130.0, 128.1, 72.6, 70.9, 70.8, 70.6, 70.5, 69.4, 68.8, 61.9, 21.8. HRMS (ESI, *m/z*) for C_15_H_24_O_7_S [M + H]^+^ calc. 349.1316; found 349.1312.

#### Synthesis of 11-azido tetraethylene glycol **6**^42^

A mixture of tosylate **5** (5.983 g, 17.17 mmol), NaN_3_ (3.361 g, 51.70 mmol), and DMF (60 mL) was stirred at 100°C for 4 h, protected from light. The mixture was concentrated under a stream of N_2_ and the white solid was dissolved in CH_2_Cl_2_ (80 mL). The organic phase was washed with H_2_O (3 × 80 mL) and the aqueous portions were combined and extracted with CH_2_Cl_2_ (3 × 80 mL). The combined organic portions were dried (Na_2_SO_4_), filtered and concentrated *in vacuo* to give the title compound as a pale-yellow oil (2.949 g, 78%). ^1^H NMR (500 MHz, CDCl_3_) δ 3.73–3.72 (2H, m), 3.68–3.61 (10H, m), 3.62–3.60 (2H, m), 3.40–3.38 (2H, m), 2.29 (1H, s). ^13^C NMR (125 MHz, CDCl_3_) δ 72.6, 70.9, 70.8, 70.7, 70.5, 70.2, 61.9, 50.8. HRMS (ESI, *m/z*) for C_8_H_17_N_3_O_4_ [M + H]^+^ calc. 220.1292; found 220.1294.

#### Synthesis of 11-azido-1-mesyltetraethylene glycol **7**^42^

To a stirred solution of alcohol **6** (1.373 g, 6.26 mmol) and Et_3_N (2.5 mL, 17.94 mmol) in anhydrous CH_2_Cl_2_ (35 mL) at 0°C under an inert atmosphere, was added dropwise MsCl (1.00 mL, 12.92 mmol). The reaction was warmed to ambient temperature and stirred for 16 h before being washed with sat. NaHCO_3_ (30 mL), H_2_O (3 × 30 mL), brine (30 mL), dried (Na_2_SO_4_), filtered, and concentrated *in vacuo* to give a yellow oil. The crude material was purified using column chromatography (EtOAc) to give the desired product as a pale-yellow oil (1.427 g, 77%). *R_f_* = 0.46 (EtOAc). ^1^H NMR (500 MHz, CDCl_3_) δ 4.40–4.37 (2H, m), 3.78–3.76 (2H, m), 3.68–3.65 (10H, m), 3.39 (2H, t, *J* = 5.2 Hz), 3.07 (3H, s). ^13^C NMR (125 MHz, CDCl_3_) δ 70.84, 70.80, 70.76, 70.2, 69.4, 69.2, 50.8, 37.8. HRMS (ESI, *m/z*) for C_9_H_19_N_3_O_6_S [M + Na]^+^ calc. 320.0887; found 320.0896.

#### Synthesis of 11-azido-1-phthalimidotetraethylene glycol **8**^42^

A mixture of mesylate **7** (1.420 g, 4.78 mmol), potassium phthalimide (980 mg, 5.29 mmol), and anhydrous DMF (25 mL) was stirred under an inert atmosphere at 120°C, protected from light, for 22 h. The mixture was concentrated under a steam of N_2_ and the off-white solid was suspended in CH_2_Cl_2_ (30 mL) and extracted with H_2_O (30 mL). The aqueous layer was extracted with CH_2_Cl_2_ (3 × 30 mL) and the organic portions were combined and washed with H_2_O (3 × 30 mL), brine (30 mL), dried (Na_2_SO_4_), filtered and concentrated *in vacuo* to give a dark yellow oil. The crude material was purified using column chromatography (20–50% EtOAc in pet. spirits) to give the desired product (1.162 g, 70%) as a colourless oil. *R_f_* = 0.37 (50% EtOAc in pet. spirits). ^1^H NMR (500 MHz, CDCl_3_) δ 7.85–7.83 (2H, m), 7.72–7.70 (2H, m), 3.90 (2H, t, *J* = 5.9 Hz), 3.74 (2H, t, *J* = 5.9 Hz), 3.67–3.59 (10H, m), 3.36 (2H, t, *J* = 5.2 Hz). ^13^C NMR (125 MHz, CDCl_3_) δ 168.4, 134.1, 132.3, 123.4, 70.81, 70.78, 70.3, 70.1, 68.1, 50.8, 37.4. HRMS (ESI, *m/z*) for C_16_H_20_N_4_O_5_ [M + H]^+^ calc. 349.1506; found 349.1486. Anal. RP-HPLC: *t*_R_ 11.87 min, purity > 99%.

#### Synthesis of 1-amino-11-azidoetraethylene glycol **9**^42^

To a solution of thalimide **8** (978 mg, 2.81 mmol) in anhydrous EtOH (28 mL) was added N_2_H_4_·H_2_O (550 μL, 11.34 mmol) and the heterogenous mixture was stirred under an inert atmosphere at 79°C, protected from light for 16 h. The reaction mixture was concentrated under reduced pressure and the white solid was suspended in a solution of 2 M HCl (18 mL) and stirred at 100°C for 1 h. The reaction mixture was cooled to ambient temperature and then cooled on ice for 1 h before the white precipitate was removed using vacuum filtration. The filtrate was neutralised using 3 M NaOH and then concentrated to approximately one third of its volume under a stream of N_2_. The filtrate was diluted with CH_2_Cl_2_ (70 mL) and 4 M NaOH (20 mL) and the two phases were separated. The aqueous portion was extracted with CH_2_Cl_2_ (3 × 70 mL) and the combined organic portions were washed with 3 M NaOH (2 × 30 mL), dried (Na_2_SO_4_), filtered and concentred *in vacuo* to give the title compound as a colourless oil (586 mg, 96%). ^1^H NMR (500 MHz, CDCl_3_) δ 3.68–3.61 (10H, m), 3.52 (2H, t, *J* = 5.2 Hz), 3.38 (2H, t, *J* = 5.2 Hz), 2.87 (2H, t, *J* = 5.3 Hz), 1.83 (2H, br s). ^13^C NMR (125 MHz, CDCl_3_) δ 73.3, 70.80, 70.78, 70.76, 70.4, 70.2, 50.8, 41.8. HRMS (ESI, *m/z*) for C_8_H_18_N_4_O_3_ [M + H]^+^ calc. 219.1452; found 219.1455.

#### Synthesis of cholestenyl-TEG-azide **10**

A solution of cholesterol (46 mg, 0.119 mmol), CDI (25 mg, 0.155 mmol), and anhydrous THF (400 μL) was stirred at ambient temperature, under an inert atmosphere for 24 h. To the stirring solution was added amine **9** (157 mg, 0.719 mmol) and the reaction was stirred at ambient temperature for 24 h. The reaction mixture was concentrated under a stream of N_2_ and the resulting oil was dissolved in EtOAc (10 mL) and washed with H_2_O (2 × 10 mL), brine (10 mL), dried (Na_2_SO_4_), filtered and concentrated under reduced pressure. The crude material was purified using column chromatography (20–50% EtOAc in pet. spirits) to give the desired product (38 mg, 51%) as a colourless, viscous oil. *R_f_* = 0.41 (50% EtOAc in pet. spirits). ^1^H NMR (500 MHz, CDCl_3_) δ 5.37–5.36 (1H, m), 5.14 (1H, br s), 4.51–4.47 (1H, m), 3.69–3.61 (10H, m), 3.56–3.54 (2H, m), 3.40–3.35 (4H, m), 2.37–2.34 (1H, m), 2.29–2.24 (1H, m), 2.01–1.79 (5H, m), 1.72–0.85 (33H, m), 0.67 (3H, s). ^13^C NMR (125 MHz, CDCl_3_) δ 156.3, 140.0, 122.6, 74.4, 70.80, 70.79, 70.76, 70.4, 70.3, 70.2, 56.8, 56.3, 50.8, 50.2, 42.5, 40.8, 39.9, 39.7, 38.7, 37.1, 36.7, 36.3, 35.9, 32.04, 32.01, 28.4, 28.3, 28.2, 24.5, 24.0, 23.0, 22.7, 21.2, 19.5, 18.9, 12.0. HRMS (ESI, *m/z*) for C_36_H_62_N_4_O_5_ [M + H]^+^ calc. 631.4793; found 631.4795.

#### Synthesis of cholestanyl-TEG-azide **11**

A solution of 5α-cholestan-3β-ol (85 mg, 0.219 mmol), CDI (49 mg, 0.302 mmol), and anhydrous THF (800 μL) was stirred at ambient temperature, under an inert atmosphere for 24 h. To the stirring solution was added amine **9** (289 mg, 1.32 mmol) and the reaction was stirred at ambient temperature for 24 h. The reaction mixture was concentrated under a stream of N_2_ and the resulting oil was dissolved in EtOAc (10 mL) and washed with H_2_O (2 × 10 mL), brine (10 mL), dried (Na_2_SO_4_), filtered and concentrated under reduced pressure. The crude material was purified using column chromatography (50% EtOAc in pet. spirits) to give the desired product (99 mg, 71%) as a colourless, viscous oil. *R_f_* = 0.37 (50% EtOAc in pet. spirits). ^1^H NMR (500 MHz, CDCl_3_) δ 5.12–5.06 (1H, m), 4.60–4.55 (1H, m), 3.69–3.61 (10H, m), 3.56–3.54 (2H, m), 3.40–3.35 (4H, m), 1.97–1.93 (1H, m), 1.84–1.76 (2H, m), 1.73–1.69 (1H, m), 1.66–0.85 (35H, m), 0.81 (3H, s), 0.64 (3H, s). ^13^C NMR (125 MHz, CDCl_3_) δ 156.5, 74.2, 70.9, 70.80, 70.76, 70.4, 70.3, 70.2, 56.6, 56.4, 54.4, 50.8, 44.8, 42.7, 40.8, 40.1, 39.7, 36.9, 36.3, 35.9, 35.63, 35.57, 34.6, 32.1, 28.8, 28.4, 28.2, 28.0, 24.3, 24.0, 23.0, 22.7, 21.3, 18.8, 12.4, 12.2. HRMS (ESI, *m/z*) for C_36_H_64_N_4_O_5_ [M + H]^+^ calc. 633.4949; found 633.4965.

#### Synthesis of **ReTEGCholesterol**

A mixture of **ReAlkyne** (31 mg, 0.050 mmol), azide **10** (37 mg, 0.059 mmol), sodium ascorbate (11 mg, 0.056 mmol), and CuSO_4_·5H_2_O (6 mg, 0.024 mmol) in 1:1 CH_2_Cl_2_/H_2_O (2 mL) was stirred at ambient temperature under an inert atmosphere for 24 h. The reaction mixture was diluted with CH_2_Cl_2_ (5 mL) and transferred to a separatory funnel. The organic phase was washed with H_2_O (3 × 5 mL), brine (5 mL), dried (Na_2_SO_4_), filtered and concentrated *in vacuo* to give a yellow film. The crude material was purified using column chromatography (CH_2_Cl_2_ to 2% MeOH in CH_2_Cl_2_ to 10% MeOH in CH_2_Cl_2_) to give the title compound (27 mg, 44%) as a waxy, yellow solid. *R_f_* = 0.61 (10% MeOH in CH_2_Cl_2_). m.p. 117–119°C. ^1^H NMR (500 MHz, CDCl_3_) δ 9.53 (2H, d, *J* = 5.0 Hz), 8.58 (2H, d, *J* = 8.0 Hz), 8.01 (2H, s), 7.96 (1H, s), 7.91–7.90 (4H, m), 7.75 (2H, d, *J* = 8.0 Hz), 5.35 (1H, d, *J* = 4.6 Hz), 5.14 (1H, t, *J* = 4.7 Hz), 4.57 (2H, t, *J* = 4.6 Hz), 4.49–4.43 (1H, m), 3.89 (2H, t, *J* = 4.9 Hz), 3.61–3.46 (10H, m), 3.30–3.29 (2H, m), 2.35–2.21 (2H, m), 2.01–1.81 (6H, m), 1.57–0.85 (32H, m), 0.67 (3H, s). ^13^C NMR (125 MHz, CDCl_3_) δ 196.4, 193.4, 162.1, 156.3, 153.9, 153.1, 147.6, 147.4, 140.0, 138.8, 138.2, 131.3, 130.6, 127.8, 127.2, 126.2, 125.8, 122.6, 121.3, 74.4, 70.7, 70.60, 70.56, 70.3, 70.2, 69.6, 56.8, 56.2, 50.4, 50.1, 42.4, 40.7, 39.9, 39.6, 38.7, 37.1, 36.7, 36.3, 35.9, 32.02, 31.98, 28.4, 28.3, 28.1, 24.4, 24.0, 23.0, 22.7, 21.2, 19.5, 18.8, 12.0. HRMS (ESI, *m/z*) for C_60_H_75_N_10_O_8_Re [M + H]^+^ calc. 1251.5400; found 1251.5424. Anal. RP-HPLC: *t*_R_ 25.48 min, purity > 96%.

#### Synthesis of **ReTEGCholestanol**

A mixture of **ReAlkyne** (35 mg, 0.056 mmol), azide **11** (44 mg, 0.070 mmol), sodium ascorbate (26 mg, 0.131 mmol), and CuSO_4_·5H_2_O (14 mg, 0.056 mmol) in 1:1 CH_2_Cl_2_/H_2_O (2.4 mL) was stirred at ambient temperature under an inert atmosphere for 24 h. The reaction mixture was diluted with CH_2_Cl_2_ (15 mL) and transferred to a separatory funnel. The organic phase was washed with H_2_O (3 × 10 mL), brine (10 mL), dried (Na_2_SO_4_), filtered and concentrated *in vacuo* to give a yellow film. The crude material was purified using column chromatography (CH_2_Cl_2_ to 3% MeOH in CH_2_Cl_2_) to give the title compound (32 mg, 46%) as a yellow wax. *R_f_* = 0.51 (10% MeOH in CH_2_Cl_2_). ^1^H NMR (500 MHz, CDCl_3_) δ 9.54 (2H, d, *J* = 5.1 Hz), 8.58 (2H, d, *J* = 8.2 Hz), 8.01 (2H, s), 7.95–7.88 (5H, m), 7.74 (2H, d, *J* = 8.2 Hz), 5.10 (1H, br s), 4.58–4.56 (2H, m), 3.89 (2H, t, *J* = 4.9 Hz), 3.62–3.54 (8H, m), 3.47 (2H, t, *J* = 4.9 Hz), 3.31–3.28 (2H, m), 1.96–1.93 (1H, m), 1.82–1.78 (2H, m), 1.70–0.78 (41H, m), 0.64 (3H, s). ^13^C NMR (125 MHz, CDCl_3_) δ 196.4, 193.5, 156.5.1, 153.9, 153.2, 147.6, 138.7, 138.2, 130.6, 127.8, 127.2, 126.2, 126.0, 125.8, 121.3, 74.2, 70.7, 70.61, 70.58, 70.3, 70.2, 69.6, 56.5, 56.4, 50.3, 50.5, 44.8, 42.7, 40.8, 40.1, 39.6, 36.9, 36.3, 35.9, 35.60, 35.55, 34.6, 32.1, 28.8, 28.4, 28.1, 28.0, 24.3, 24.0, 23.0, 22.7, 21.3, 18.8, 12.4, 12.2. HRMS (ESI, *m/z*) for C_60_H_77_N_10_O_8_Re [M + H]^+^ calc. 1253.5556; found 1253.5543. Anal. RP-HPLC: *t*_R_ 23.61 min, purity > 93%.

### General methods for cell application

Human prostate non-malignant PNT1a and prostate cancer 22Rv1, LNCaP (clone FCG), and PC3 cell lines were obtained from the European Collection of Cell Cultures via CellBank Australia (Children's Medical Research Institute, Westmead, NSW, Australia). Prostate cell lines PNT1a, 22Rv1, and LNCaP were maintained with RPMI culture medium (Gibco) supplemented with 10% fetal bovine serum (Moregate BioTech). PC3 cell lines were maintained in Ham's F12K culture medium (Gibco) supplemented with 10% fetal bovine serum. Culture media were replaced every 2 to 3 d and cells were sub-cultured when they reached 80–90% confluency. Cells were maintained at 37°C with 5% CO_2_. For experiments cells were cultured at the following densities: PNT1a 8 × 10^4^; 22Rv1 8 × 10^4^; LNCaP 6 × 10^4^; and PC3 4 × 10^4^. All cell lines were maintained in RPMI culture medium supplemented with 10% fetal bovine serum for 24 h, once seeded for experiments. Following 24 h, the culture medium was replaced with fresh RPMI culture medium containing no fetal bovine serum and cells were incubated for a further 24 h. Serum starvation was applied as serum contains albumin which interacts with neutral Re(I) complexes, affecting their emission properties^43^ and which may enhance cholesterol efflux from cells.^44,45^ It also contains cholesterol which would likely interfere with **ReTEGCholestanol** uptake. Once cells were starved, they were treated with an imaging compound by replacing half of the culture medium with fresh warm culture medium containing the imaging compound of interest at 10 μM to give a final concentration of ∼5 μM. For the final staining protocols, the 10 μM solution of **ReTEGCholestanol** was centrifuged (Eppendorf, 5424 R) at 15000 rpm for 10 min to remove any solids/aggregates. Only small quantities of particulate matter were removed following centrifugation of **ReTEGCholesanol** and therefore, while we acknowledge the final concentration used for incubation was less than the ideal 5 μM, it is not anticipated to be significantly less. Control cells were treated with the equivalent amount of DMSO. Cells were incubated for a further 24 h prior to imaging or for other staining applications.

### Cell Discoverer 7 imaging and quantification of luminescence intensity

For Cell Discoverer 7 (Zeiss) high throughput widefield microscope imaging, cells were seeded on glass-bottom 96-well plates (Greiner) and stained with **ReTEGCholestanol** as described above (Section 2.3). Cells were imaged under culture conditions (37°C, 5% CO_2_) using 50x Plan-Apochromat Objective, with 385 nm excitation wavelength (LED light intensity 5.4%, exposure time 70 ms) and phase gradient channel (transmitted light intensity 10%, exposure time 48 ms). Five images were taken per cell line.

The luminescence intensity of **ReTEGCholestanol** for each image was quantify using Zen Blue software (3.0), according to the equation:

(1) \begin{equation*}{{\mathrm{L}}}_{{\mathrm{intensity}}} = {{\mathrm{L}}}_{{\mathrm{ReTEGCholestanol}}} - {{\mathrm{L}}}_{{\mathrm{DMSO}}}\end{equation*}

where L is luminescence. The luminescence intensity for each cell line was an average of five images taken per experiment, which was repeated 3 times independently. The data were presented as mean ± standard deviation (SD).

### Cell viability assay

Cell viability was assessed using the colorimetric MTS assay (Promega). Cells were seeded onto black 96-well plates (Corning Costar) and incubated with the compound of interest or DMSO as described in Section 2.3. Untreated cells kept for 24 h in RPMI media with no fetal bovine serum served as a control for this assay. After a 24 h incubation, MTS reagent was added at 10% of the total volume and cells were then incubated for a further 3 h. The absorbance was measured at 490 nm wavelength using an EnSpire Plate Reader (Perkin Elmer). Cell viability was reported as a proportion, calculated by comparing the absorbance of cells treated with the compound of interest, or DMSO against the control cells, which were assigned a viability value of 1. Final values have been reported as the mean ± SD of four biological replicates.

### Cell staining and immunofluorescence

For co-staining studies, cells were seeded on 8-well μ-slides (Ibidi). Following 24 h incubations with **ReTEGCholestanol** further staining was performed using specific dyes for acidic vesicles (LysoTracker^TM^; Invitrogen), mitochondria (MitoTracker^TM^; Invitrogen), neutral lipids/lipid droplets (LipidTox^TM^; Invitrogen) or plasma membrane (CellMask^TM^, Invitrogen). Live cells were stained with 75 nM Lysotracker^TM^ Deep Red for 40 min or with 0.5 μM MitoTracker^TM^ Deep Red for 20 min in serum-free media under culture conditions. After incubation with a specific probe, the staining solution was removed, and fresh culture medium was added to the cells followed by confocal imaging. For neutral lipids/lipid droplets staining, cells were fixed for 12 min with cold solution containing 8% paraformaldehyde and 8% sucrose in PBS (fixative solution), which was added to cells in warm culture medium at 1:1 ratio. After fixation, cells were washed 3 × 5 min in PBS, followed by LipidTox^TM^ Deep Red staining (1:1000 in PBS, 30 min), following a wash step confocal imaging was performed. For cell membrane staining live cells were incubated with CellMask (1:1000) for 5 min in serum-free culture medium under culture conditions and then washed with PBS. Cells were then fixed with 4% paraformaldehyde, washed 3 times with PBS and imaged in PBS using confocal microscopy.

For immunofluorescence, cells were cultured on glass coverslips in 24-well plate (Falcon) and then incubated for 24 h with **ReTEGCholestanol** and fixed with cold fixative solution as described above. Cells were blocked and permeabilized in blocking buffer (2.5% BSA containing 0.05% saponin) for 1 h before incubation with primary antibodies (diluted in blocking buffer) at 4°C overnight. Cells were washed 3 × 5 min in PBS, followed by secondary antibody (diluted in blocking buffer) staining (anti-mouse or anti-rabbit Alexa Fluor 647; Invitrogen) performed for 1 h at room temperature. Cells were then washed 3 × 5 min in PBS and mounted in Prolong Gold media containing DAPI (Thermofisher). The slides were allowed to cure for 24–48 h before imaging. Immunofluorescence was performed using antibodies against Rab5 (Abcam ab18211, 5 μg/mL), EEA1 (Thermofisher, SC-6415, 1 μg/mL), CD63 (Abcam ab1318, 2 μg/mL), and Lamp1 (Abcam ab24170, 5 μg/mL).

### Confocal Imaging

Cells were imaged with a Nikon A + confocal microscope (Nikon, Japan), equipped with a LU-N4/LU-N4S 4-laser unit (403, 488, 561, and 638 nm), the A1-DUG GaAsP Multi Detector Unit (2 GaAsP PMTs + 2 standard PMTs), a 32 channel spectral detector (Nikon, Minato, Tokyo, Japan) and a Uno‐Combined‐Controller, CO_2_ microscope electric top stage incubation system (Okolab, Pozzuoli, NA, Italy) to maintain live cells at 37°C and 5% CO_2_.^46^  **ReTEGCholestanol** signal was recorded using 403 nm excitation wavelength (2% laser power) and 505–600 nm detection, while deep red signal was recorded using 638 nm excitation wavelength (5% laser power) and 665–750 nm detection. Single-stained controls were imaged with each luminescence channel to identify and minimize bleed-through. Images were captured using a Plan Apo λ 60x oil immersion objective.

### Co-localization measurement

Thresholded Mander's co-localisation coefficients (M1, M2) were obtained using ImageJ software (2.1.0) with JACoP plug-in. Mander's coefficients report the proportion of signal in one channel that co-localizes with the other channel.^47^ In our case M1 coefficient informs on the amount of **ReTEGCholestanol** which overlaps with the other fluorophore, while M2 indicates a given fluorophore signal that overlaps with **ReTEGCholestanol**. Five images were analyzed for each marker and the data were presented as mean ± SD.

## Results

### Chemical synthesis and characterization

**Scheme 1:**
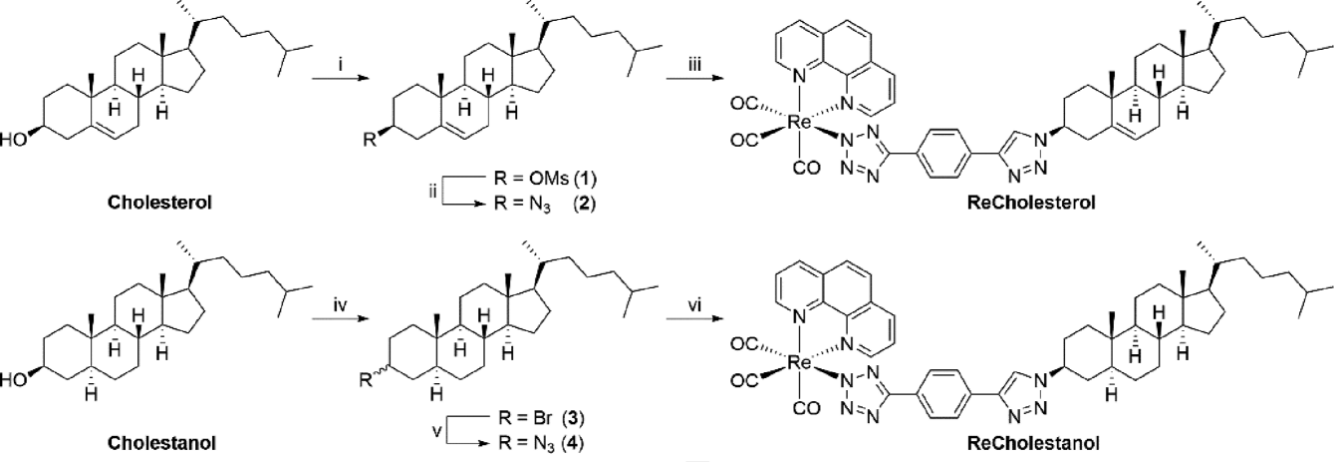
Reagents and conditions: (i) MsCl, Et_3_N, CH_2_Cl_2_, 21°C, 4 h, **1** (86%); (ii) TMSN_3_, BF_3_·(OEt)_2_, CH_2_Cl_2_, 21°C, 16 h, **2** (60%); (iii) **ReAlkyne**, CuSO_4_·5H_2_O, sodium ascorbate, THF/H_2_O 3:1, 50°C, 2 d, **ReCholesterol** (55%); (iv) CBr_4_, PPh_3_, CH_2_Cl_2_, 21°C, 16 h, **3** (99%); (v) NaN_3_, DMF, 100°C, 16 h, **2** (62%); (vi) **ReAlkyne**, CuSO_4_·5H_2_O, sodium ascorbate, THF/H_2_O 3:1, 50°C, 2 d, **ReCholestanol** (44%).

A copper(I)-catalyzed alkyne azide cycloaddition (CuAAC) approach—a common form of “click chemistry”—was used for the synthesis of our desired Re(I) steroidal conjugates, specifically cholesterol and cholestanol. In all instances **Realkyne** was employed as the alkyne coupling partner, whilst the azide components were prepared in-house. Functionalization through the steroidal hydroxyl handle was selected as this has previously been performed for the preparation of cholesterol^40^ and cholestanol^48^ conjugates. Direct conjugations between each of the two steroidal frameworks and **Realkyne** was pursued first (Scheme 1). In order to preserve stereochemistry at the C3-position, the hydroxyl group of cholesterol was firstly activated using methanesulfonyl chloride (MsCl), followed by substitution with the requisite azido moiety using trimethylsilyl azide (TMSN_3_) and a Lewis acid to give **2**.^49^ Conjugation of **2** with **Realkyne** proceeded smoothly to give **ReCholesterol** in 55% yield after a chromatographic purification step. When the same strategy was trialled on cholestanol the azidation step failed with no consumption of starting material observed, as evidenced by ^1^H NMR analysis. Therefore, cholestanol azide **4** was accessed over two S_N_2 steps; bromination and azidation using NaN_3_.^41^ CuAAC coupling between **Realkyne** and azide **4** was then performed to give **ReCholestanol** in 44%.

**Scheme 2:**
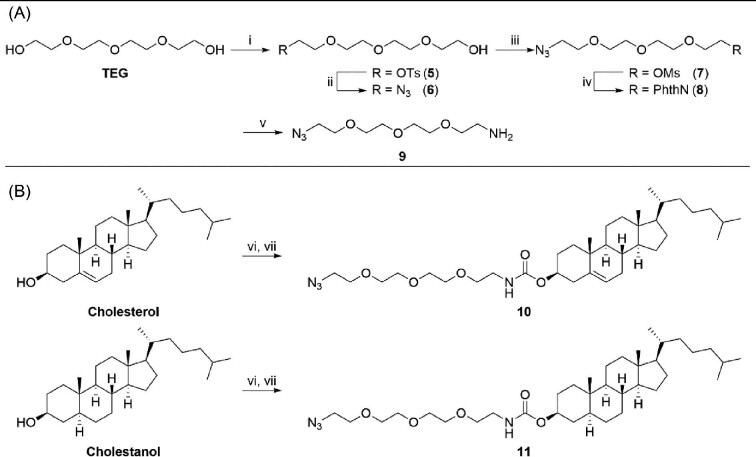
Synthesis of linker **9** (**A**) and azido steroids **10** and **11** (**B**). Reagents and conditions: (i) TsCl, Et_3_N, CH_2_Cl_2_, 21°C, 16 h, **5** (86%); (ii) NaN_3_, DMF, 100°C, 4 h, **6** (78%); (iii) MsCl, Et_3_N, CH_2_Cl_2_, 21°C, 16 h, **7** (77%); (iv) Potassium thalimide, DMF, 120°C, 22 h, **8** (70%); (v) N_2_H_4_·H_2_O, EtOH, 79°C, 16 h, **9** (96%); (vi) CDI, THF, 21°C, 24 h; (vii) **9**, THF, 21°C, 16 h, **10** (51%), and **11** (71%).

We also prepared two Re(I) complexes in which the steroid moiety (cholesterol or cholestanol) was separated from the Re(I) moiety by a short TEG linker to improve the water solubility and limit interference of the Re(I) complex with potential cholesterol-membrane interactions. Following the procedure described by Trinh and co-workers,^42^ azido-TEG-NH_2_  **9** was synthesised over five steps from TEG using a protecting group strategy. Briefly, TEG was selectively protected using *p*-toluenesulfonyl chloride (TsCl) under basic conditions to give **5** which was then azidated using NaN_3_ in DMF at 100°C. The remaining alcohol handle was then converted to mesylate **7** before being substituted with potassium thalimide under thermal conditions to give **8**. Reduction of thalimide **8** using hydrazine monohydrate (N_2_H_4_·H_2_O) proceeded smoothly to give the requisite azido-TEG-NH_2_ linking group. Installation of amine **9** onto cholesterol has been previously reported^50^ and as such, cholesterol and cholestanol were each functionalized over two steps in one-pot using 1,1′-carbonyldiimidazole (CDI) in anhydrous tetrahydrofuran (THF) to firstly form the reactive ester intermediate, before amine **9** was added to ultimately form a carbamate. The desired carbamates (**10** and **11**, Scheme 2) were isolated after a chromatographic purification step in each case.

**Scheme 3:**
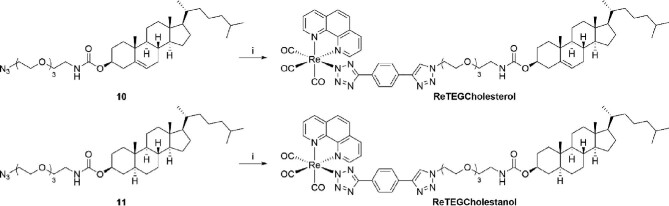
Reagents and conditions: (i) **ReAlkyne**, CuSO_4_·5H_2_O, sodium ascorbate, CH_2_Cl_2_/H_2_O, 21°C, 24 h, **ReTEGCholesterol** (44%), and **ReTEGCholestanol** (46%).

With azides **10** and **11** in hand, CuAAC coupling was carried out using **ReAlkyne** in a biphasic mixture of CH_2_Cl_2_ in H_2_O (Scheme 3). Each conjugate required a careful chromatographic purification step in order to isolate the desired products. Attempts to further improve the purity of both conjugates by crystallization and preparative HPLC were unsuccessful, however, ^1^H NMR analysis indicated the impurity was unreacted **10** or **11**, respectively. Given the aim of this study is to track cholesterol derivatives in live cells, we decided a small amount of this impurity would unlikely interfere in our pursuit. Therefore, the four Re(I)steroidal conjugates were of acceptable purity to proceed with biological evaluation.

### Biological compatibility

In order to deliver the cholesterol compounds into live cells or other biological systems, most protocols require the incubation of cells with the compound at low concentration in aqueous solutions, such as cell culture media or physiological buffers.^51,52^ To overcome the non-polar nature of the compounds synthesized in this study, serial dilution steps were performed, first into DMSO, which can dissolve both polar and non-polar materials, followed by dilution into the aqueous media for final delivery. Unfortunately, ReCholesterol and ReCholestanol were unable to be properly dissolved in aqueous cell culture media using an acceptable concentration of DMSO for live cell imaging (i.e., <1%);^53^ these complexes were therefore excluded from further cell biology imaging experiments. The introduction of the TEG linker did improve compound solubility and complexes were able to be solubilized to a level that allowed incubations with live cells. Imaging by confocal microscopy revealed precipitation of the complexes, especially ReTEGCholesterol, on the cell membranes and surface of the slides, which was unable to be removed, even with vigorous wash steps. The particulate matter was not expected to be the effect of the compounds decomposition as our Re(I) tricarbonyl tetrazolato complexes are highly stable^36^ and the fluorescent crystals were visible straight after adding the probes to cells. To address this issue culture media containing ReTEGCholesterol or ReTEGCholestanol were centrifuged (Eppendorf, 5424 R at 15000 rpm for 10 min) to remove any insoluble or aggregate material. Following this step, only ReTEGCholestanol was still able to be detected in cells by fluorescence microscopy and was therefore selected for further evaluation by cellular imaging.

**Fig. 1:**
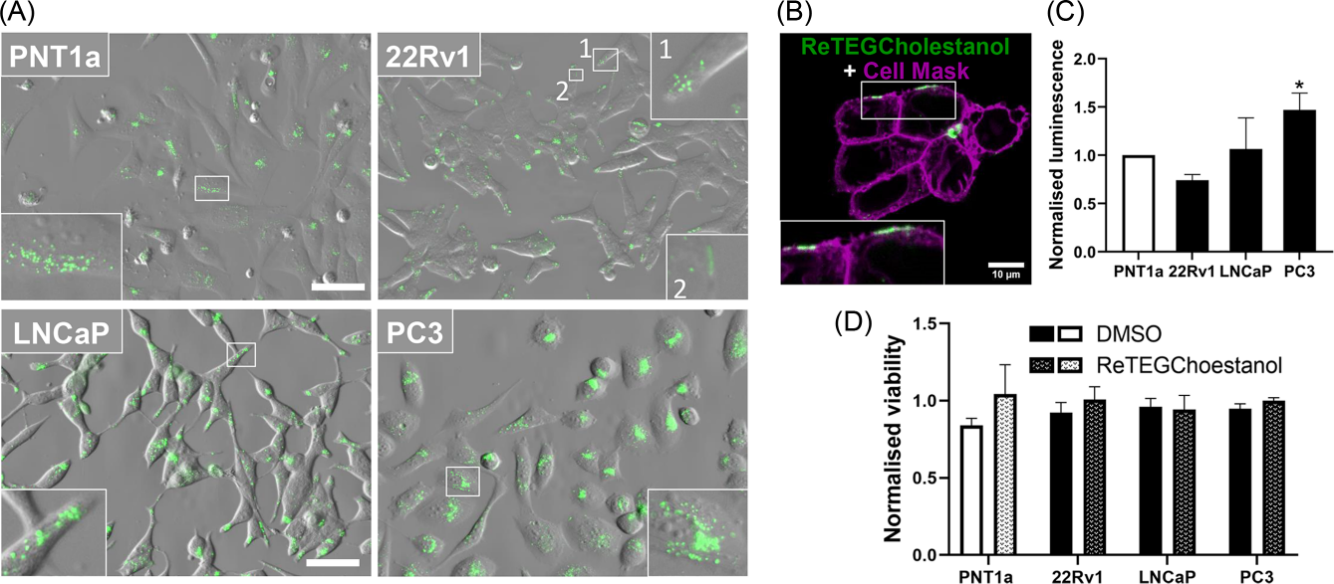
ReTEGCholestanol accumulates in prostate cell lines and does not affect cell viability. (A) Images of live, serum-starved prostate cells incubated for 24 h with ∼5 μM ReTEGCholestanol compound. Insets show that ReTEGCholestanol localizes to punctate vesicular structures in the cytoplasm of non-malignant and malignant prostate cell lines, and in 22Rv1 cell lines was detected in compartments in close proximity or at the cell surface (22Rv1, inset 2). Scale bar in A is 50 μm. (B) Confocal image showing co-localization between ReTEGCholestanol and the plasma membrane marker—CellMask in 22Rv1 cells. Scale bar in B is 10 μm. (C) Luminescence intensity of ReTEGCholestanol in tested cell lines. Data are presented as mean ± SD of three independent experiments. * *P* < 0.05 significant difference between PNT1a and PC3 cells by one-way ANOVA and Dunnett's multiple comparisons test. (D) Viability of cells after 24 h incubation with ReTEGCholestanol or vehicle (DMSO) normalized to untreated cells, which were assigned a viability value of 1. Data are presented as mean ± SD of four biological repeats.

The imaging of ReTEGCholestanol was conducted in four prostate cell lines: non-malignant cell line PNT1a and malignant cell lines 22Rv1, LNCaP and PC3. We chose prostate cancer cells for this study as alterations in lipid metabolism, including cholesterol, are a hallmark of prostate cancer. The malignant cell lines differ in their origin, androgen-sensitivity and consequently lipid-dependance. LNCaP cell line was derived from lymph node metastatic site and is androgen-dependent. 22Rv1 cell line comes from a xenograft, is considered slightly androgen-sensitive and expresses constitutively active variant of androgen receptor. PC3 cell line was derived from bone metastasis, is androgen-insensitive and represents aggressive phenotype of prostate cancer.^54,55^ As a result, all these cell lines represent different biology, lipid profiles and different alterations of lipid metabolism.^46,56,57^ Thus, we assumed that these model cell lines would provide an opportunity to track different cellular pathways/fates of cholesterol. Live cells were incubated with ∼5 μM ReTEGCholestanol for 24 h and then imaged using widefield automated fluorescence microscopy (Cell Discoverer 7). All four cell lines showed significant internalization of ReTEGCholestanol, which was observed in cytoplasmic vesicles (Fig. 1A), and in 22Rv1 cells there was also occasional plasma membrane co-localization (Fig. 1A, B). The amount of ReTEGCholestanol uptake varied between cell lines, which may reflect variations in lipid sequestering and or metabolism (Fig. 1A, C). Luminescence intensity measurements revealed that PC3 cells internalized significantly higher amounts of ReTEGCholestanol than non-malignant PNT1a cells (Fig. 1C).

Cells treated with ∼5 μM ReTEGCholestanol had a normal morphology upon imaging and there were no observable signs of increased cell death in the presence of the compound. Cell viability was quantitatively assessed by MTS assay, which showed no significant difference between any of the cells treated with ReTEGCholestanol and with DMSO vehicle alone (Fig. 1D). ReTEGCholestanol was therefore deemed to be non-toxic and amenable for live cell imaging.

### Biodistribution of ReTEGCholestanol

The biodistribution of ReTEGCholestanol was assessed in the non-malignant epithelial type cell line (PNT1a) and three different malignant prostate cancer cell lines; androgen-responsive 22Rv1 and LNCaP cancer cells and androgen non-responsive, more aggressive, lipid dependent PC3 cancer cells.^58^ Cholesterol can be sequestered/internalized into cells and distributed into different subcellular compartments including lipid droplets,^59^ mitochondria,^60^ and endosomes-lysosomes,^24,61^ we therefore assessed the localization of ReTEGCholestanol in conjunction with specific markers for these important organelles.

**Fig. 2:**
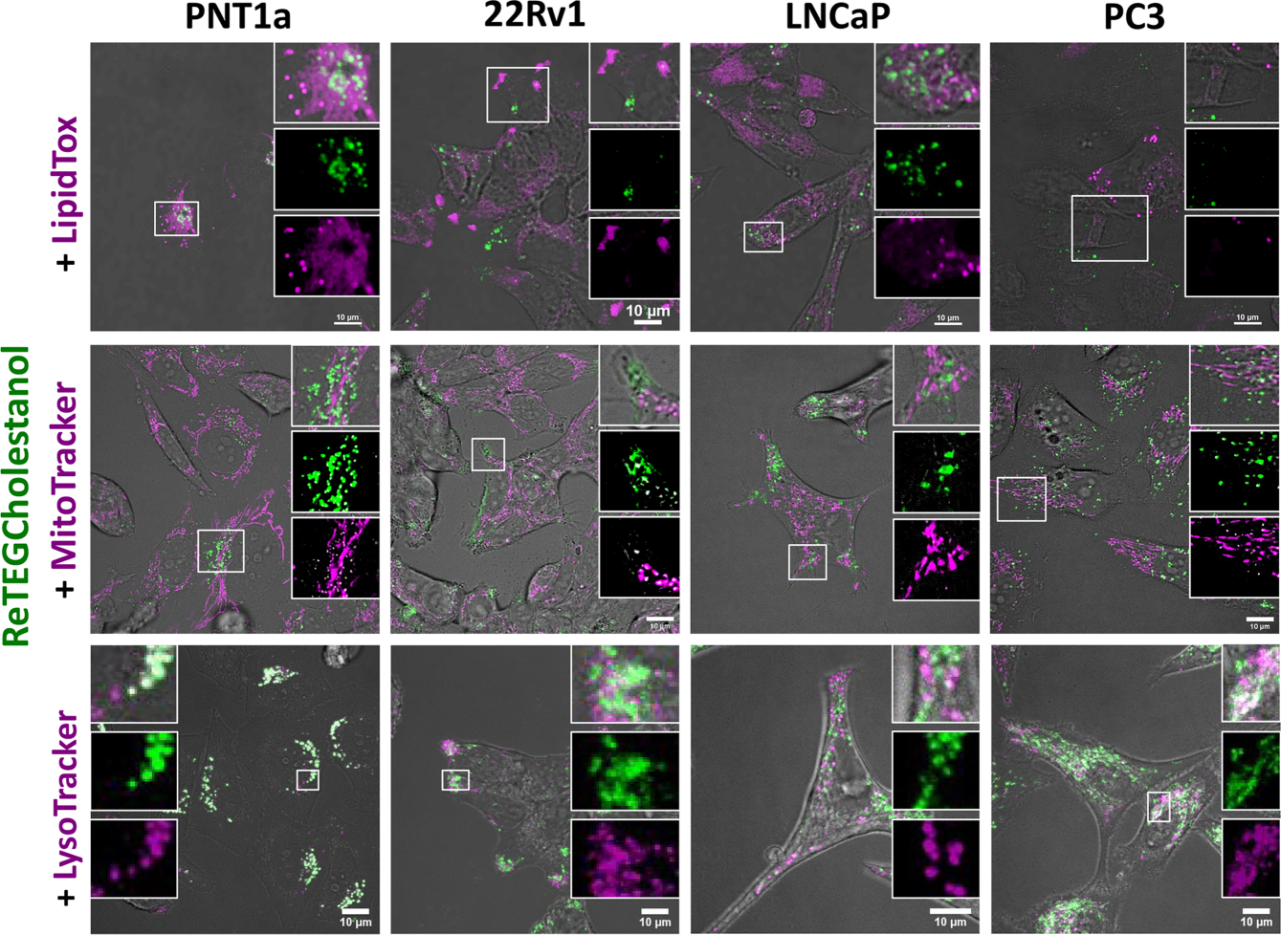
ReTEGCholestanol does not co-localize with lipid droplets (Lipid Tox staining) or mitochondria (MitoTracker stainnig), but accumulates to a variable extent in acidic vesicles (LysoTracker staining) of different prostate cell lines. Serum-starved prostate cells were incubated for 24 h with ∼5 μM ReTEGCholestanol and then stained live with MitoTracker Deep Red or LysoTracker Deep Red, or fixed and stained with LipidTox Deep Red. To avoid cross-talk, dyes emitting in far-red channel (excitation/emission ∼644/665 nm) were selected for imaging. Scale bars; 10 μm.

Lipid droplets are the primary site for lipid storage and were visualized using LipidTox; selected in preference to commonly used BODIPY dyes due to its far-red emission profile, which avoided crosstalk and bleed through with the green emission from ReTEGCholestanol. Since LipidTox stains neutral lipids, it interacts with lipid droplets and to some extent with the endoplasmic reticulum (Fig. 2). Lipid droplet staining differed between the cell lines with PNT1a displaying intensely stained spherical structures with a perinuclear distribution, while 22Rv1 cells displayed a polarized distribution of lipid droplets mainly near the cell periphery, whereas LNCaP and PC3 had more of a dispersed lipid droplet distribution (Fig. 2); and this was consistent with previous reports of lipid droplet distribution in these cell lines.^46^ As the luminescent probe attachment point to cholestanol prevents the esterification of the complex, which is required to access the storage pathway, ReTEGCholestanol did not co-localize with lipid droplets (Fig. 2). There was however some co-location of ReTEGCholestanol-positive compartments with a subset of lipid droplets in PNT1a cells (Fig. 2, PNT1a, inset).

Since cholesterol can be sequestered to mitochondria in cancer cells,^60^ we performed co-staining with MitoTracker and ReTEGCholestanol in prostate cell lines. While the ReTEGCholestanol-positive structures appeared to be in close proximity to some mitochondria, there was little or no co-localization between the complex and MitoTracker dye in any of the cell lines (Fig. 2).

Lysosomes are involved in cholesterol trafficking and a recently developed cholestanol-rhodamine probe was distributed to acidic vesicles in 3T3-L1 mouse adipocytes.^24^ We therefore assessed the co-localization between ReTEGCholestanol and LysoTracker, which mainly detects acidified late endosomes and lysosomes. The ReTEGCholestanol co-localized with LysoTracker positive acidic vesicles in the non-malignant prostate cell line PNT1a (Fig. 2), although there was a proportion of LysoTracker-stained vesicles that did not contain ReTEGCholestanol (Fig. 2). This suggests that ReTEGCholestanol distributes to only a subset of the late endosomes/lysosomes in PNT1a cells. In contrast, ReTEGCholestanol had little co-localization to LysoTracker positive acidic vesicles in 22Rv1 and LNCaP prostate cancer cells, but there was a close association observed between ReTEGCholestanol and LysoTracker-stained vesicles (Fig. 2). In the PC3 prostate cancer cell line there was, however, co-localization between ReTEGCholestanol and LysoTracker, but to a lesser extent than that observed in PNT1a cells (Fig. 2).

The localization of ReTEGCholestanol with late endosomes/lysosomes is consistent with reports of other cholesterol tracking probes,^24,59,61^ suggesting a possible application for this complex for investigating cholesterol trafficking in the endosomal pathway. Distinct staining patterns in all of the of the prostate cell lines suggests differences in cholesterol trafficking between normal and malignant cells, which is in accordance with the well-established idea of altered lipid metabolism in prostate cancer cells.^46,56,57^

**Fig. 3:**
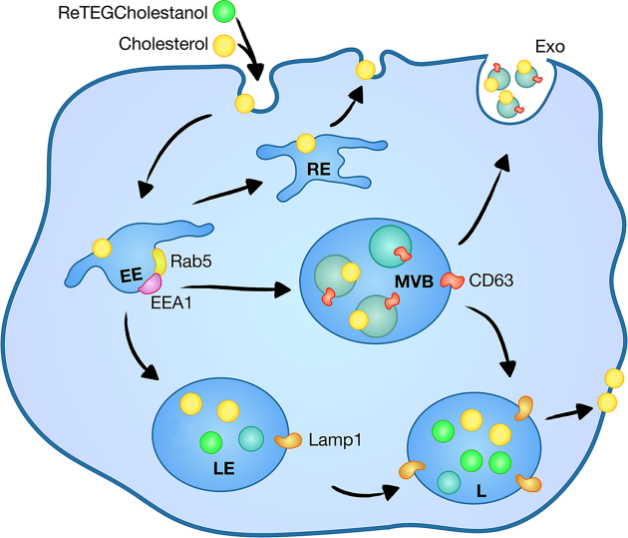
Schematic of cholesterol endosomal pathway and localization of ReTEGCholestanol to late endosomes and lysosomes in non-malignant prostate cells after 24 h incubation. EE, early endosomes; RE, recycling endosomes; LE, late endosomes; L, lysosomes; MVB, multivesicular bodies; Exo, exosomes.

It has previously been demonstrated that the endosomal pathway is altered in prostate cancer cell lines.^62^ Since exogenous cholesterol is incorporated into cells via the endosomal pathway,^61^ we investigated the localization of ReTEGCholestanol in prostate cancer cell lines in relation to endosomal compartments. ReTEGCholestanol treated cells were co-stained with selected markers for the endosomal pathway, to depict early endosomes (Rab5, EEA1), late endosomes/multivesicular bodies (CD63) and late endosomes/lysosomes (Lamp1) (Fig. 3). CD63 is also a marker of exosomes, however, we did not isolate exosomes in this study.

**Fig. 4:**
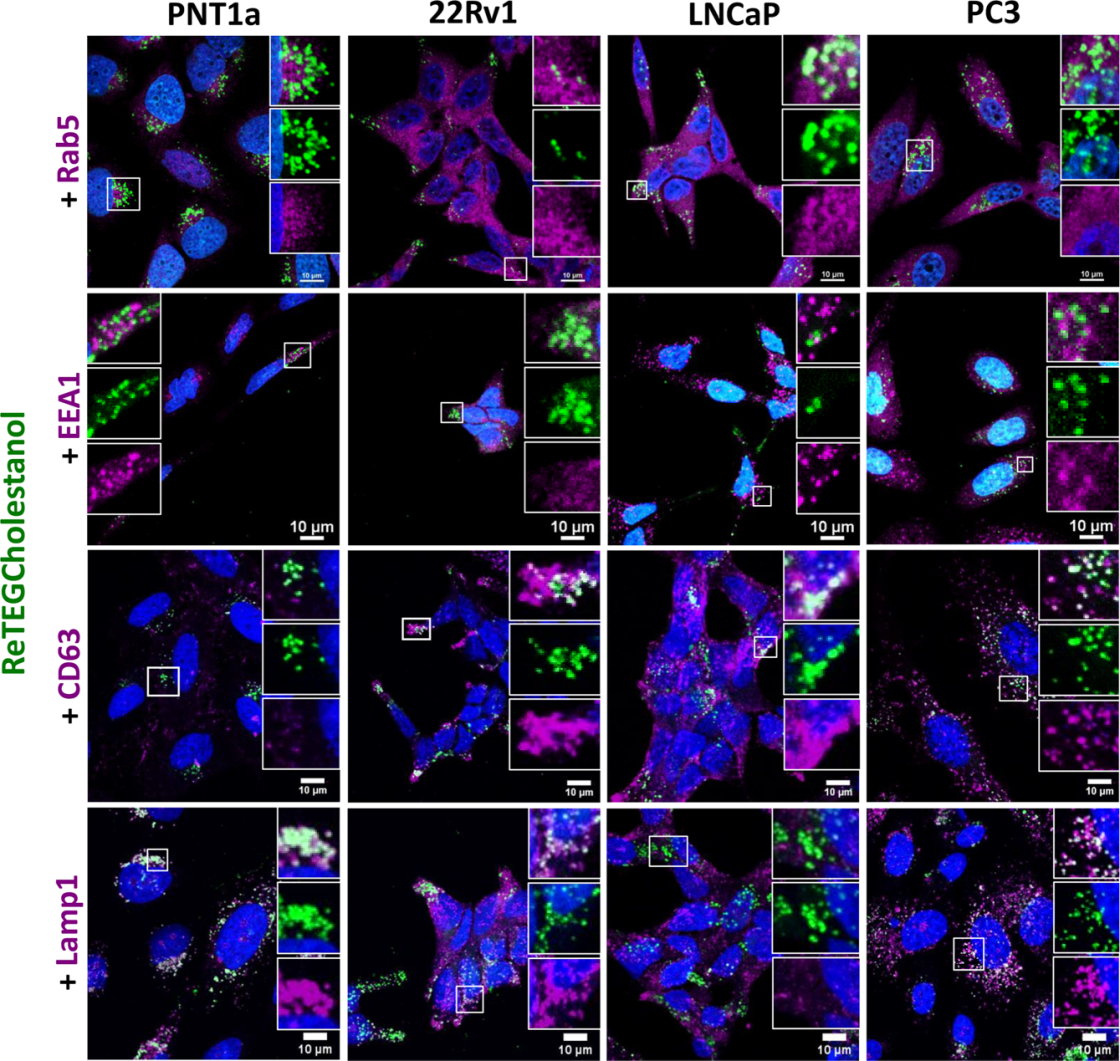
ReTEGCholestanol co-localized with different endosome-lysosome compartments in non-malignant and malignant prostate cancer cells. Early endosomes were detected with anti-Rab5 and anti-EEA1 antibodies, while multivesicular bodies/late endosomes and lysosomes were detected with anti-CD63 and anti-Lamp1 antibodies, respectively. Serum-starved prostate cells were incubated for 24 h with ∼5 μM ReTEGCholestanol, then fixed, permeabilised and immunolabelled with specific antibodies. To avoid cross-talk, secondary antibodies conjugates with Alexa Fluor 647 (excitation/emission 647/670 nm) were used. Scale bars; 10 μm.

The steady state, endosomal compartment distribution of ReTEGCholestanol was different in the prostate cell lines after 24 h (Fig. 4). In non-malignant PNT1a cells the majority of ReTEGCholestanol co-localized with Lamp1 positive lysosomes, with very little co-localization observed in Rab5 and EEA1 early endosomes or CD63 late endosomes/multivesicular bodies (Fig. 4), which was substantiated by M1 Mander's co-localization coefficient analysis (Table 1). However, not all Lamp1 positive lysosomes in PNT1a cells contained ReTEGCholestanol. In prostate cancer cell lines, ReTEGCholestanol co-localized with some Rab5-early endosomes, but not with EEA1-early endosomes (Fig. 4). This was substantiated by the Mander's M1 coefficient with the highest co-localization coefficient between ReTEGCholestanol and Rab5 reported for 22Rv1 cells (M1 = 0.454 ± 0.059) followed by LNCaP cells and PC3 cells, respectively, (M1 = 0.399 ± 0.077; M1 = 0.349 ± 0.125; Table 1). Co-localization was also observed with some CD63-positive late endosomes/multivesicular bodies in all of the prostate cancer cell lines, but this was not observed in PNT1a cells (Fig. 4). LNCaP cells showed the highest co-localization coefficient between ReTEGCholestanol and CD63 (M1 = 0.541 ± 0.097), followed by PC3 and 22Rv1 cells (M1 = 0.536 ± 0.156, M1 = 0.438 ± 0.095, respectively), with PNT1a cells having minimal co-localization (M1 = 0.174 ± 0.143) (Table 1). Consistent with LysoTracker staining, the lysosomal marker Lamp1 showed significant co-localization with ReTEGCholestanol in PC3 cells, but this was not evident in LNCaP cells (Fig. 4; Table 1). In 22Rv1 cells ReTEGCholestanol was co-localized with Lamp1-positive lysosomes (Fig. 4; Table 1), but interestingly this was less apparent with LysoTracker staining (Fig. 3). Although accumulation of ReTEGCholestanol was demonstrated in some Rab5-early endosomes, CD63-late endosomes/multivesicular bodies and lysosomes in different prostate cell lines, not all of these endosomal compartments were ReTEGCholestanol positive, which was evident by the low M2 Mander's co-localization coefficients (Table 1). Taken together, these data suggest that in non-malignant, PNT1a cells ReTEGCholestanol is being primarily trafficked to and accumulates in end stage lysosomal vesicles. In contrast an altered endosomal trafficking pathway in prostate cancer cell lines may contribute to reduced trafficking of ReTEGCholestanol to lysosomes and increased accumulation in a subset of early endosomes and late endosomes/multivesicular bodies.

**Table 1:** Mander's co-localization coefficients (M1; ReTEGCholestanol co-localized to a given marker; and M2; marker co-localized to ReTEGCholestanol) for ReTEGCholestanol and endo-lysosomal markers.

Cell line		PNT1a	22Rv1	LNCaP	PC3
**Rab5**	M1	0.251 ± 0.067	0.454 ± 0.059	0.399 ± 0.077	0.346 ± 0.125
	M2	0.048 ± 0.040	0.04 ± 0.010	0.027 ± 0.015	0.029 ± 0.018
**EEA1**	M1	0.047 ± 0.009	0.173 ± 0.049	0.234 ± 0.090	0.092 ± 0.048
	M2	0.008 ± 0.002	0.052 ± 0.041	0.055 ± 0.037	0.040 ± 0.025
**CD63**	M1	0.130 ± 0.082	0.438 ± 0.095	0.541 ± 0.097	0.536 ± 0.156
	M2	0.054 ± 0.030	0.110 ± 0.040	0.173 ± 0.027	0.257 ± 0.097
**Lamp1**	M1	0.933 ± 0.032	0.622 ± 0.073	0.307 ± 0.086	0.734 ± 0.105
	M2	0.386 ± 0.163	0.186 ± 0.097	0.061 ± 0.016	0.181 ± 0.055

Data presented was the mean of five images ± SD

## Discussion

The trafficking of fluorescently-tagged cholesterol analogs has previously been demonstrated, and with each different derivitization the probes delineate some, but not all, of the complex biology of cholesterol function and homeostasis.^24,39^ The delivery of lipophilic cholesterol tagged molecules or fluorescent cholesterol mimics is a significant challenge for live cell imaging, due to the aqueous extracellular environment required for cell survival, but also because of the multifunctionality of cholesterol and its diversity of molecular interactions. The attachment of a fluorescence tag such as BODPIY to cholesterol via the 3’ OH group has demonstrated some level of success in producing soluble compounds that can be internalized into cells.^21,27^ Our results indicate that direct attachment of our luminescent Re(I) tag to cholesterol or cholestanol *via* the 3’ OH group resulted in low solubility, which was not suitable for live cell imaging. The incorporation of a TEG linker greatly enhanced the solubility of the cholesterol and cholestanol compounds, however, only **ReTEGCholestanol** was ultimately deemed suitable for cell biology investigations in culture medium. The intracellular uptake of cholesterol mimics can be improved by complexing them with MβCD to improve solubility, however, this process is time consuming, the targeting may be altered and the complexes require careful storage.^12,13^ This investigation focused on the synthesis of cholesterol related compounds that could be delivered without the use of a surrogate structure and thus focused on **ReTEGCholestanol**, which had less solubility issues than **ReTEGCholesterol** and either directly coupled **ReCholestanol or ReCholesterol**. The ability to use a standard live cell staining protocol for a cholesterol mimic, to analyse cholesterol uptake and delivery offers distinct analytical, time and cost advantages, averting the need for additional preparation steps and costly complexing reagents. Furthermore, **ReTEGCholestanol** proved functional for both live cell imaging and fixed cell applications, making it compatible with a variety of experimental protocols and multicolour imaging with, for example, antibodies and other molecular imaging technologies. The use of the rhenium luminescent tag affords a number of other advantages over traditional organic fluorophores including, reduced photobleaching, a large Stokes shift, long emissions lifetime^29^ and compatibility with multimodal imaging platforms.^29,31–37^ Thus, **ReTEGCholestanol** is amenable to a vast array of experimental approaches, which can be particularly useful in the study of lipids that are difficult to detect by traditional fluorescence imaging and many lipids are not compatible with fixation/permeabilization protocols. Producing a range of different functionalized cholesterol derivatives offers the potential to dissect different aspects of cholesterol biology, but not all of these compounds have functional utility, as demonstrated here with cholestanol and cholesterol linked directly to rhenium complexes.

**ReTEGCholestanol** had a similar cellular distribution reported for other cholesterol mimics, with uptake and delivery to endosomes.^20,24,63^ In non-malignant prostate cell line PNT1a, **ReTEGCholestanol** primarily accumulated in late endosomal/lysosomal compartments, detected by LysoTracker and Lamp1-positive antibody staining (Fig. 2 and 4). This matches with the localization of a recently reported rhodamine-tagged cholestanol label^24^ and BODIPY-cholesterol attached at C24.^20^ We did not observe **ReTEGCholestanol** localization to lipid droplets, as esterification of this compound is unlikely due to the position of the rhenium tag at hydroxy group of cholestanol. The **ReTEGCholestanol** was also not detected in mitochondria, which may therefore also be dependent upon the hydroxyl region of the cholesterol being available. Significant amounts of the **ReTEGCholestanol** were not observed at the plasma membrane, with the exception of 22RV1 cells, which may suggest that the normal trafficking from the endosome-lysosome system to the cell surface^6,7^ is also restricted. The absence of **ReTEGCholestanol** at the plasma membrane in PNT1a cells, may also result from a transient association under normal conditions, which was not detected at the single time point investigated, but which could be visualized when trafficking was altered as in the case of 22RV1 cells. The cellular uptake and delivery of **ReTEGCholestanol** in all of the prostate cell lines, however, indicated that it is being incorporated into the endosome and lysosome system. The significantly higher accumulation of **ReTEGCholestanol**, as assessed by luminescence intensity, in androgen independent PC3 cells compared to androgen dependent 22Rv1 and LNCaP cells is consistent with increased lipid metabolism and altered cholesterol biology in advanced cancer. This is also in agreement with previous reports of increased cholesterol uptake in advanced prostate cancer cells.^64–66^ While our luminescence intensity studies provide qualitative evidence of **ReTEGCholestanol** uptake, to precisely quantify the intracellular levels of the compound will require inductively coupled plasma mass spectrometry (ICP-MS) analysis. We concluded that while certain functional aspects of cholesterol were blocked by the Re TEG coupling to the 3’ OH site, the endocytic uptake and trafficking in the endosome-lysosome system was still maintained, providing in effect an imaging agent to report on specific aspects of cholesterol biology/function, mainly endosome-lysosome traffic, but possible membrane incorporation. Thus, an alternate coupling of ReTEG to maintain the functional secondary 3’ OH of cholesterol, using, for example, the alkyl chain at the other end of the molecule and the tertiary alcohol in 25-hydroxycholesterol as the coupling site, may generate an imaging agent that can report on cholesterol lipid droplet and mitochondrial distribution. These different derivatives may be used to build a library of cholesterol related compounds that enable studies on specific aspects of cholesterol function.

As **ReTEGCholestanol** is suitable not only for live cell imaging but is also retained in cells after fixation and permeabilization, we were able to perform immunofluorescence after **ReTEGCholestanol** treatment. To further explore the endosomal distribution of **ReTEGCholestanol** we investigated the co-localization of this imaging agent with specific endosome-lysosome sub-compartment markers. The **ReTEGCholestanol** was detected in Rab5 early endosomes, CD63 late endosomes/multivesicular bodies and Lamp1 endosomes-lysosomes in different cells demonstrating that the imaging agent is being effectively trafficked to different endosome-lysosome sub-compartments. After 24 h of uptake most of the **ReTEGCholestanol** was detected in the lysosomes of non-malignant PNT1a cells. However, endosome biogenesis is significantly altered in the prostate cancer, with both modified compartment distribution, and reduced endosome compartment mediated transferring trafficking to the nucleus reported in 22Rv1 and LNCaP cell lines.^62^ The reduced trafficking of **ReTEGCholestanol** to the lysosome and increased detection in endosomal compartments of the prostate cancer cells is therefore consistent with the altered endosome dynamics in prostate cancer. Interestingly, the **ReTEGCholestanol** did detect Lamp1 positive lysosomes in 22Rv1 prostate cancer cells, but there was minimal LysoTracker staining, suggesting reduced lysosome acidification, which can also contribute to altering the positioning and traffic of endosome-lysosome compartments.^67^ The increased localization of **ReTEGCholestanol** to CD63-positive structures (CD63 is a biomarker of multivesicular bodies and exosomes) in malignant prostate cancer cells as compared to non-malignant cells is consistent with reports of exosomes from breast and prostate cancer being enriched in cholesterol.^68,69^ Cholesterol is required for exosomes biogenesis,^70^ and this biology can facilitate intercellular communication^71–73^ in cancer cells; and in future studies we therefore plan to investigate **ReTEGCholestanol** distribution in exosomes.

The **ReTEGCholestanol** is a new cholesterol mimic with advantageous luminescence properties provided by rhenium-based complex. It proved to effectively monitor endosome-lysosome traffic in both live and fixed cells using luminescence imaging, but may have other potential applications in techniques such as vibrational microscopy. The 3’ OH derivitized **ReTEGCholestanol** has the potential to selectively study the endosomal handling of sterols and the integration of cholesterol into endosome/lysosome compartments, and may be used to provide biological insight into a range of pathologies in other diseases besides prostate cancer.

### Patents

Aspects of the Re platform and lipid imaging applications have been patented (PCT/AU2015/000159: Brooks D, Plush S, Massi M. ‘Methods and products for labelling lipids’).

## Supplementary Material

mfac040_Supplemental_File

## Data Availability

The datasets generated during and/or analysed during the current study are available from the corresponding author on reasonable request.

## References

[1] Narwal V., Deswal R., Batra B., Kalra V., Hooda R., Sharma M., Rana J. S., Cholesterol biosensors: a review, Steroids, 2019, 143, 6–17. 30543816 10.1016/j.steroids.2018.12.003

[2] Chiang J. Y. L. , Bile acid metabolism and signaling, Compr. Physiol., 2013, 3 (3), 1191–1212. 23897684 10.1002/cphy.c120023 PMC4422175

[3] Prabhu A. V., Luu W., Li D., Sharpe L. J., Brown A. J., DHCR7: a vital enzyme switch between cholesterol and vitamin D production, Prog. Lipid Res., 2016, 64, 138–151. 27697512 10.1016/j.plipres.2016.09.003

[4] Luo J., Yang H., Song B.-L., Mechanisms and regulation of cholesterol homeostasis, Nat. Rev. Mol. Cell Biol., 2020, 21 (4), 225–245. 31848472 10.1038/s41580-019-0190-7

[5] Ikonen E. , Cellular cholesterol trafficking and compartmentalization, Nat. Rev. Mol. Cell Biol., 2008, 9 (2), 125–138. 18216769 10.1038/nrm2336

[6] Luo J., Jiang L., Yang H., Song B.-L., Routes and mechanisms of post-endosomal cholesterol trafficking: a story that never ends, Traffic, 2017, 18 (4), 209–217. 28191915 10.1111/tra.12471

[7] Trinh M. N., Brown M. S., Goldstein J. L., Han J., Vale G., McDonald J. G., Seemann J., Mendell J. T., Lu F., Last step in the path of LDL cholesterol from lysosome to plasma membrane to ER is governed by phosphatidylserine, Proc. Natl. Acad. Sci., 2020, 117 (31), 18521–18529. 32690708 10.1073/pnas.2010682117 PMC7414171

[8] Kuzu O. F., Noory M. A., Robertson G. P., The role of cholesterol in cancer, Cancer Res., 2016, 76 (8), 2063–2070. 27197250 10.1158/0008-5472.CAN-15-2613 PMC5813477

[9] Pelton K., Freeman M. R., Solomon K. R., Cholesterol and prostate cancer, Curr. Opin. Pharmacol., 2012, 12 (6), 751–759. 22824430 10.1016/j.coph.2012.07.006 PMC3515742

[10] Shafique K., McLoone P., Qureshi K., Leung H., Hart C., Morrison D. S., Cholesterol and the risk of grade-specific prostate cancer incidence: evidence from two large prospective cohort studies with up to 37 years’ follow up, BMC Cancer, 2012, 12 (1), 1–8. 22260413 10.1186/1471-2407-12-25 PMC3271031

[11] Stopsack K. H., Gerke T. A., Andrén O., Andersson S. O., Giovannucci E. L., Mucci L. A., Rider J. R., Cholesterol uptake and regulation in high-grade and lethal prostate cancers, Carcinogenesis, 2017, 38 (8), 806–811. 28595267 10.1093/carcin/bgx058 PMC6074871

[12] Maxfield F. R., Wüstner D., Analysis of cholesterol trafficking with fluorescent probes, in Methods in Cell Biology, Academic Press, 2012, vol. 108, pp. 367–393. 22325611 10.1016/B978-0-12-386487-1.00017-1 PMC3626500

[13] Mukherjee S., Zha X., Tabas I., Maxfield F. R., Cholesterol distribution in living cells: fluorescence imaging using dehydroergosterol as a fluorescent cholesterol analog, Biophys. J., 1998, 75 (4), 1915–1925. 9746532 10.1016/S0006-3495(98)77632-5 PMC1299862

[14] Fischer R. T., Stephenson F. A., Shafiee A., Schroeder F., Δ^5,7,9(11)^-cholestatrien-3β-ol: a fluorescent cholesterol analogue, Chem. Phys. Lipids., 1984, 36 (1), 1–14. 6518610 10.1016/0009-3084(84)90086-0

[15] Rogers J., Lee A. G., Wilton D. C., The organization of cholesterol and ergosterol in lipid bilayers based on studies using non-perturbing fluorescent sterol probes, Biochimica et Biophysica Acta (BBA) - Biomembranes, 1979, 552 (1), 23–37. 435495 10.1016/0005-2736(79)90243-8

[16] Wüstner D., Landt Larsen A., Færgeman N. J., Brewer J. R., Sage D., Selective visualization of fluorescent sterols in caenorhabditis elegans by bleach-rate-based image segmentation, Traffic, 2010, 11 (4), 440–454. 20070610 10.1111/j.1600-0854.2010.01040.x

[17] McIntosh A. L., Atshaves B. P., Huang H., Gallegos A. M., Kier A. B., Schroeder F., Fluorescence techniques using Dehydroergosterol to study cholesterol trafficking, Lipids, 2008, 43 (12), 1185–1208. 18536950 10.1007/s11745-008-3194-1 PMC2606672

[18] Wüstner D., Solanko L., Sokol E., Garvik O., Li Z., Bittman R., Korte T., Herrmann A., Quantitative assessment of sterol traffic in living cells by dual labeling with dehydroergosterol and BODIPY-cholesterol, Chem. Phys. Lipids., 2011, 164 (3), 221–235. 21291873 10.1016/j.chemphyslip.2011.01.004

[19] Li Z., Mintzer E., Bittman R., First synthesis of free cholesterol-BODIPY conjugates, J. Org. Chem., 2006, 71 (4), 1718–1721. 16468832 10.1021/jo052029x

[20] Hölttä-Vuori M., Uronen R. L., Repakova J., Salonen E., Vattulainen I., Panula P., Li Z., Bittman R., Ikonen E., BODIPY-cholesterol: a new tool to visualize sterol trafficking in living cells and organisms, Traffic, 2008, 9 (11), 1839–1849. 18647169 10.1111/j.1600-0854.2008.00801.x

[21] Rohrl C., Meisslitzer-Ruppitsch C., Bittman R., Li Z., Pabst G., Prassl R., Strobl W., Neumuller J., Ellinger A., Pavelka M., Stangl H., Combined light and electron microscopy using Diaminobenzidine Photooxidation to monitor trafficking of lipids derived from lipoprotein particles, Curr. Pharm. Biotechnol., 2012, 13 (2), 331–340. 21470121 10.2174/138920112799095338 PMC3855193

[22] Jansen M., Ohsaki Y., Rita Rega L., Bittman R., Olkkonen V. M., Ikonen E., Role of ORPs in sterol transport from plasma membrane to ER and lipid droplets in mammalian cells, Traffic, 2011, 12 (2), 218–231. 21062391 10.1111/j.1600-0854.2010.01142.x

[23] Bernecic N. C., Zhang M., Gadella B. M., Brouwers J. F. H. M., Jansen J. W. A., Arkesteijn G. J. A., de Graaf S. P., Leahy T., BODIPY-cholesterol can be reliably used to monitor cholesterol efflux from capacitating mammalian spermatozoa, Sci. Rep., 2019, 9 (1), 1–12. 31285440 10.1038/s41598-019-45831-7 PMC6614389

[24] Maiwald A., Bauer O., Gimpl G., Synthesis and characterization of a novel rhodamine labeled cholesterol reporter, Biochimica et Biophysica Acta (BBA) - Biomembranes, 2017, 1859 (6), 1099–1113. 28257814 10.1016/j.bbamem.2017.02.018

[25] Petrescu A. D., Vespa A., Huang H., McIntosh A. L., Schroeder F., Kier A. B., Fluorescent sterols monitor cell penetrating peptide Pep-1 mediated uptake and intracellular targeting of cargo protein in living cells, Biochimica et Biophysica Acta (BBA) - Biomembranes, 2009, 1788 (2), 425–441. 18992218 10.1016/j.bbamem.2008.09.015 PMC2680736

[26] Shrivastava S., Haldar S., Gimpl G., Chattopadhyay A., Orientation and dynamics of a novel fluorescent cholesterol analogue in membranes of varying phase, J. Phys. Chem. B, 2009, 113 (13), 4475–4481. 19249840 10.1021/jp808309u

[27] O'Connor D., Byrne A., Keyes T. E., Linker length in fluorophore-cholesterol conjugates directs phase selectivity and cellular localization in GUVs and live cells, RSC Adv., 2019, 9 (40), 22805–22816. 35514503 10.1039/c9ra03905h PMC9067298

[28] Ogawa Y., Tanaka M., A fluorescent cholesterol analogue for observation of free cholesterol in the plasma membrane of live cells, Anal. Biochem., 2016, 492, 49–55. 26366784 10.1016/j.ab.2015.09.003

[29] Lee L. C. C., Leung K. K., Lo K. K. W., Recent development of luminescent rhenium(i) tricarbonyl polypyridine complexes as cellular imaging reagents, anticancer drugs, and antibacterial agents, Dalton Trans., 2017, 46 (47), 16357–16380. 29110007 10.1039/c7dt03465b

[30] Hickey S. M., Ung B., Bader C., Brooks R., Lazniewska J., Johnson I. R. D., Sorvina A., Logan J., Martini C., Moore C. R., Karageorgos L., Sweetman M. J., Brooks D. A., Fluorescence microscopy—an outline of hardware, biological handling, and fluorophore considerations, Cells, 2021, 11 (1), 35. 35011596 10.3390/cells11010035 PMC8750338

[31] Bader C. A., Brooks R. D., Ng Y. S., Sorvina A., Werrett M. V., Wright P. J., Anwer A. G., Brooks D. A., Stagni S., Muzzioli S., Silberstein M., Skelton B. W., Goldys E. M., Plush S. E., Shandala T., Massi M., Modulation of the organelle specificity in Re(i) tetrazolato complexes leads to labeling of lipid droplets, RSC Adv., 2014, 4 (31), 16345–16351.

[32] Bader C. A., Carter E. A., Safitri A., Simpson P. V., Wright P., Stagni S., Massi M., Lay P. A., Brooks D. A., Plush S. E., Unprecedented staining of polar lipids by a luminescent rhenium complex revealed by FTIR microspectroscopy in adipocytes, Mol. Biosyst., 2016, 12 (7), 2064–2068. 27170554 10.1039/c6mb00242k

[33] Hostachy S., Policar C., Delsuc N., Re(I) carbonyl complexes: multimodal platforms for inorganic chemical biology, Coord. Chem. Rev., 2017, 351, 172–188.

[34] Henry L., Delsuc N., Laugel C., Lambert F., Sandt C., Hostachy S., Bernard A. S., Bertrand H. C., Grimaud L., Baillet-Guffroy A., Policar C., Labeling of Hyaluronic acids with a Rhenium-tricarbonyl tag and percutaneous penetration studied by multimodal imaging, Bioconjugate Chem., 2018, 29 (4), 987–991. 10.1021/acs.bioconjchem.7b00825 29360339

[35] Bader C. A., Shandala T., Carter E. A., Ivask A., Guinan T., Hickey S. M., Werrett M. V, Wright P. J., Simpson P. V, Stagni S., Voelcker N. H., Lay P. A., Massi M., Plush S. E., Brooks D. A., A molecular probe for the detection of polar lipids in live cells, PLoS One, 2016, 11 (8), e0161557. 27551717 10.1371/journal.pone.0161557 PMC4994960

[36] Wedding J. L., Harris H. H., Bader C. A., Plush S. E., Mak R., Massi M., Brooks D. A., Lai B., Vogt S., Werrett M. V, Simpson P. V, Skelton B. W., Stagni S., Intracellular distribution and stability of a luminescent rhenium(I) tricarbonyl tetrazolato complex using epifluorescence microscopy in conjunction with X-ray fluorescence imaging, Metallomics, 2017, 9 (4), 382–390. 27909710 10.1039/c6mt00243a

[37] Konkankit C. C., Lovett J., Harris H. H., Wilson J. J., X-Ray fluorescence microscopy reveals that rhenium(i) tricarbonyl isonitrile complexes remain intact: *In vitro*, Chem. Commun., 2020, 56 (48), 6515–6518. 10.1039/d0cc02451a 32432584

[38] Gillam T. A., Caporale C., Brooks R. D., Bader C. A., Sorvina A., Werrett M. V, Wright P. J., Morrison J. L., Massi M., Brooks D. A., Zacchini S., Hickey S. M., Stagni S., Plush S. E., Neutral Re(I) complex platform for Live intracellular imaging, Inorg. Chem., 2021, 60 (14), 10173–10185. 34210122 10.1021/acs.inorgchem.1c00418

[39] Huang H., McIntosh A. L., Atshaves B. P., Ohno-Iwashita Y., Kier A. B., Schroeder F., Use of dansyl-cholestanol as a probe of cholesterol behavior in membranes of living cells, J. Lipid Res., 2010, 51 (5), 1157–1172. 20008119 10.1194/jlr.M003244 PMC2853442

[40] Sun Q., Cai S., Peterson B. R., Practical synthesis of 3β-amino-5-cholestene and related 3β-halides involving i-steroid and retro-i-steroid rearrangements, Org. Lett., 2009, 11 (3), 567–570. 19115840 10.1021/ol802343z PMC2651230

[41] Boonyarattanakalin S., Martin S. E., Sun Q., Peterson B. R., A synthetic mimic of human Fc receptors: defined chemical modification of cell surfaces enables efficient endocytic uptake of human immunoglobulin-G, J. Am. Chem. Soc., 2006, 128 (35), 11463–11470. 16939269 10.1021/ja062377w PMC2528877

[42] Trinh T. T., Oswald L., Chan-Seng D., Lutz J. F., Synthesis of molecularly encoded oligomers using a chemoselective ‘aB + CD’ iterative approach, Macromol. Rapid Commun., 2014, 35 (2), 141–145. 24338828 10.1002/marc.201300774

[43] Lazniewska J., Agostino M., Hickey S. M., Parkinson-Lawrence E., Stagni S., Massi M., Brooks D. A., Plush S. E., Spectroscopic and molecular docking study of the interaction between neutral Re(I) tetrazolate complexes and bovine serum albumin, Chem. Eur. J., 2021, 27 (44), 11406–11417. 33960039 10.1002/chem.202101307

[44] Ha J. S., Ha C. E., Chao J. T., Petersen C. E., Theriault A., Bhagavan N. V., Human serum albumin and its structural variants mediate cholesterol efflux from cultured endothelial cells, Biochimica et Biophysica Acta (BBA) - Molecular Cell Research, 2003, 1640 (2–3), 119–128. 12729921 10.1016/s0167-4889(03)00027-2

[45] Sankaranarayanan S., De La Llera-Moya M., Drazul-Schrader D., Phillips M. C., Kellner-Weibel G., Rothblat G. H., Serum albumin acts as a shuttle to enhance cholesterol effl ux from cells, J. Lipid Res., 2013, 54 (3), 671–676. 23288948 10.1194/jlr.M031336 PMC3617942

[46] Sorvina A., Bader C. A., Caporale C., Carter E. A., Johnson I. R. D., Parkinson-Lawrence E. J., Simpson P. V., Wright P. J., Stagni S., Lay P. A., Massi M., Brooks D. A., Plush S. E., Lipid profiles of prostate cancer cells, Oncotarget, 2018, 9 (85), 35541–35552. 30473749 10.18632/oncotarget.26222 PMC6238979

[47] Dunn K. W., Kamocka M. M., McDonald J. H., A practical guide to evaluating colocalization in biological microscopy, Amer. Jour. of Phys.-Cell Phys., 2011, 300 (4), C723–C742. 10.1152/ajpcell.00462.2010 PMC3074624 21209361

[48] Sugandhi E. W., Slebodnick C., Falkinham J. O., Gandour R. D., Synthesis and antimicrobial evaluation of water-soluble, dendritic derivatives of epimeric 5α-cholestan-3-amines and 5α-cholestan-3-yl aminoethanoates, Steroids, 2007, 72 (8), 615–626. 17532019 10.1016/j.steroids.2007.04.001

[49] Zhang T., Hu X., Wang Z., Yang T., Sun H., Li G., Lu H., Carboxylate-assisted iridium-catalyzed C-H amination of arenes with biologically relevant alkyl azides, Chem. Eur. J., 2016, 22 (9), 2920–2924. 26712274 10.1002/chem.201504880

[50] Willibald J., Harder J., Sparrer K., Conzelmann K. K., Carell T., Click-modified anandamide siRNA enables delivery and gene silencing in neuronal and immune cells, J. Am. Chem. Soc., 2012, 134 (30), 12330–12333. 22812910 10.1021/ja303251f

[51] Suzuki Y., Yokoyama K., Development of functional fluorescent molecular probes for the detection of biological substances, Biosensors, 2015, 5 (2), 337–363. 26095660 10.3390/bios5020337 PMC4493553

[52] Resch-Genger U., Grabolle M., Cavaliere-Jaricot S., Nitschke R., Nann T., Quantum dots versus organic dyes as fluorescent labels, Nat. Methods, 2008, 5 (9), 763–775. 18756197 10.1038/nmeth.1248

[53] Yuan C., Gao J., Guo J., Bai L., Marshall C., Cai Z., Wang L., Xiao M., Dimethyl sulfoxide damages mitochondrial integrity and membrane potential in cultured astrocytes, PLoS One, 2014, 9 (9), e107447. 25238609 10.1371/journal.pone.0107447 PMC4169574

[54] Mah C. Y., Nassar Z. D., Swinnen J. V., Butler L. M., Lipogenic effects of androgen signaling in normal and malignant prostate, Asian Jour. of Uro., 7 (3), 258–270. 10.1016/j.ajur.2019.12.003 PMC7385522 32742926

[55] Namekawa T., Ikeda K., Horie-Inoue K., Inoue S., Application of prostate cancer models for preclinical study: advantages and limitations of cell lines, patient-derived xenografts, and three-dimensional culture of patient-derived cells, Cells, 2019, 8 (1), 74. 30669516 10.3390/cells8010074 PMC6357050

[56] Chen Y., Hughes-Fulford M., Human prostate cancer cells lack feedback regulation of low-density lipoprotein receptor and its regulator, SREBP2, Int. J. Cancer, 2001, 91 (1), 41–45. 11149418 10.1002/1097-0215(20010101)91:1<41::aid-ijc1009>3.0.co;2-2

[57] Wu X., Daniels G., Lee P., Monaco M. E., Lipid metabolism in prostate cancer, Am. J. Clin. Exp. Urol., 2014, 2 (2), 111–120. 25374912 PMC4219300

[58] Cunningham D., You Z., In vitro and in vivo model systems used in prostate cancer research, Jour. of Biol. Meth., 2015, 2 (1), e17. 10.14440/jbm.2015.63 PMC4487886 26146646

[59] Welte M. A., Wu C. C., Smith C., McCray B. A., Simpson T. I., Taylor J. P., Pennetta G., Hansen M., Yang F., Niebergall L. J., Al E., Expanding roles for lipid droplets, Curr. Biol., 2015, 25 (11), R470—R481. 26035793 10.1016/j.cub.2015.04.004 PMC4452895

[60] Ribas V., García-Ruiz C., Fernández-Checa J. C., Mitochondria, cholesterol, and cancer cell metabolism, Clin. Transl. Med., 2016, 5 (1), 22. 27455839 10.1186/s40169-016-0106-5 PMC4960093

[61] Meng Y., Heybrock S., Neculai D., Saftig P., Cholesterol handling in Lysosomes and beyond, Trends Cell Biol., 2020, 30 (6), 452–466. 32413315 10.1016/j.tcb.2020.02.007

[62] Ian D. A. B., Johnson R. D., Parkinson-Lawrence E. J., Shandala T., Weigert R., Butler L. M., Altered endosome biogenesis in prostate cancer has biomarker, Mol. Cancer Res., 2014, 12 (12), 1851–1862. 25080433 10.1158/1541-7786.MCR-14-0074 PMC4757910

[63] Szomek M., Moesgaard L., Reinholdt P., Haarhøj Hald S. B., Petersen D., Krishnan K., Covey D. F., Kongsted J., Wüstner D., Membrane organization and intracellular transport of a fluorescent analogue of 27-hydroxycholesterol, Chem. Phys. Lipids., 2020, 233, 105004. 33137329 10.1016/j.chemphyslip.2020.105004

[64] Schörghofer D., Kinslechner K., Preitschopf A., Schütz B., Röhrl C., Hengstschläger M., Stangl H., Mikula M., The HDL receptor SR-BI is associated with human prostate cancer progression and plays a possible role in establishing androgen independence, Repro. Biol. and Endo., 2015, 13 (1), 88, doi: 10.1186/s12958-015-0087-z. PMC4528807 26251134

[65] Allott E. H., Masko E. M., Freedland A. R., MacIas E., Pelton K., Solomon K. R., Mostaghel E. A., Thomas G. V., Pizzo S. V., Freeman M. R., Freedland S. J., Serum cholesterol levels and tumor growth in a PTEN-null transgenic mouse model of prostate cancer, Pro. Cancer Pro. Dis., 2018, 21 (2), 196–203. 10.1038/s41391-018-0045-x PMC6026483 29795142

[66] Gordon J. A., Noble J. W., Midha A., Derakhshan F., Wang G., Adomat H. H., Guns E. S. T., Lin Y. Y., Ren S., Collins C. C., Nelson P. S., Morrissey C., Wasan K. M., Cox M. E., Upregulation of scavenger receptor B1 is required for steroidogenic and nonsteroidogenic cholesterol metabolism in prostate cancer, Cancer Res., 2019, 79 (13), 3320–3331. 31064850 10.1158/0008-5472.CAN-18-2529 PMC6606386

[67] Glunde K., Guggino S. E., Solaiyappan M., Pathak A. P., Ichikawa Y., Bhujwalla Z. M., Extracellular acidification alters lysosomal trafficking in human breast cancer cells, Neoplasia, 2003, 5 (6), 533–545. 14965446 10.1016/s1476-5586(03)80037-4 PMC1502575

[68] Roberg-Larsen H., Lund K., Seterdal K. E., Solheim S., Vehus T., Solberg N., Krauss S., Lundanes E., Wilson S. R., Mass spectrometric detection of 27-hydroxycholesterol in breast cancer exosomes, J. Steroid Biochem. Mol. Biol., 2017, 169, 22–28. 26877254 10.1016/j.jsbmb.2016.02.006

[69] Llorente A., Skotland T., Sylvänne T., Kauhanen D., Róg T., Orłowski A., Vattulainen I., Ekroos K., Sandvig K., Molecular lipidomics of exosomes released by PC-3 prostate cancer cells, Biochimica et Biophysica Acta (BBA) - Mole. and Cell Bio. of Lipids, 2013, 1831 (7), 1302–1309. 10.1016/j.bbalip.2013.04.011 24046871

[70] Pfrieger F. W., Vitale N., Thematic review series: exosomes and microvesicles: lipids as key components of their biogenesis and functions, cholesterol, and the journey of extracellular vesicles, J. Lipid Res., 2018, 59 (12), 2255–2261. 29678958 10.1194/jlr.R084210 PMC6277151

[71] Lehmann B. D., Paine M. S., Brooks A. M., McCubrey J. A., Renegar R. H., Wang R., Terrian D. M., Senescence-associated exosome release from human prostate cancer cells, Cancer Res., 2008, 68 (19), 7864–7871. 18829542 10.1158/0008-5472.CAN-07-6538 PMC3845029

[72] Lorenc T., Klimczyk K., Michalczewska I., Słomka M., Kubiak-Tomaszewska G., Olejarz W., Exosomes in prostate cancer diagnosis, prognosis, and therapy, Int. J. Mol. Sci., 2020, 21 (6), 2118. 32204455 10.3390/ijms21062118 PMC7139716

[73] Vlaeminck-Guillem V. , Extracellular vesicles in prostate cancer carcinogenesis, diagnosis, and management, Front. Oncol., 2018, 8, 1. 29951375 10.3389/fonc.2018.00222 PMC6008571

