# Supplementary material for: Rhenium(I) conjugates as tools for tracking cholesterol in cells
Source: Metallomics. 2022 Jun 3;14(8):mfac040. doi: 10.1093/mtomcs/mfac040 (PMC9344854; doi:10.1093/mtomcs/mfac040)
Supplement: mfac040_Supplemental_File [file mfac040_supplemental_file.docx]

Rhenium(I) steroidal conjugates as tools for tracking cholesterol in cells.

Joanna Lazniewska ^1, ⸸^ , Christie Bader ^1, ⸸^ , Shane M. Hickey ^1^, Stavros Selemidis ^2^, John O’Leary ^3^, Peter V. Simpson ^4^, Stefano Stagni ^5^, Sally E. Plush ^1^, Massimiliano Massi ^4^ and Doug Brooks ^1,3,4^*

Email: doug.brooks@unisa.edu.au

**CONTENTS**

- ^1^H, ^13^C NMR spectra for all compounds (**Figures S1–S30**) p 2
- HRMS spectra for Re-complexes (**Figures S31-S34**) p 32
- Normalised absorbance and emission spectra for Re-complexes (**Figures S35-S38**) p 36


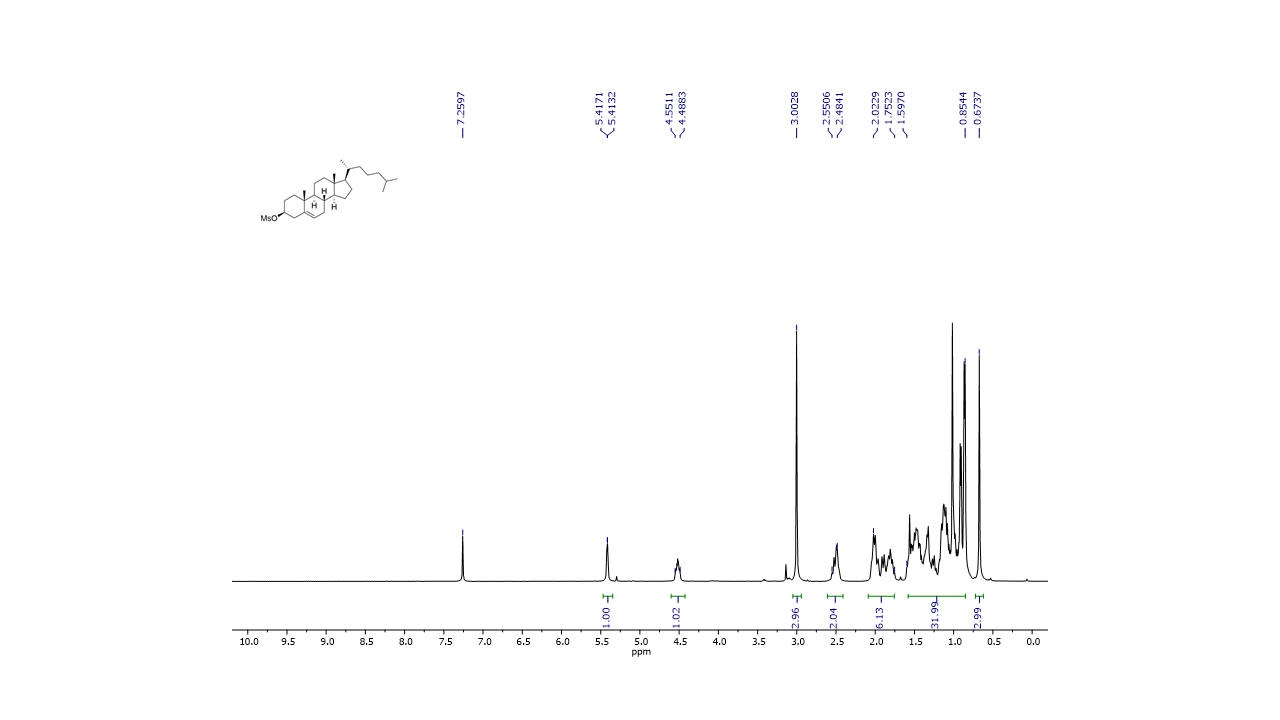


**Figure S1**: ^1^H NMR spectrum of **1** in CDCl_3_.


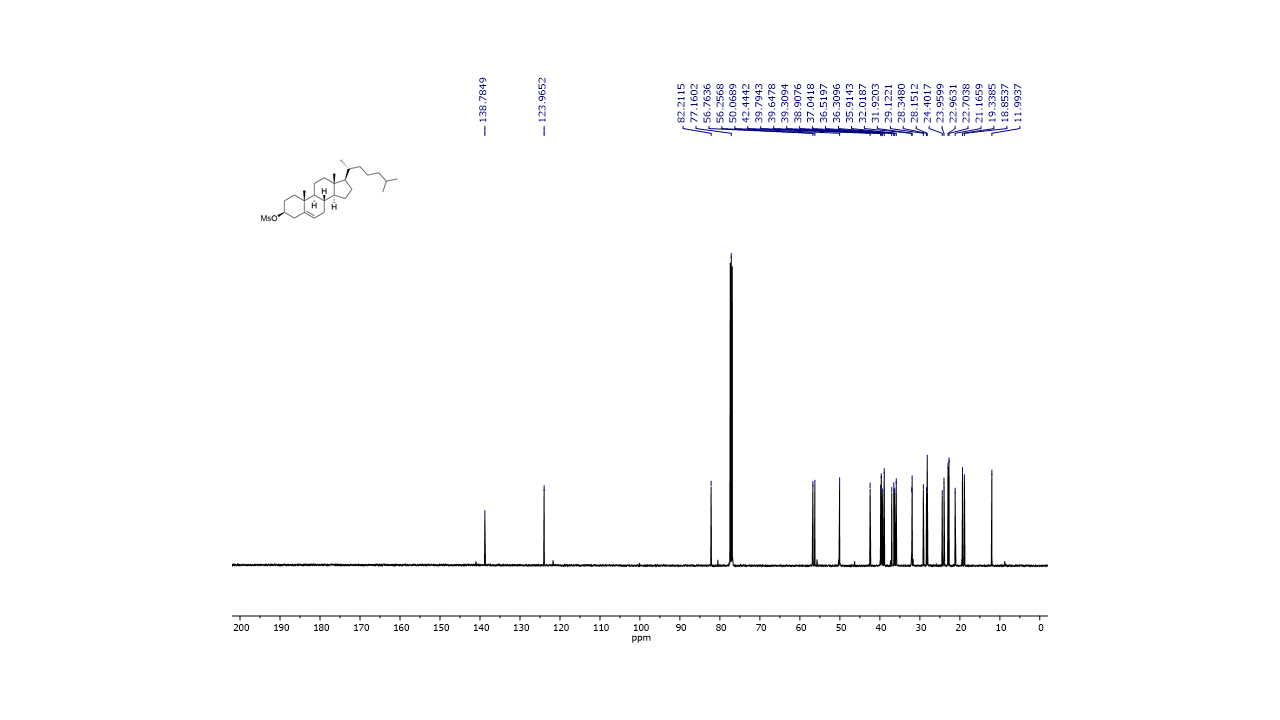


**Figure S2**: ^13^C NMR spectrum of **1** in CDCl_3_.


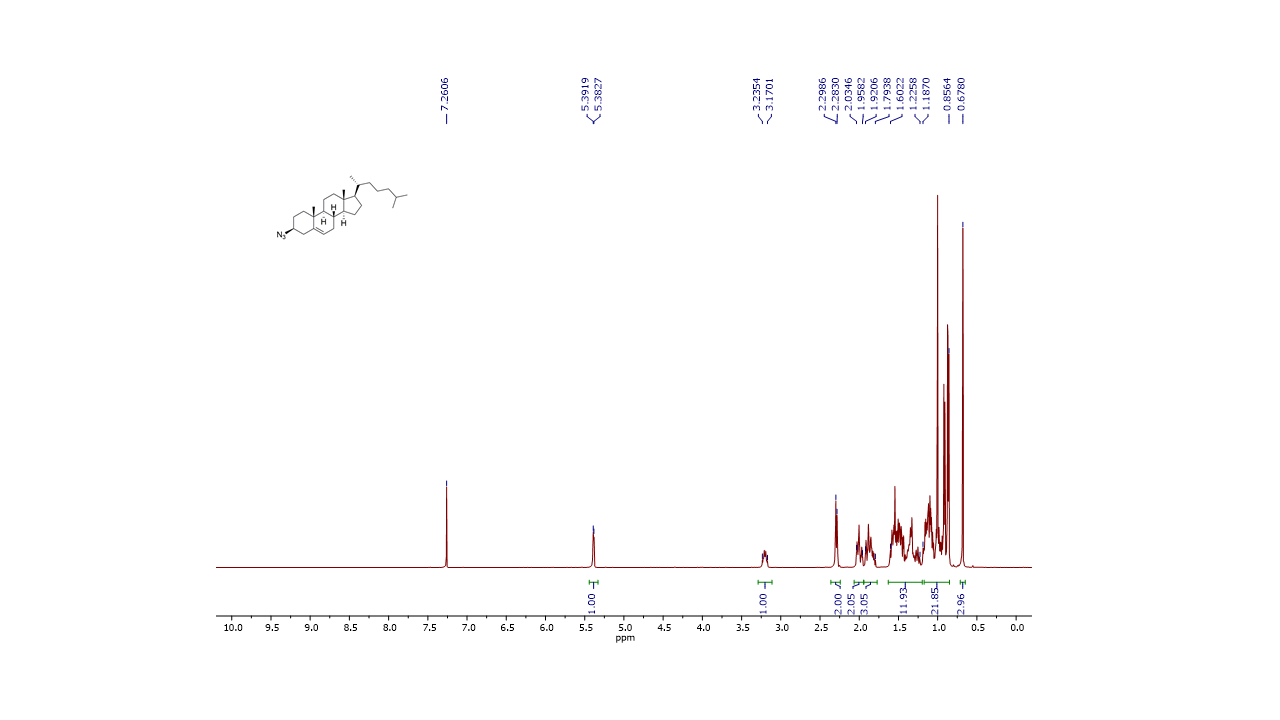


**Figure S3**: ^1^H NMR spectrum of **2** in CDCl_3_.


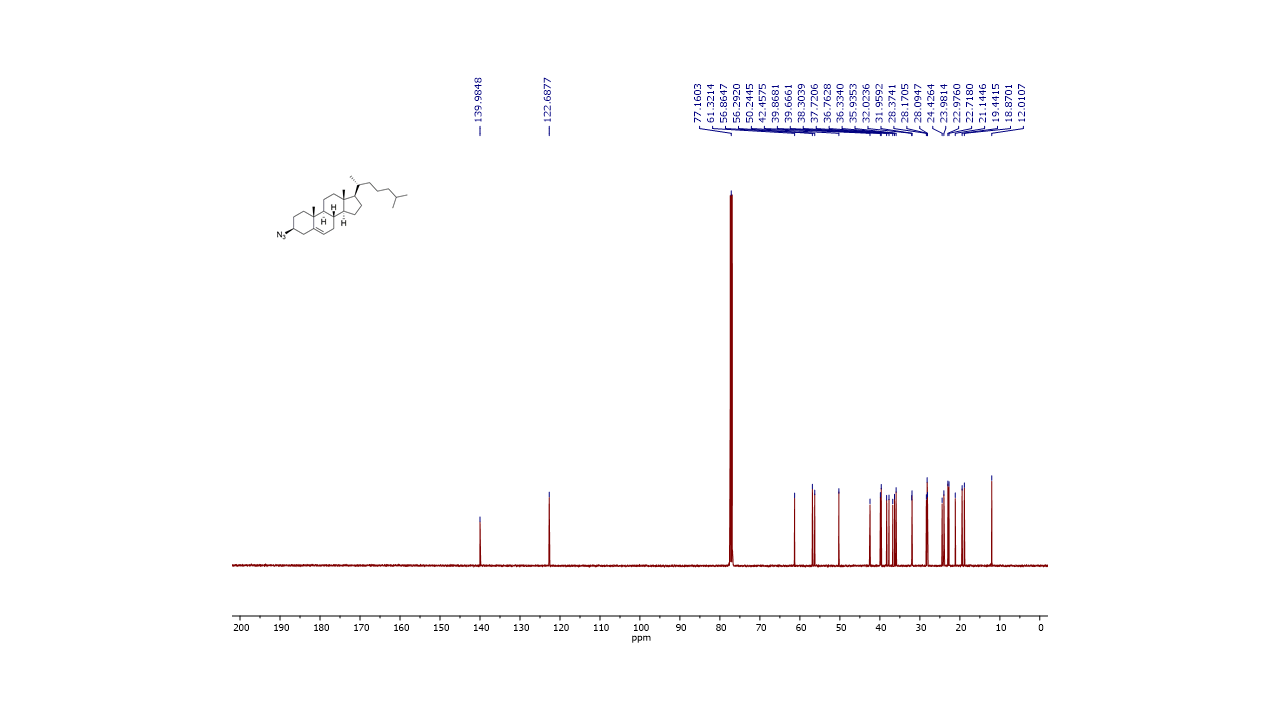


**Figure S4**: ^13^C NMR spectrum of **2** in CDCl_3_.


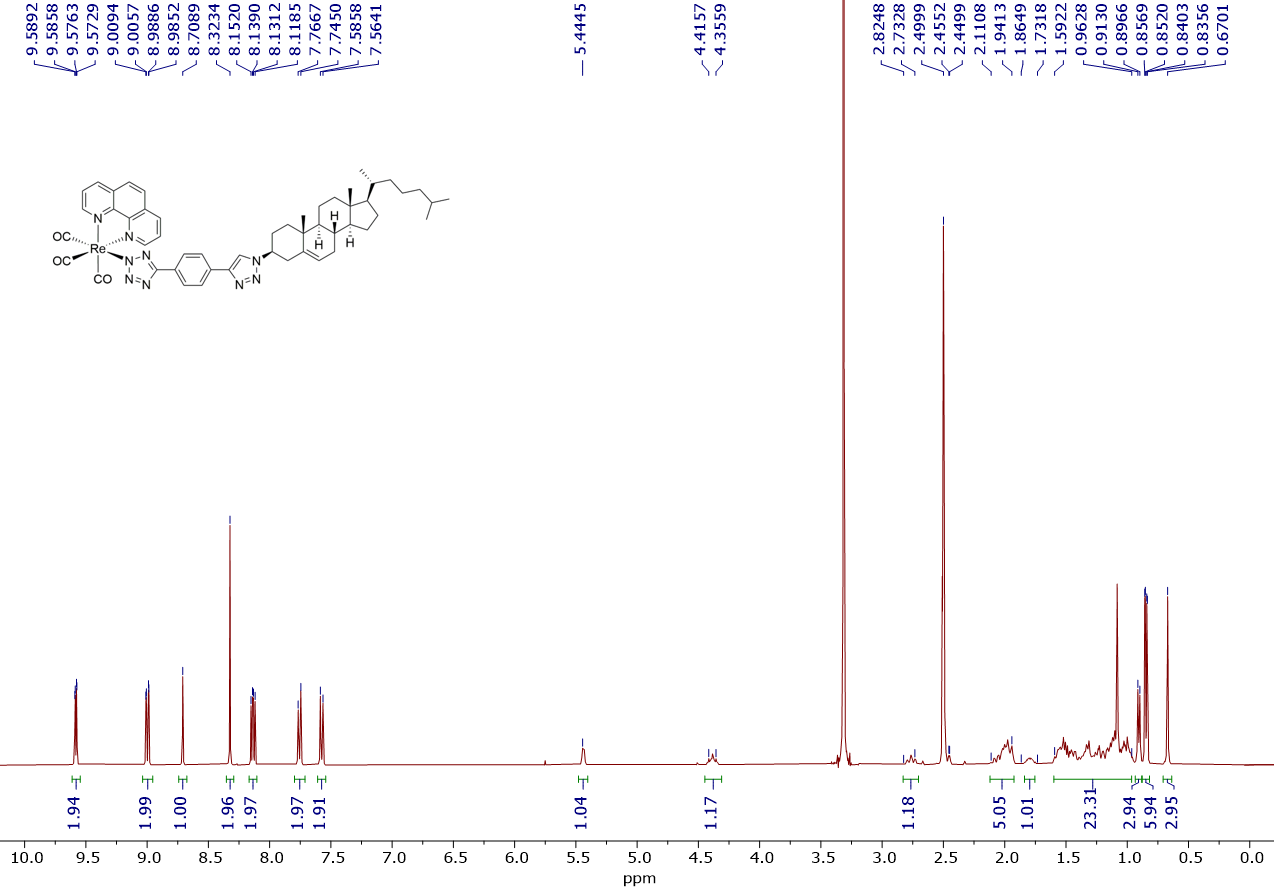


**Figure S5**: ^1^H NMR spectrum of **ReCholesterol** in DMSO-*d_6_*.


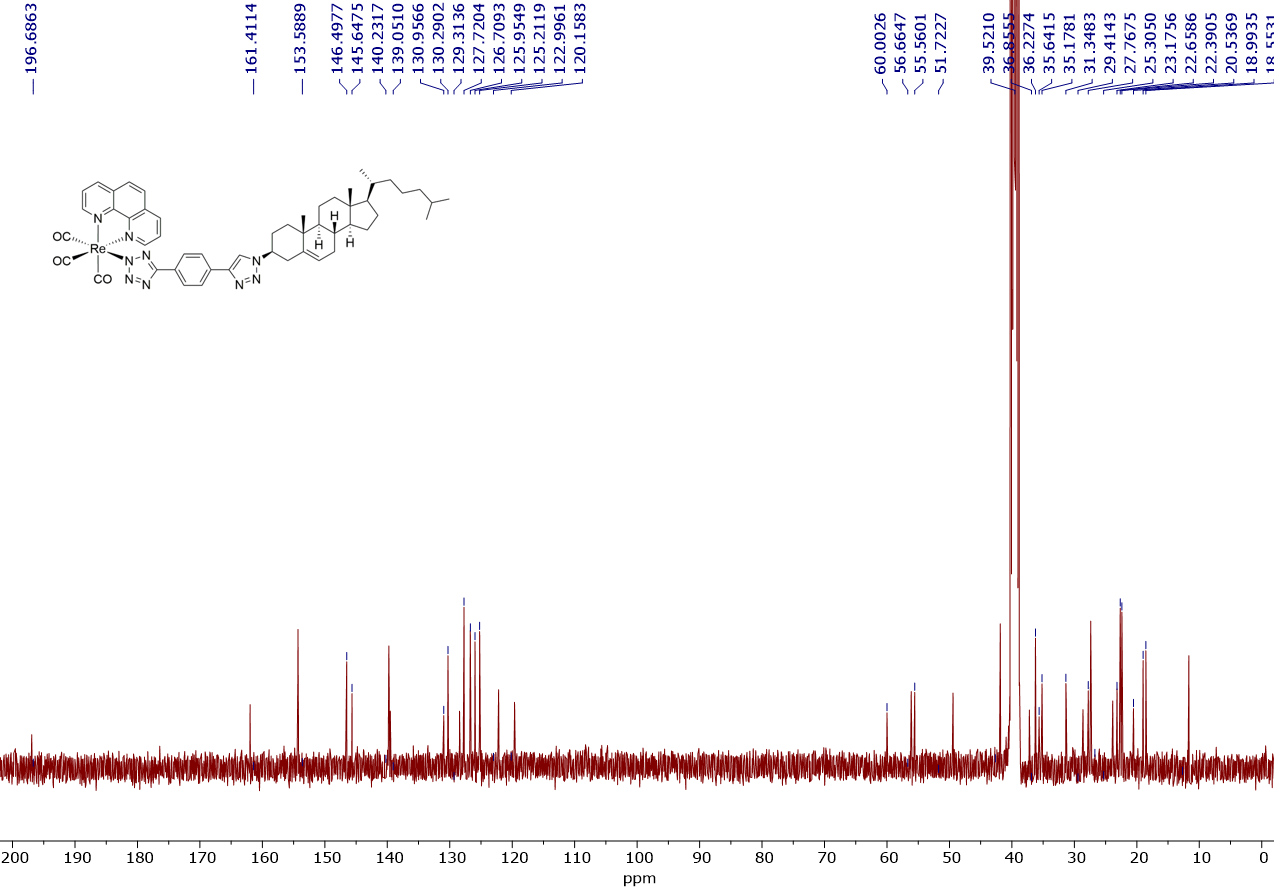


**Figure S6**: ^13^C NMR spectrum of **ReCholesterol** in DMSO-*d_6_*.


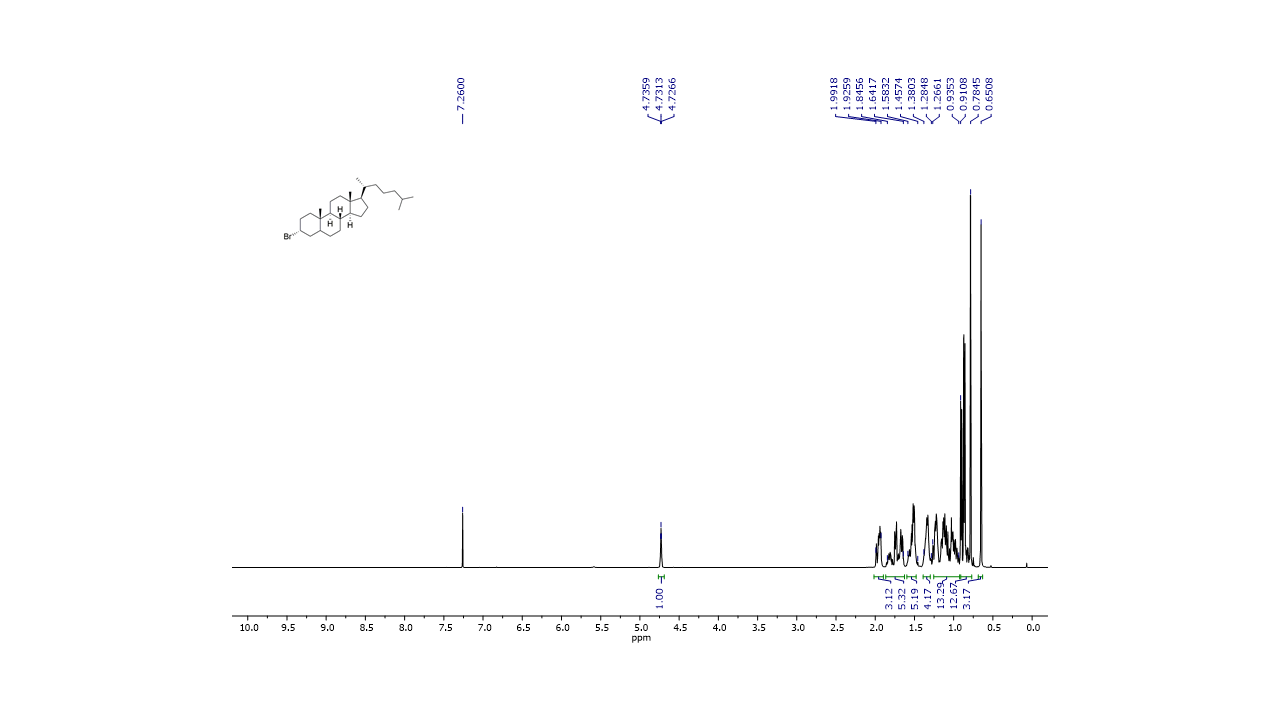


**Figure S7**: ^1^H NMR spectrum of **3** in CDCl_3_.


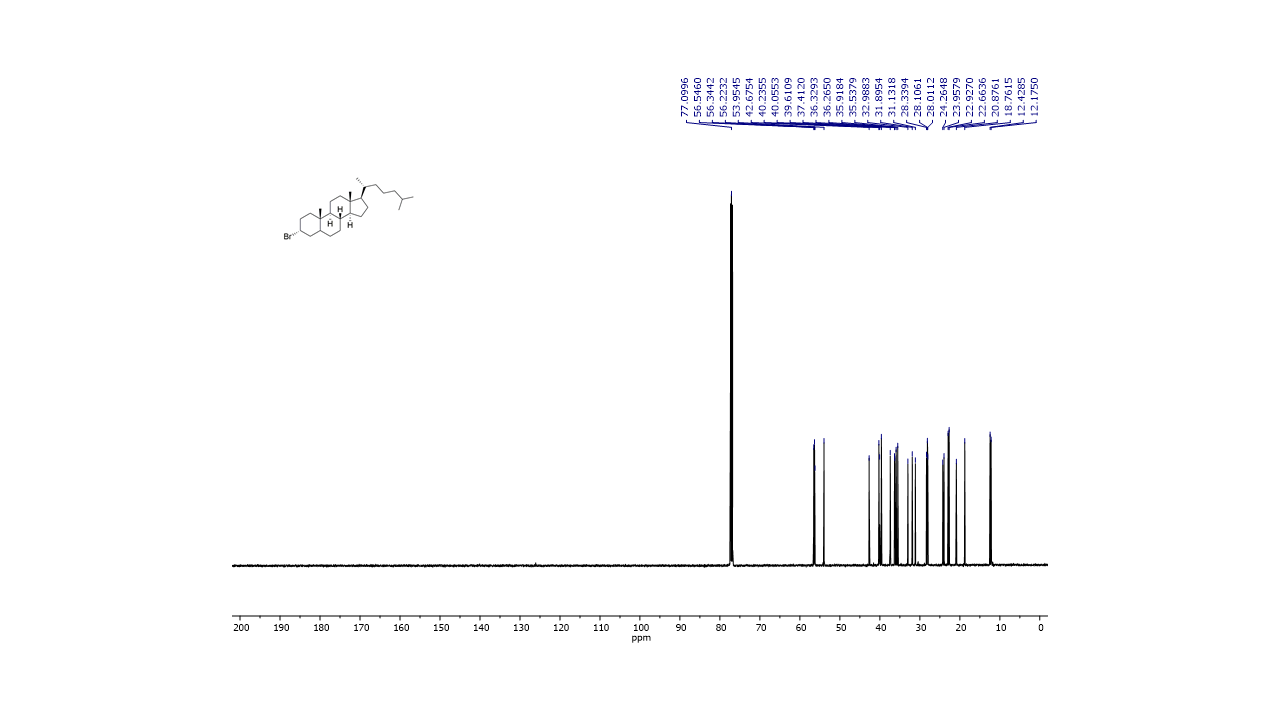


**Figure S8**: ^13^C NMR spectrum of **3** in CDCl_3_.


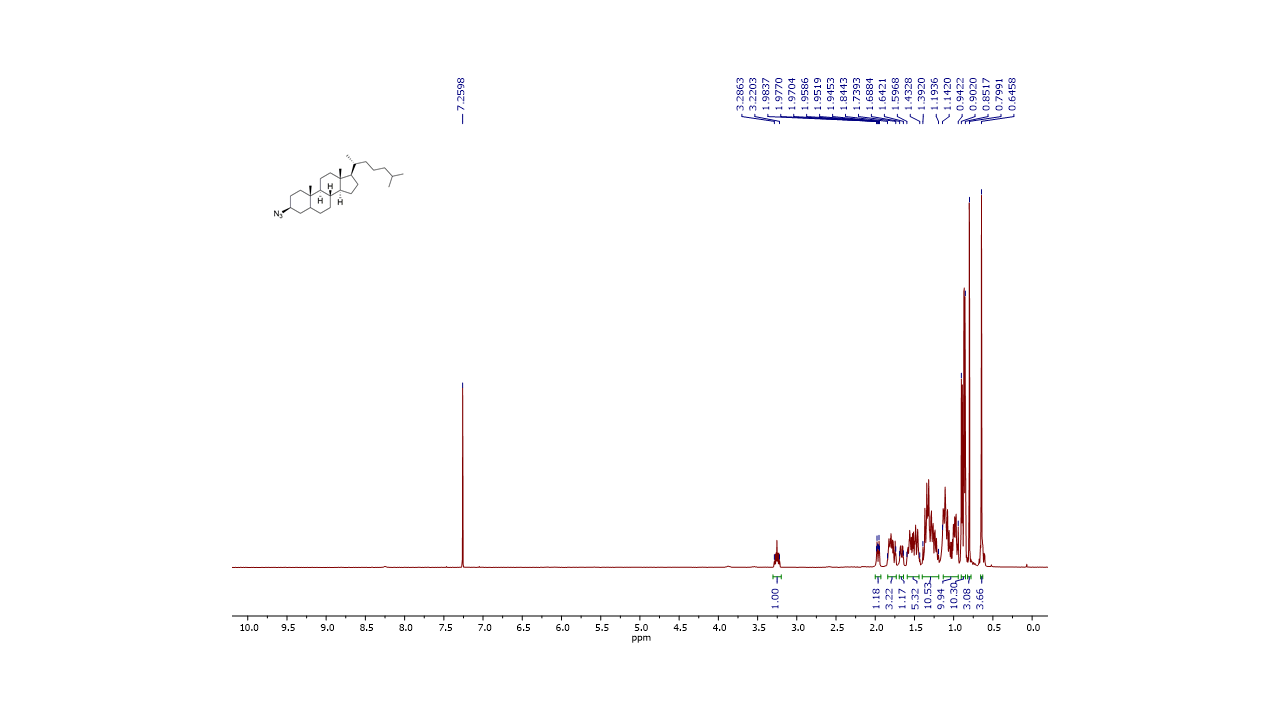


**Figure S9**: ^1^H NMR spectrum of **4** in CDCl_3_.


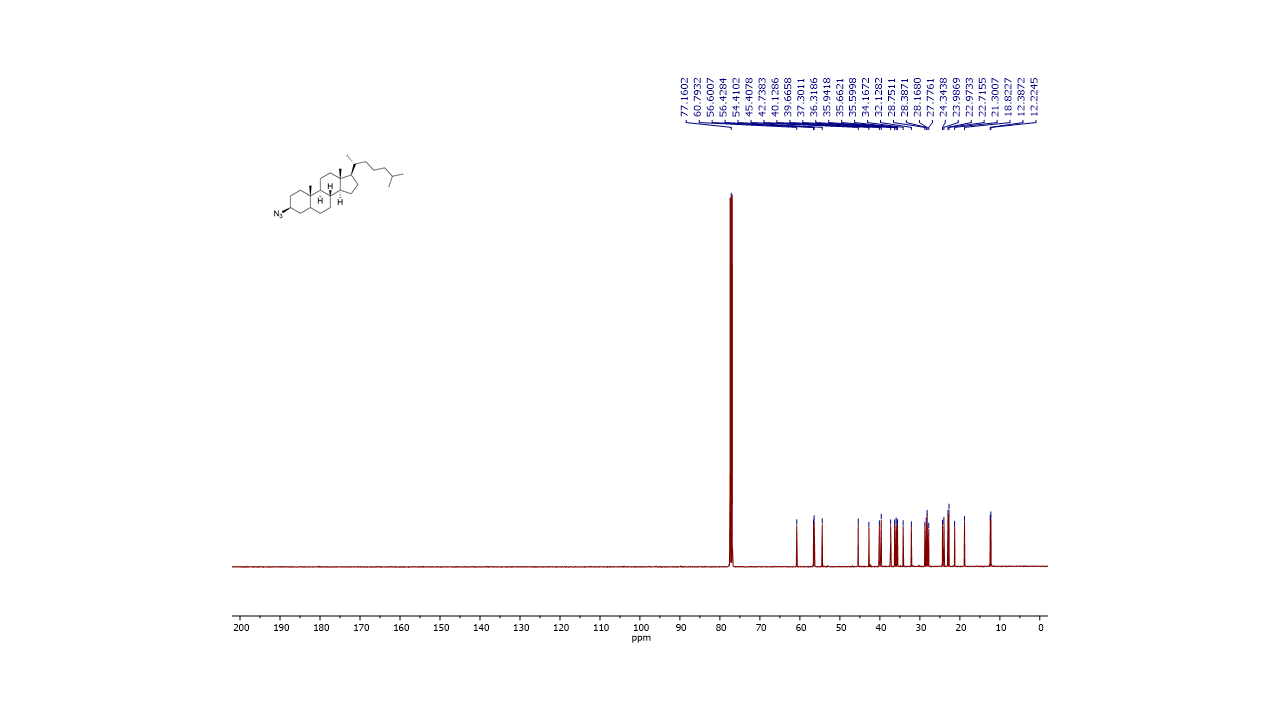


**Figure S10**: ^13^C NMR spectrum of **4** in CDCl_3_.


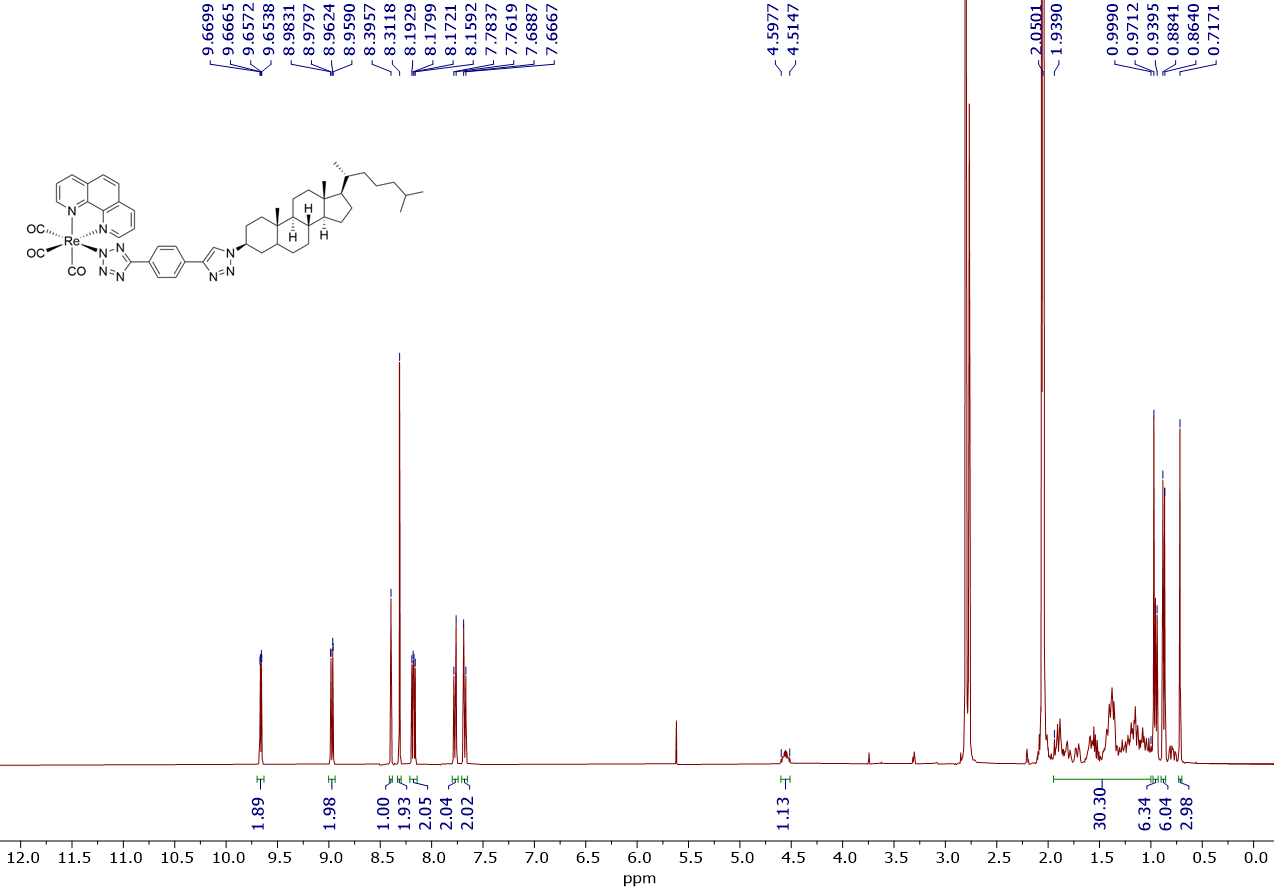


**Figure S11**: ^1^H NMR spectrum of **ReCholestanol** in acetone-*d_6_*.


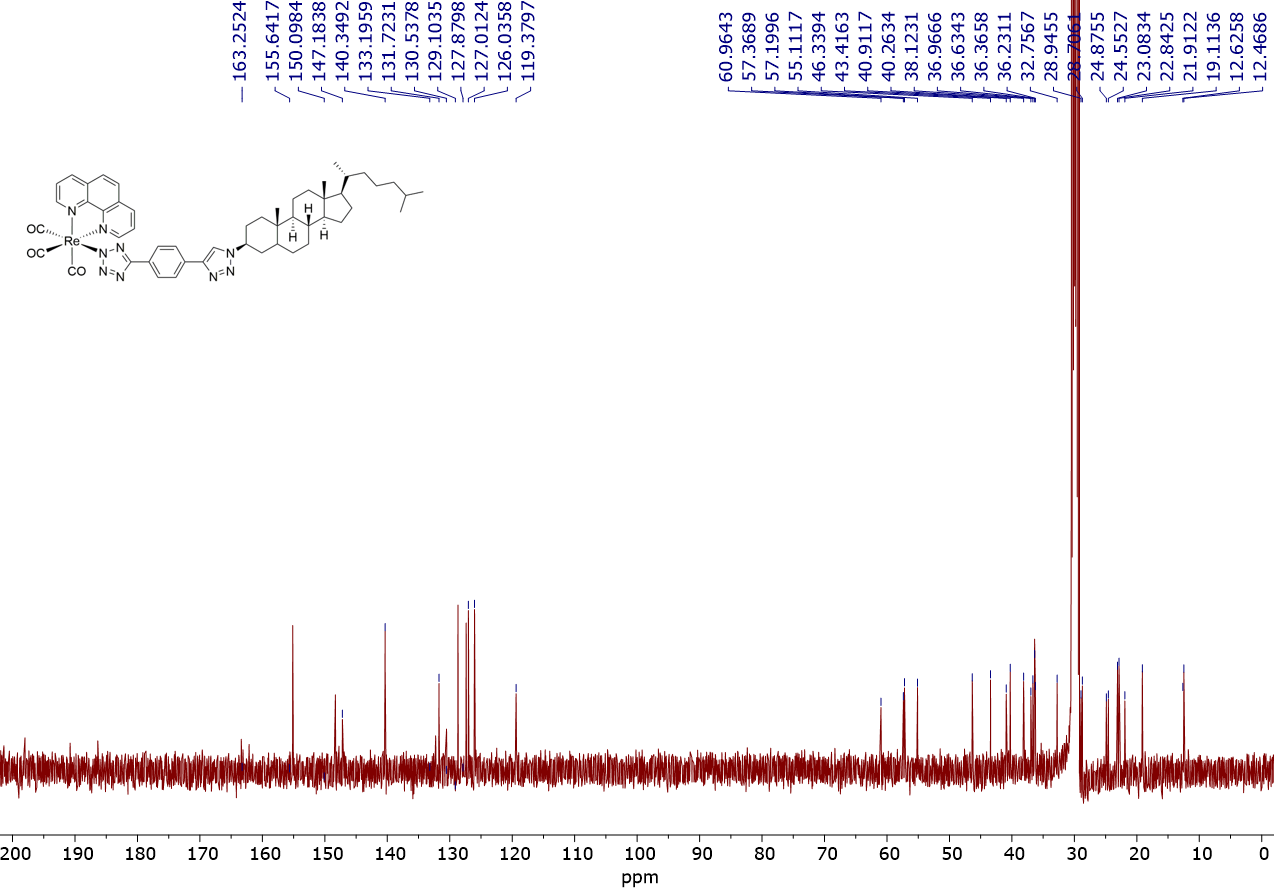


**Figure S12**: ^13^C NMR spectrum of **ReCholestanol** in acetone-*d_6_*.


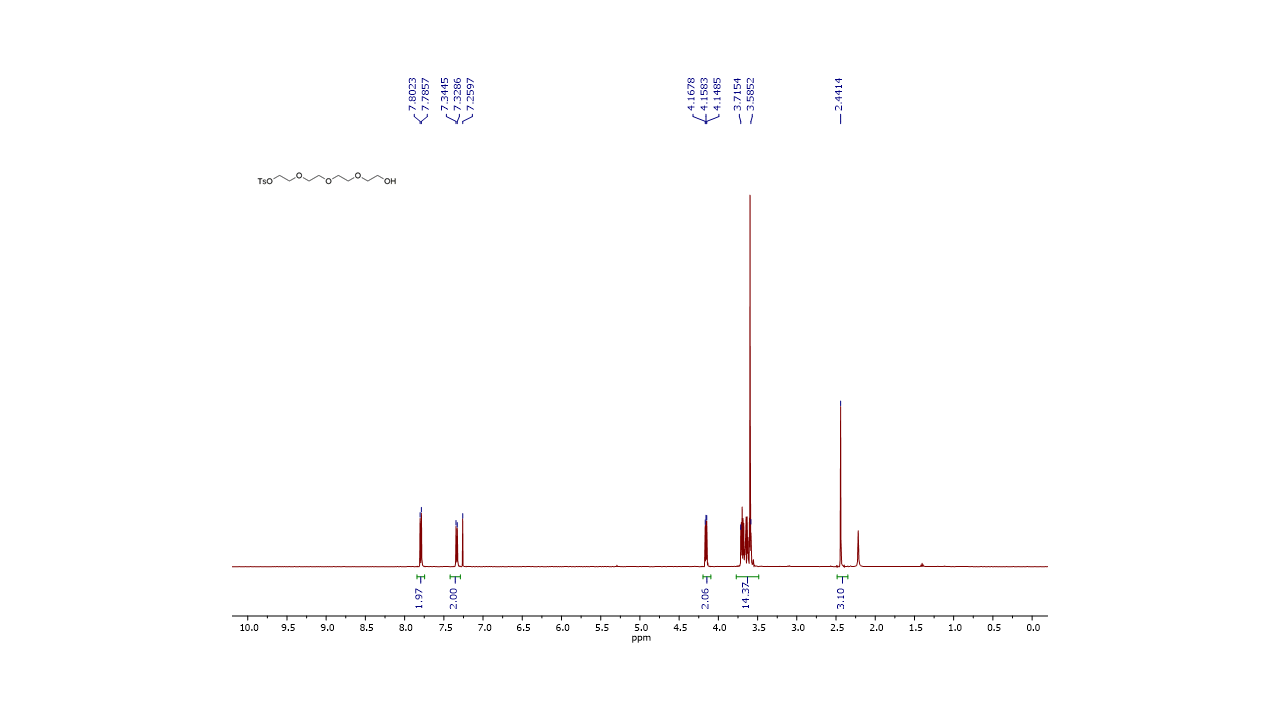


**Figure S13**: ^1^H NMR spectrum of **5** in CDCl_3_.


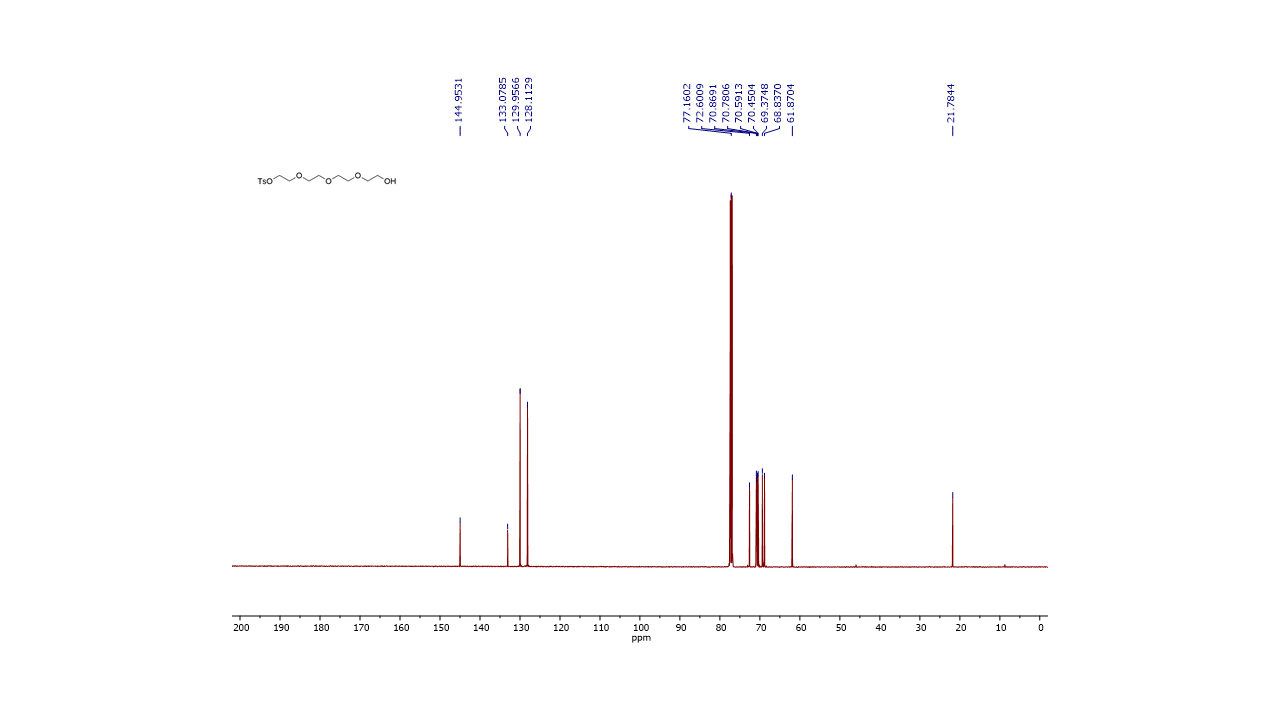


**Figure S14**: ^13^C NMR spectrum of **5** in CDCl_3_.


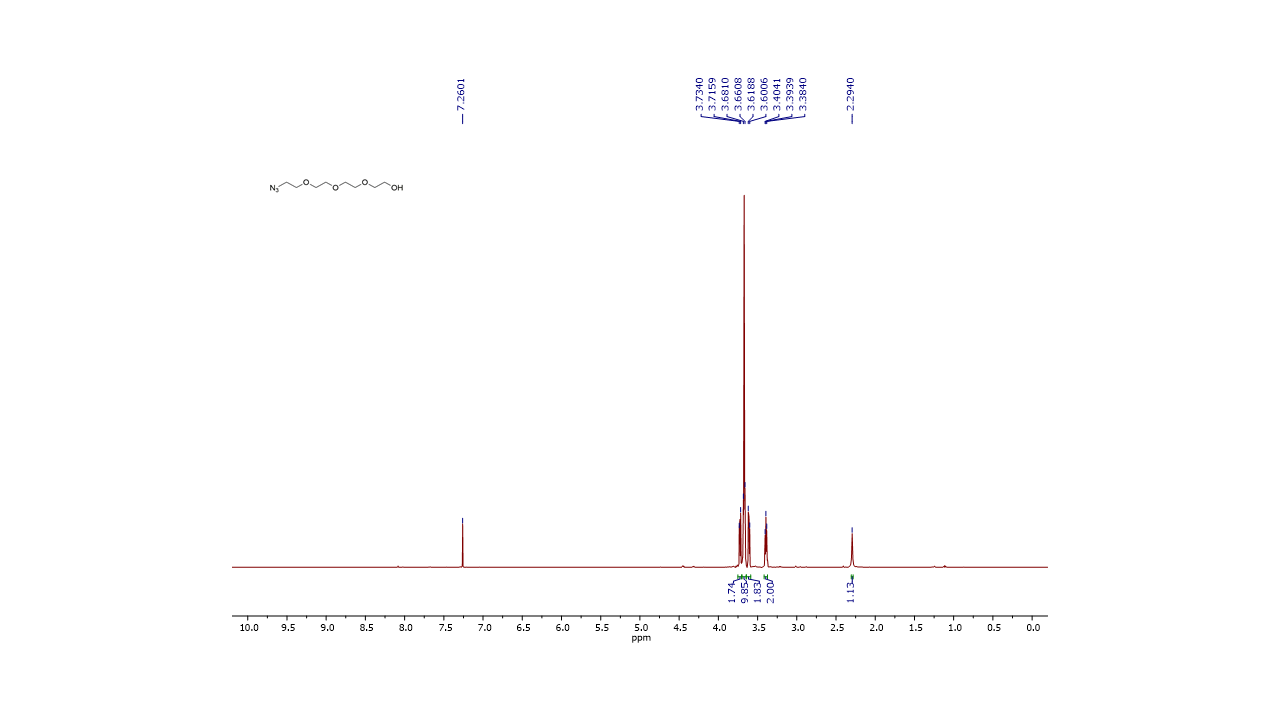


**Figure S15**: ^1^H NMR spectrum of **6** in CDCl_3_.


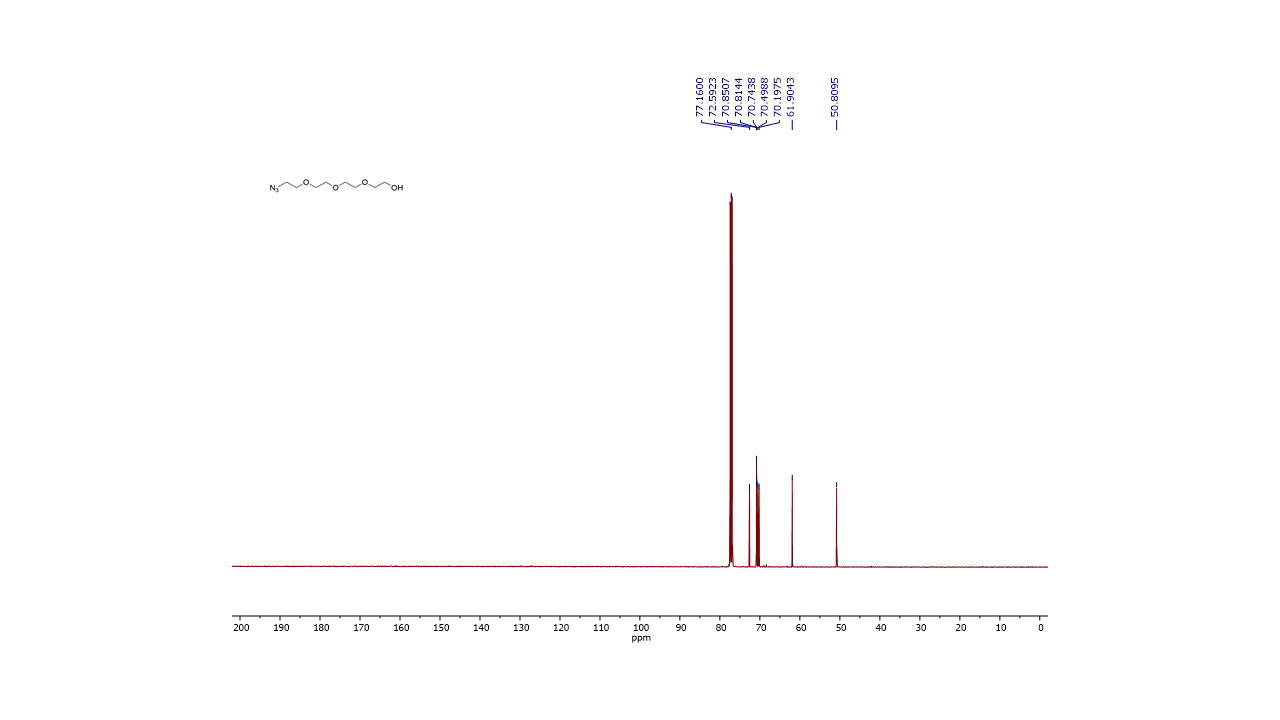


**Figure S16**: ^13^C NMR spectrum of **6** in CDCl_3_.


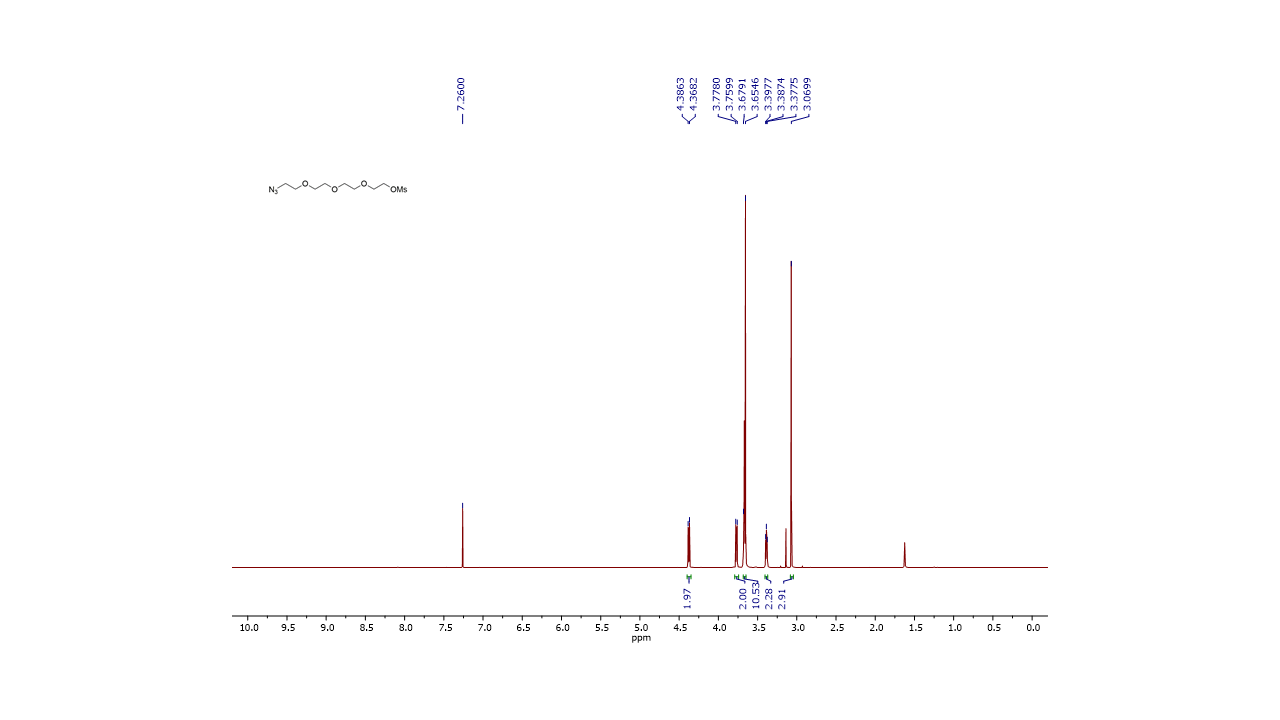


**Figure S17:** ^1^H NMR spectrum of **7** in CDCl_3_.


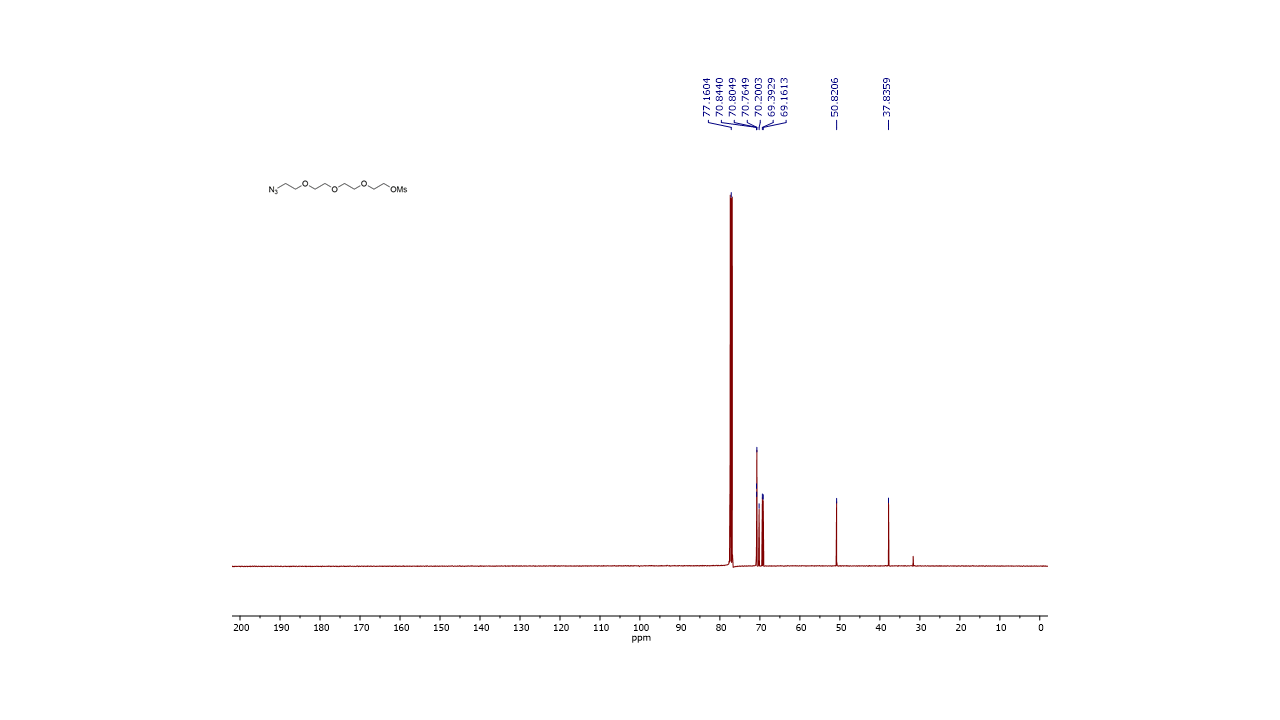


**Figure S18:** ^13^C NMR spectrum of **7** in CDCl_3_.


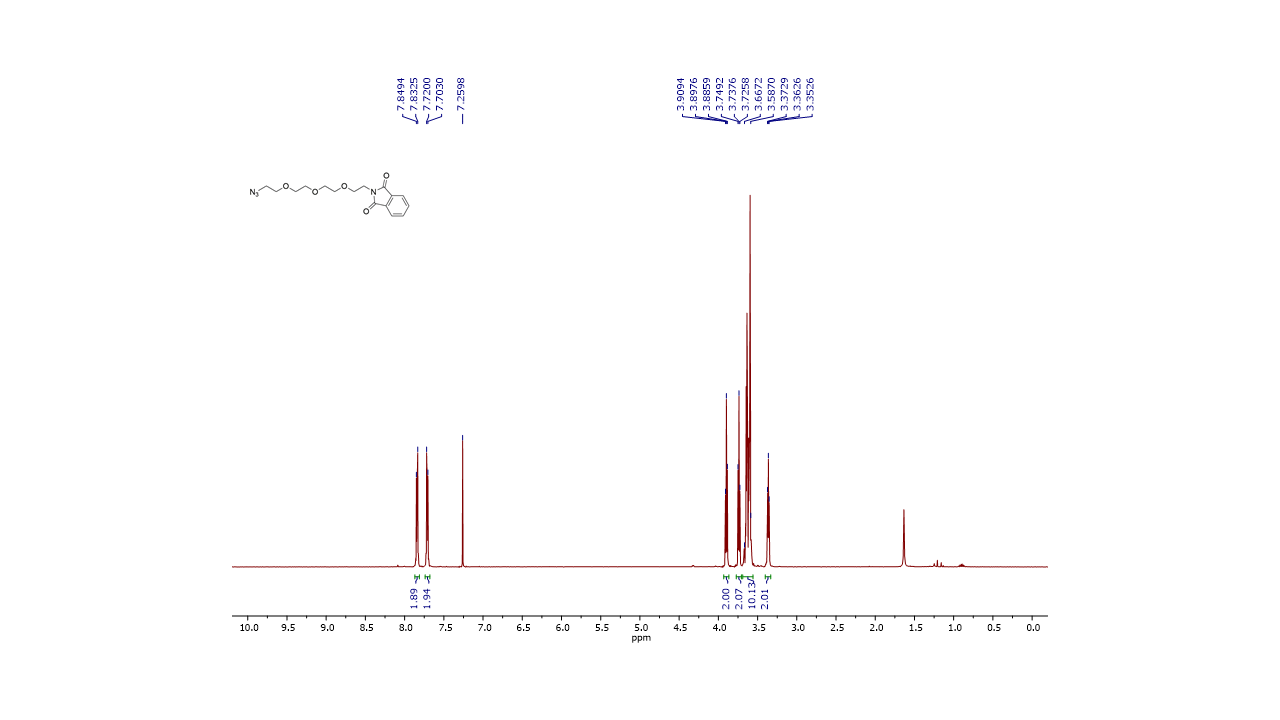


**Figure S19:** ^1^H NMR spectrum of **8** in CDCl_3_.


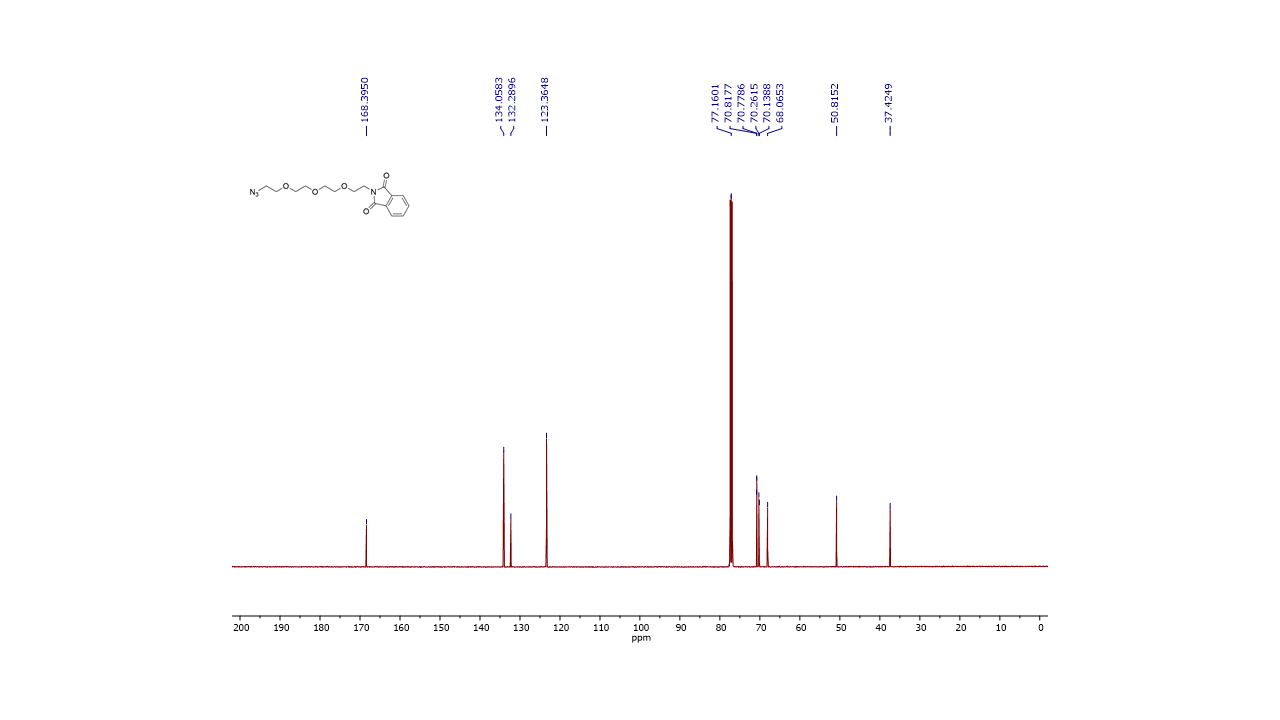


**Figure S20:** ^13^C NMR spectrum of **8** in CDCl_3_.


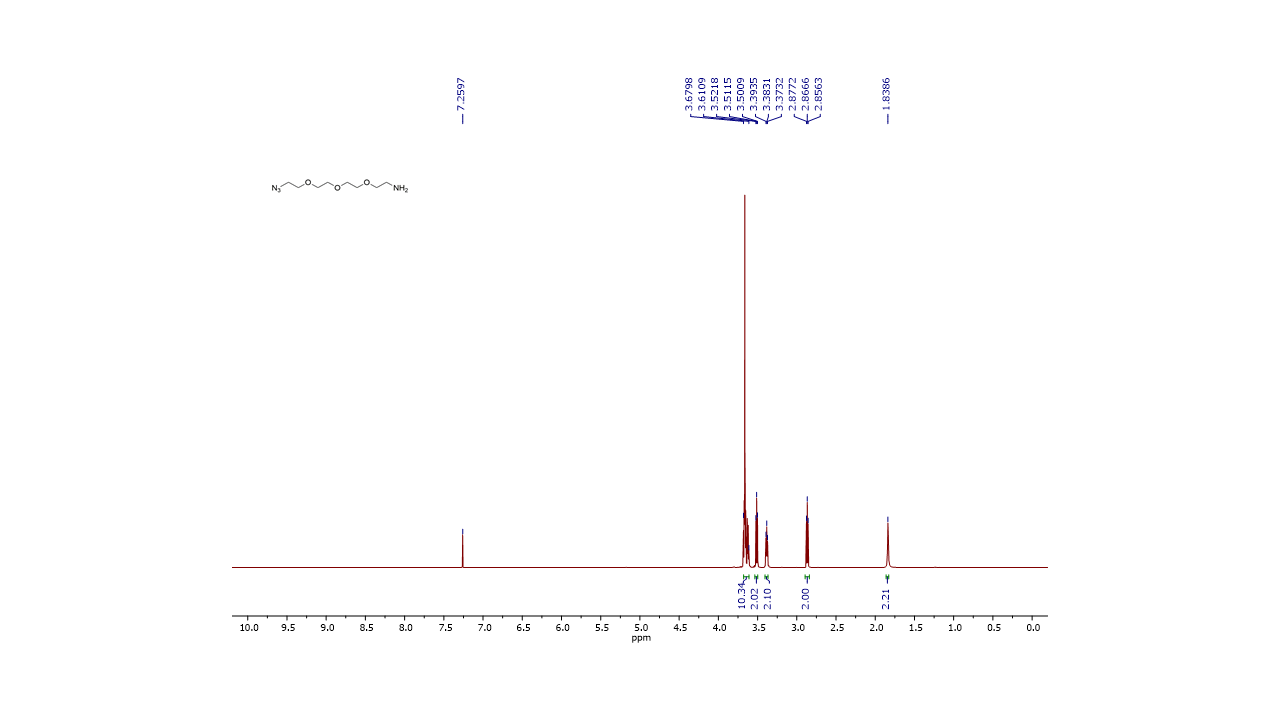


**Figure S21:** ^1^H NMR spectrum of **9** in CDCl_3_.


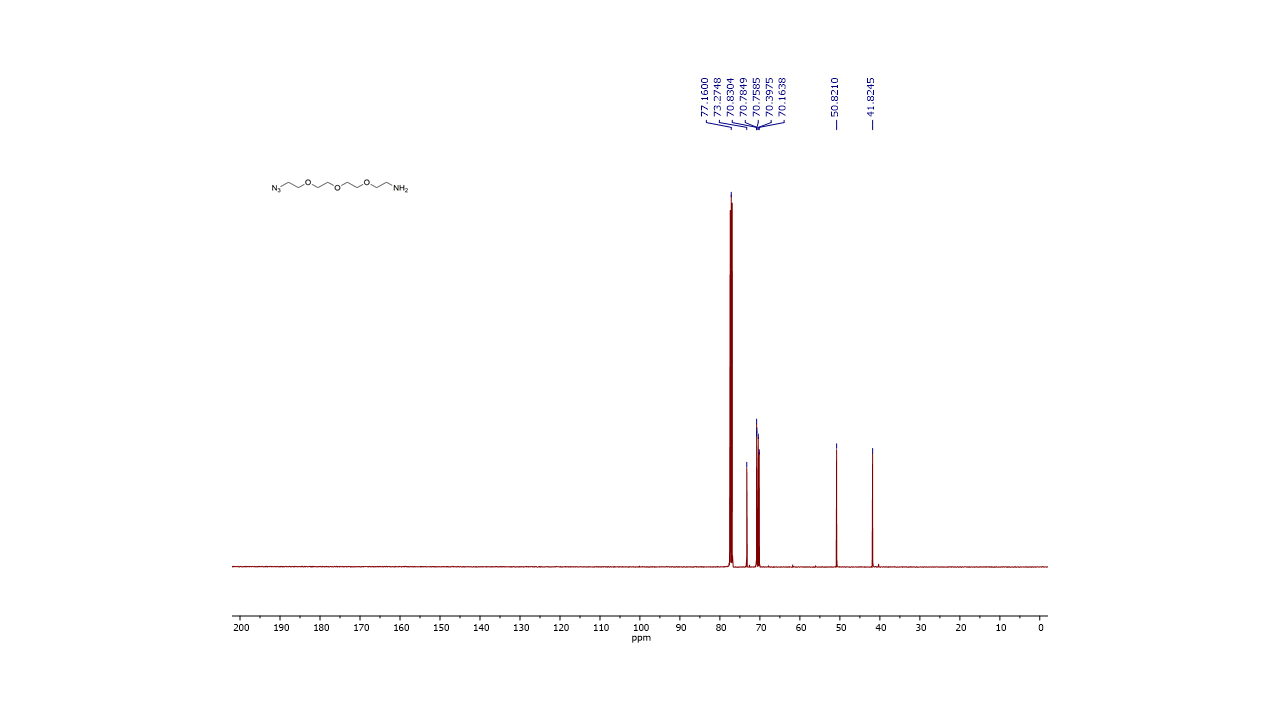


**Figure S22:** ^13^C NMR spectrum of **9** in CDCl_3_.


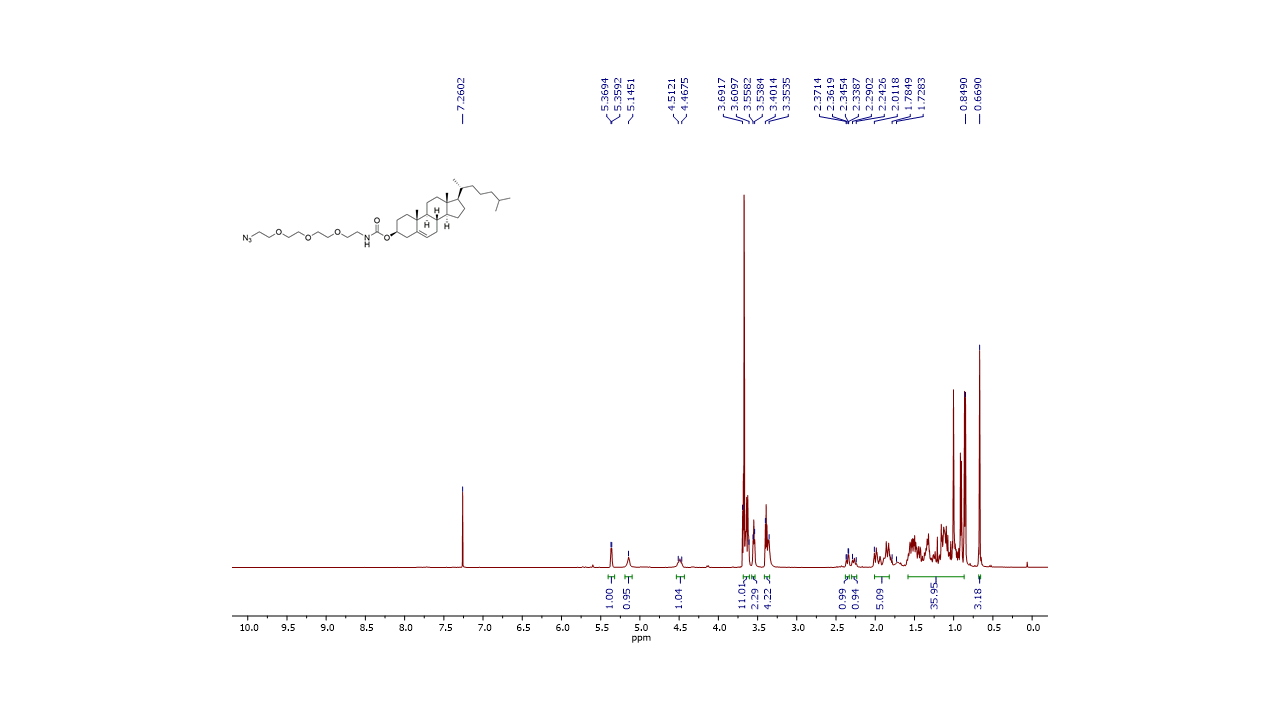


**Figure S23:** ^1^H NMR spectrum of **10** in CDCl_3_.


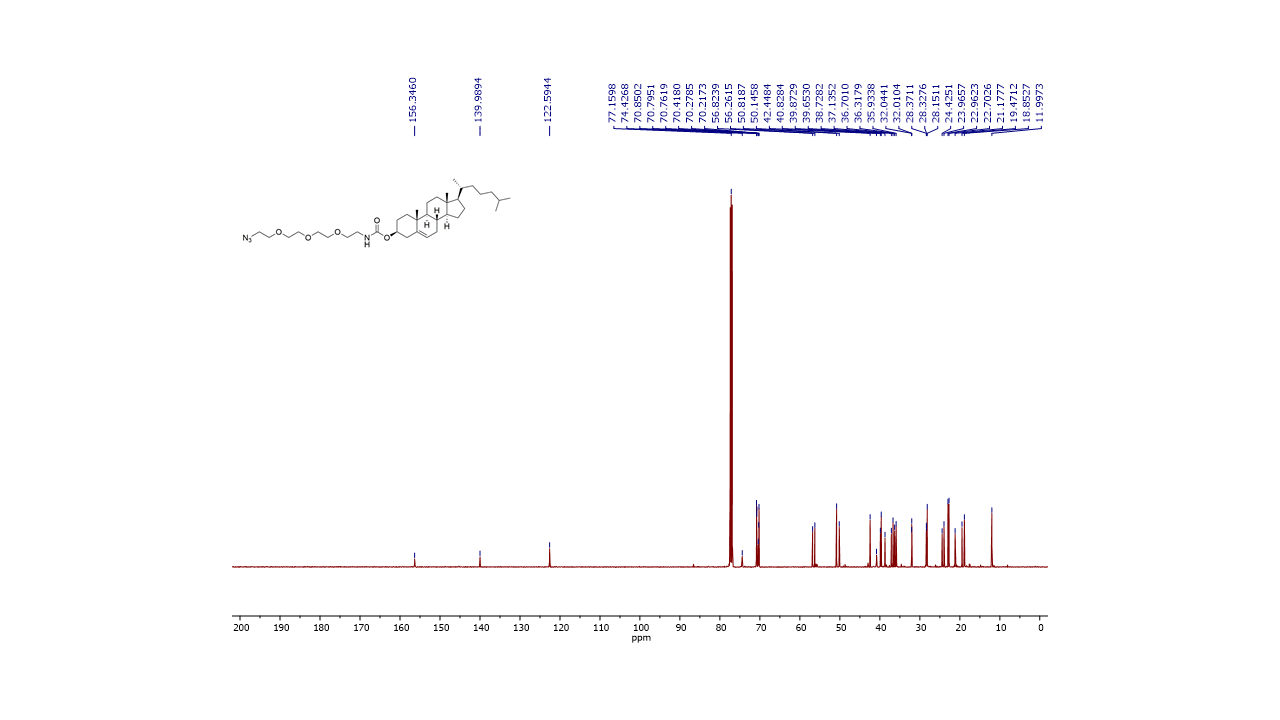


**Figure S24:** ^13^C NMR spectrum of **10** in CDCl_3_.


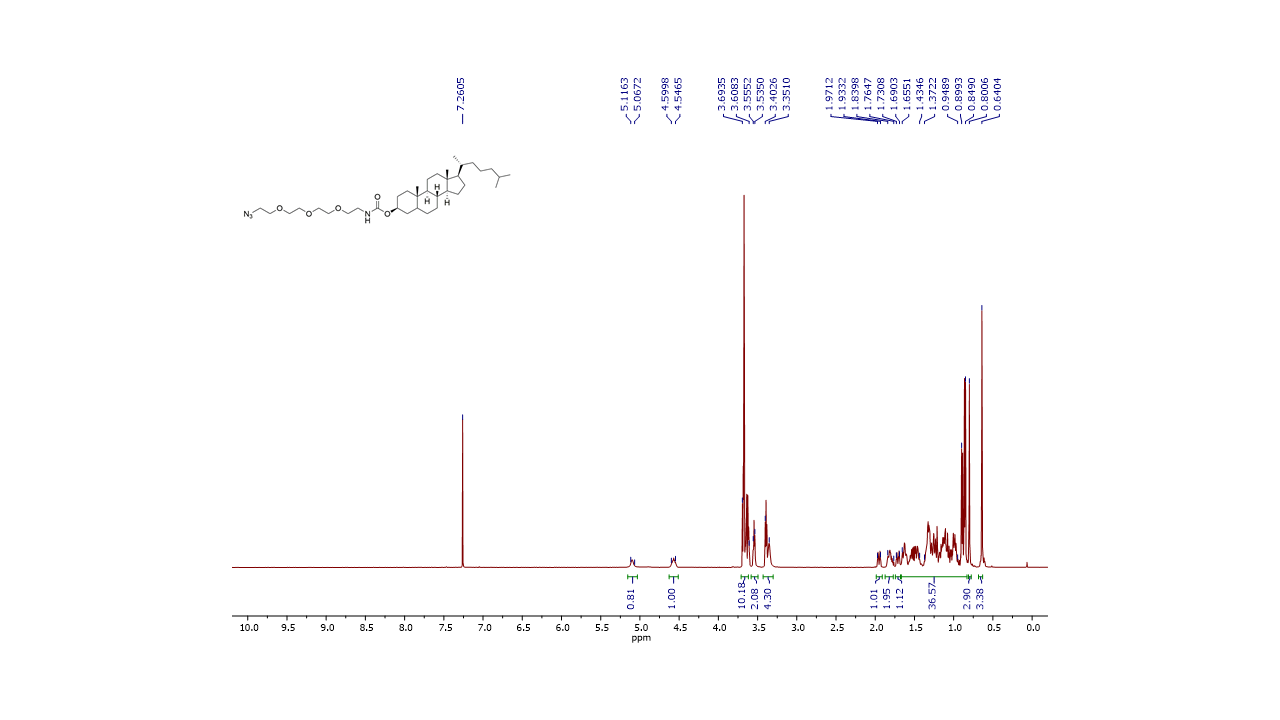


**Figure S25:** ^1^H NMR spectrum of **11** in CDCl_3_.


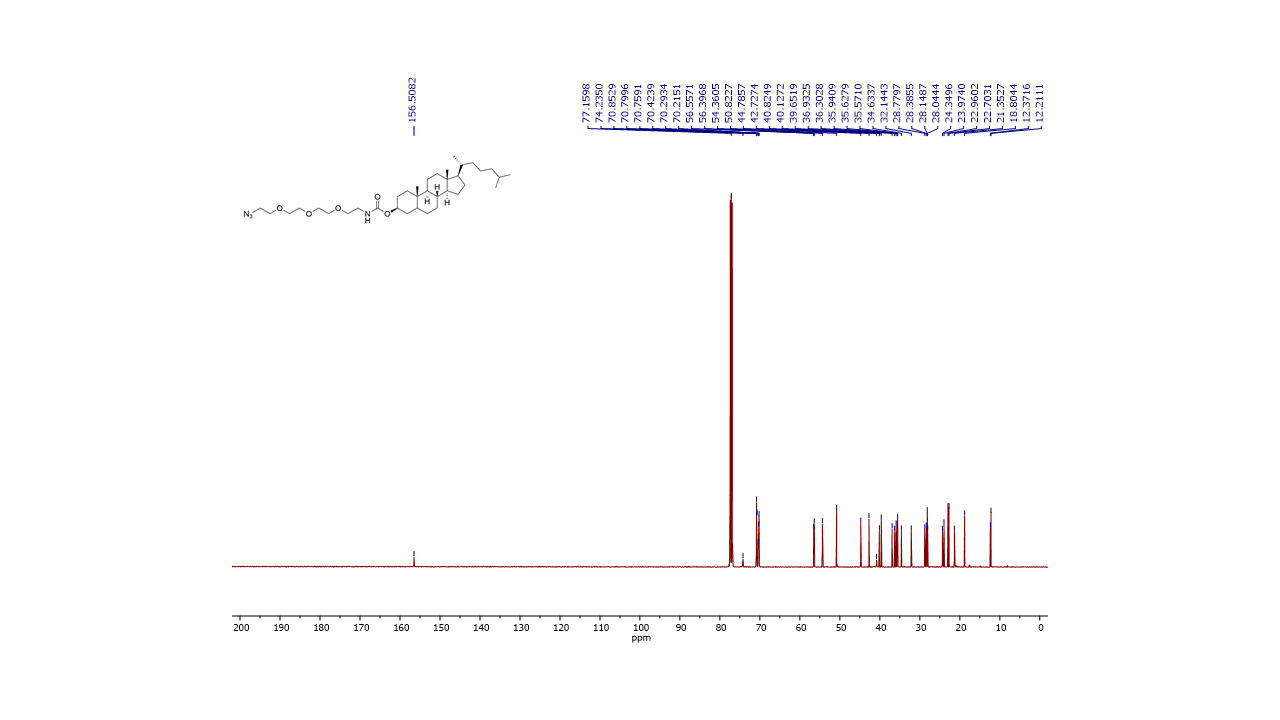


**Figure S26:** ^13^C NMR spectrum of **11** in CDCl_3_.


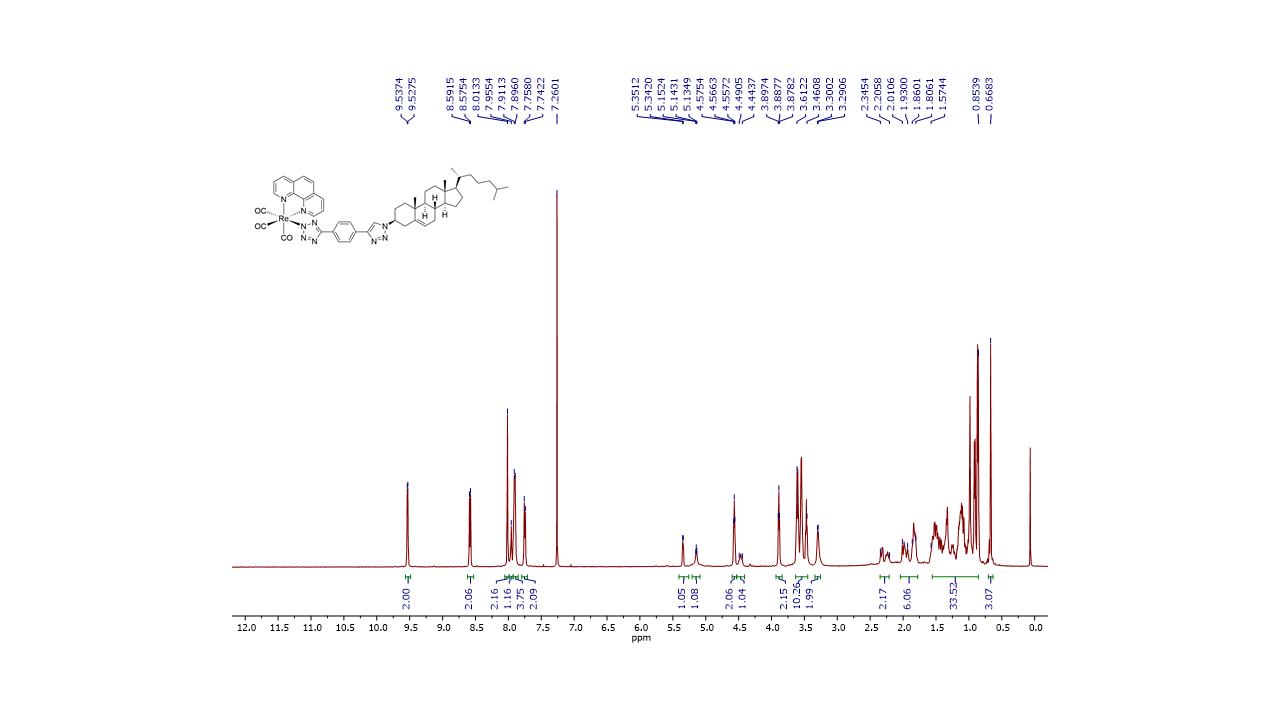


**Figure S27:** ^1^H NMR spectrum of **ReTEGCholesterol** in CDCl_3_.


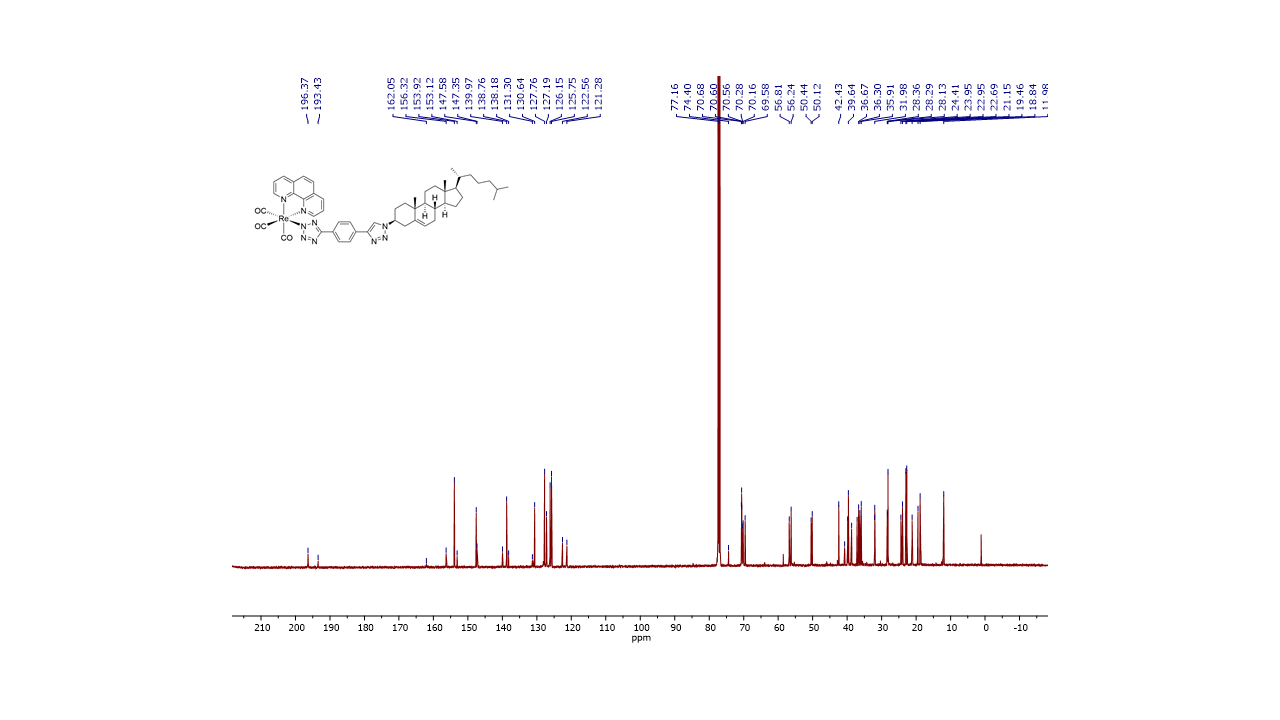


**Figure S28:** ^13^C NMR spectrum of **ReTEGCholesterol** in CDCl_3_.


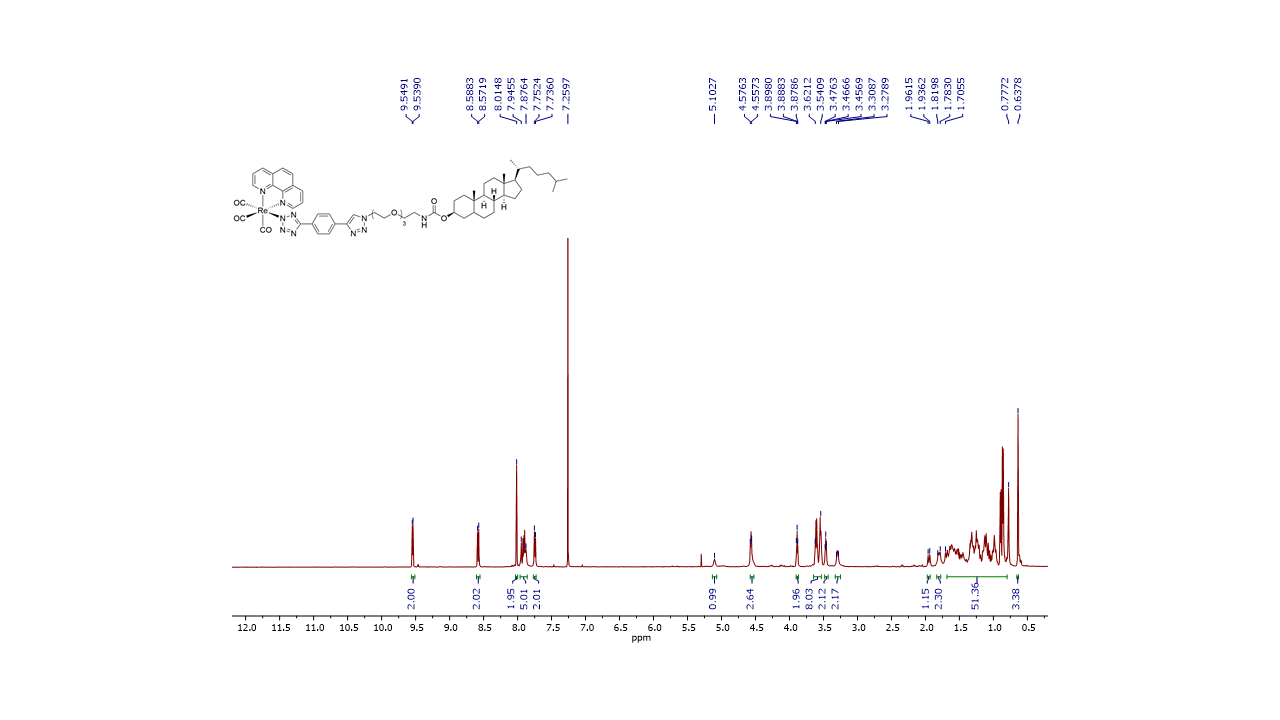


**Figure S29:** ^1^H NMR spectrum of **ReTEGCholestanol** in CDCl_3_.


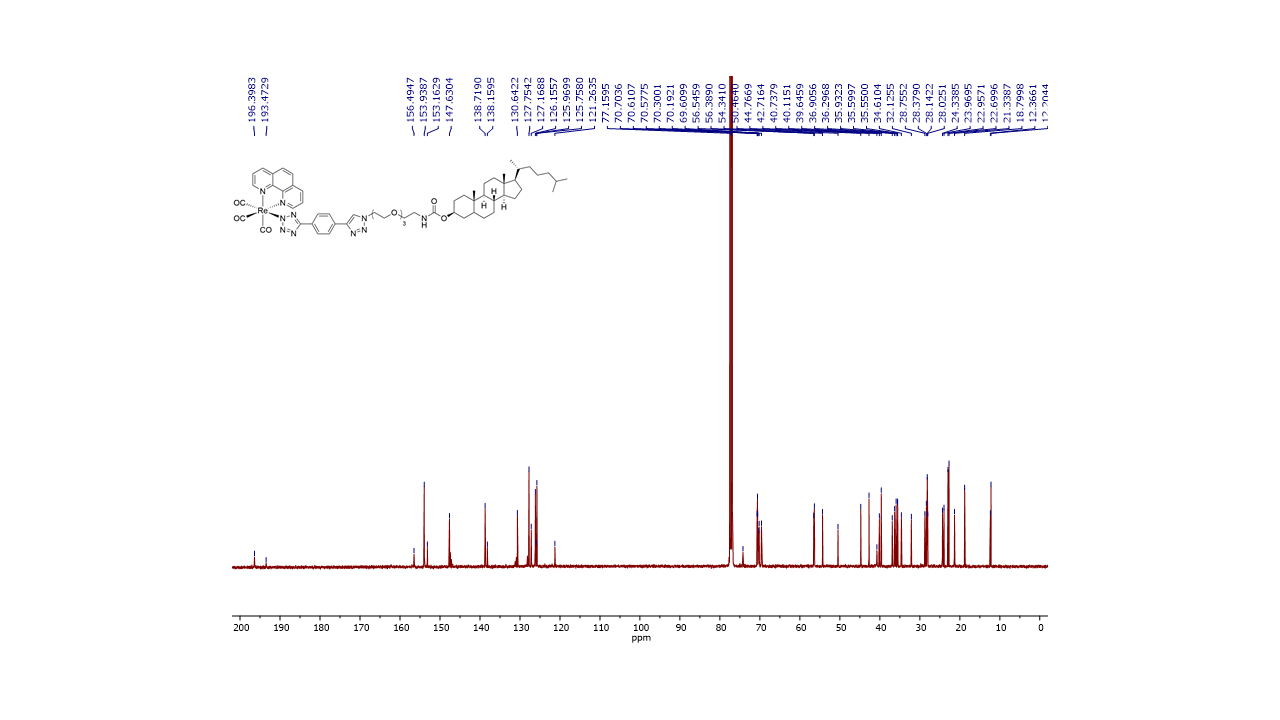


**Figure S30:** ^13^C NMR spectrum of **ReTEGCholestanol** in CDCl_3_.

**Figure S31**: HRMS spectrum of **ReCholesterol**.

**Figure S32**: HRMS spectrum of **ReCholestanol**.

**Figure S33:** HRMS spectrum of **ReTEGCholesterol**.


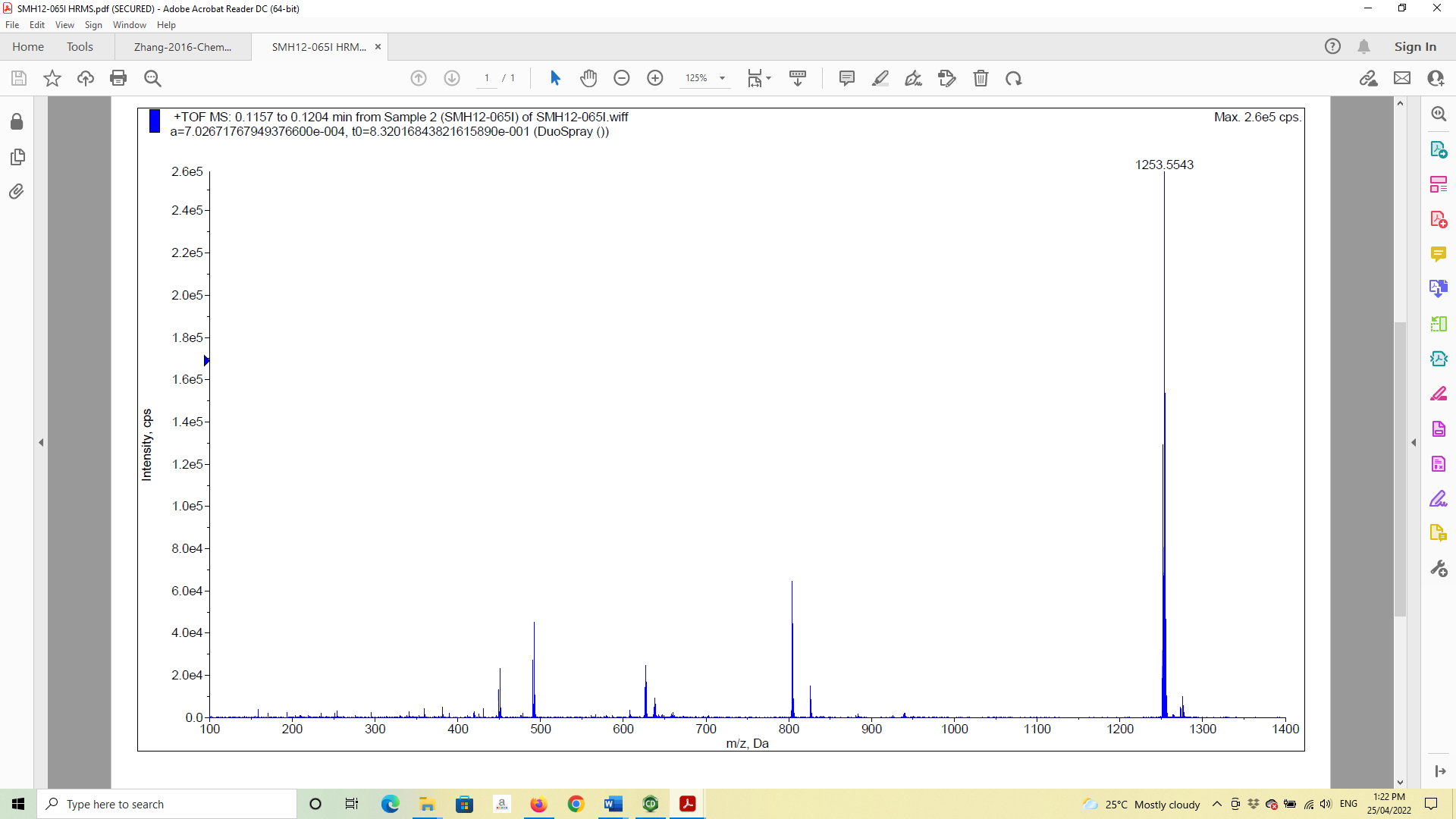


**Figure S34**: HRMS spectrum of **ReTEGCholestanol**


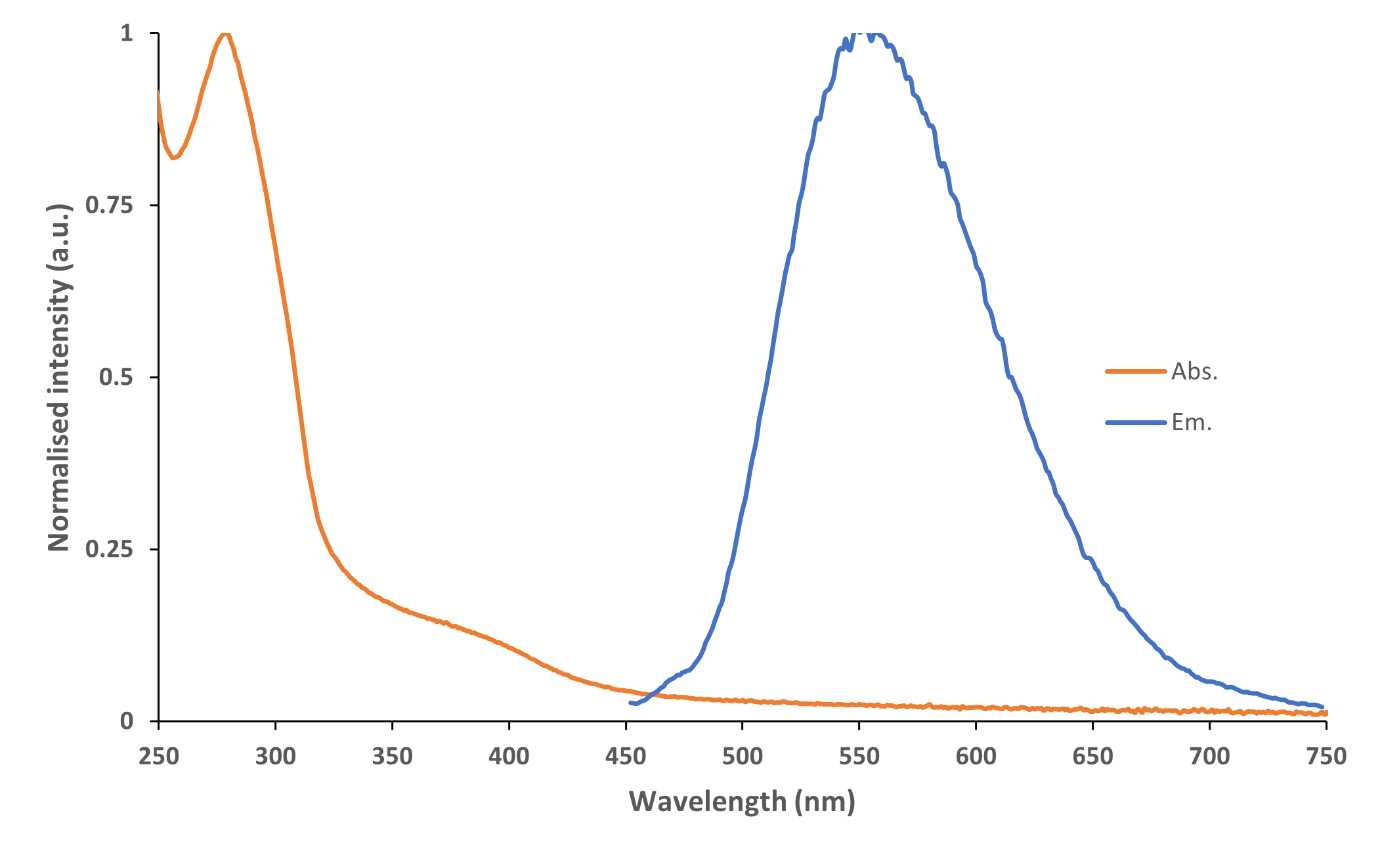


**Figure S35**: Normalised absorbance and fluorescence emission spectra for **ReCholesterol**.


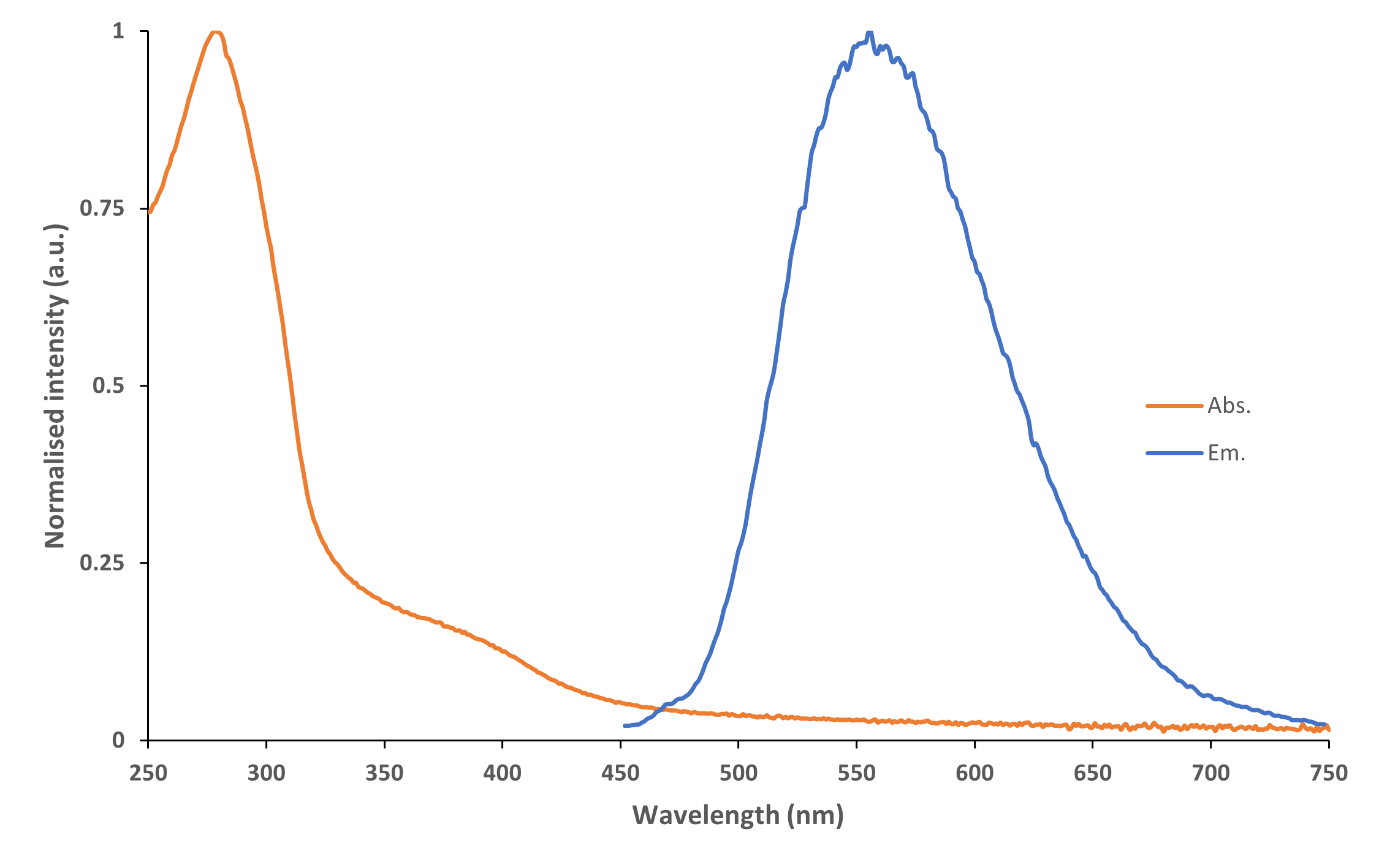


**Figure S36**: Normalised absorbance and fluorescence emission spectra for **ReCholestanol**.


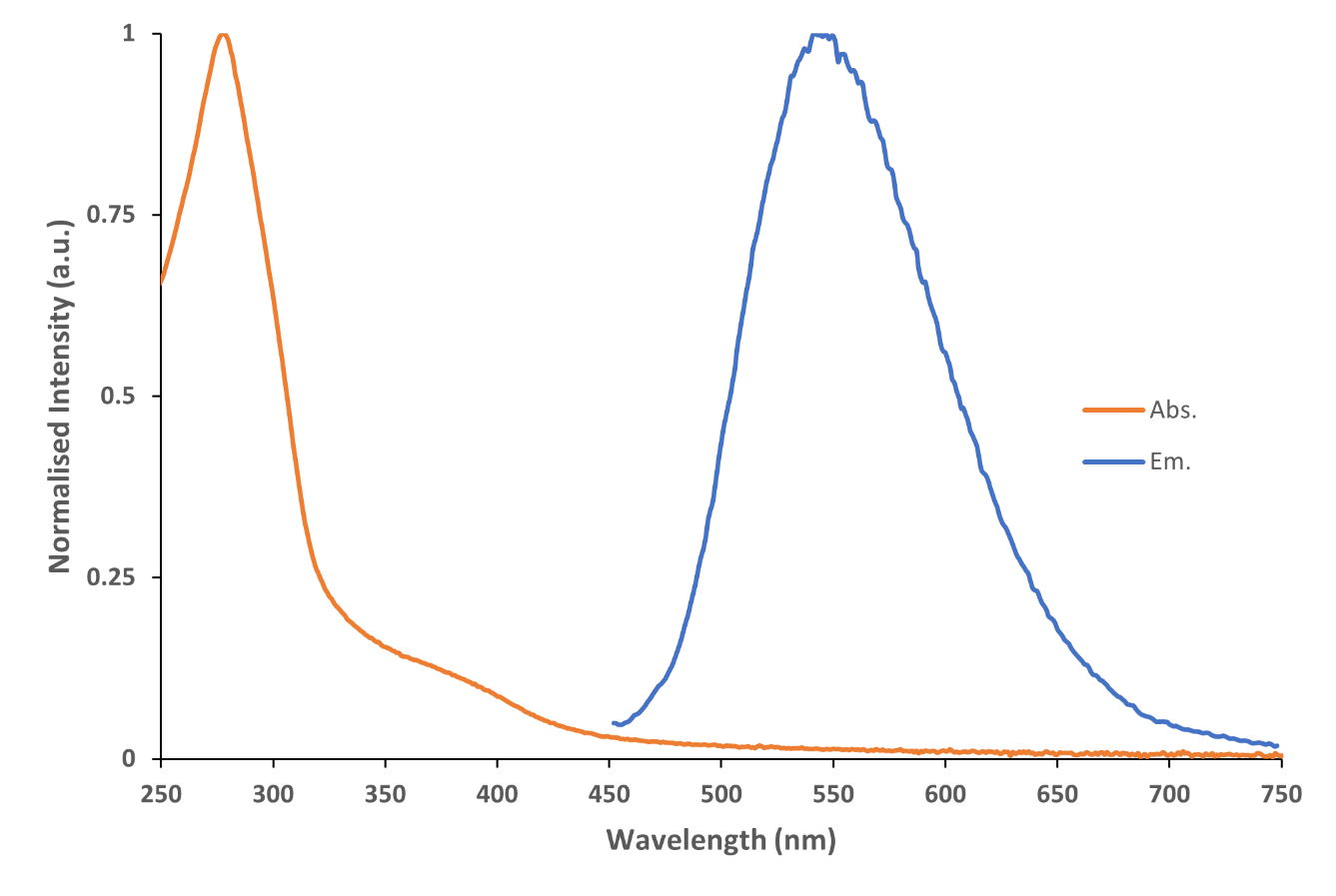


**Figure S37**: Normalised absorbance and fluorescence emission spectra for **ReTEGCholesterol**.


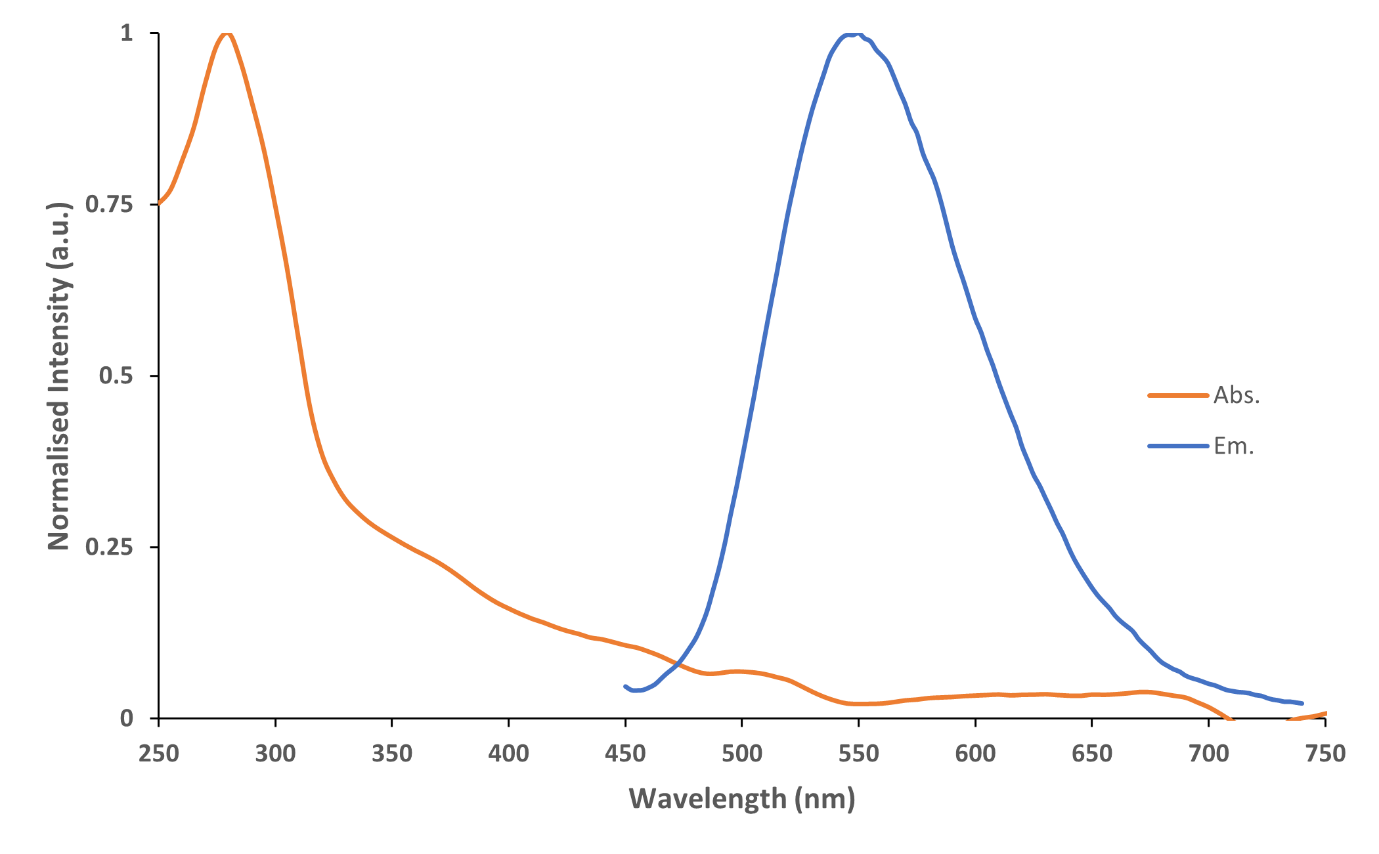


**Figure S38**: Normalised absorbance and fluorescence emission spectra for **ReTEGCholestanol**.
